# Biological and therapeutic implications of a unique subtype of *NPM1* mutated AML

**DOI:** 10.1038/s41467-021-21233-0

**Published:** 2021-02-16

**Authors:** Arvind Singh Mer, Emily M. Heath, Seyed Ali Madani Tonekaboni, Nergiz Dogan-Artun, Sisira Kadambat Nair, Alex Murison, Laura Garcia-Prat, Liran Shlush, Rose Hurren, Veronique Voisin, Gary D. Bader, Corey Nislow, Mattias Rantalainen, Soren Lehmann, Mark Gower, Cynthia J. Guidos, Mathieu Lupien, John E. Dick, Mark D. Minden, Aaron D. Schimmer, Benjamin Haibe-Kains

**Affiliations:** 1 Princess Margaret Cancer Centre, University Health Network, Toronto, ON Canada; 2 Department of Medical Biophysics, University of Toronto, Toronto, ON Canada; 3 Karolinska Institute, Stockholm, Sweden; 4 Department of Immunology, Weizmann Institute of Science, Rehovot, Israel; 5 The Donnelly Centre, University of Toronto, Toronto, ON Canada; 6 Department of Computer Science, University of Toronto, Toronto, ON Canada; 7 Faculty of Pharmaceutical Sciences, The University of British Columbia, Vancouver, Canada; 8 The Hospital for Sick Children, Toronto, ON Canada; 9 Ontario Institute for Cancer Research, Toronto, ON Canada; 10 Department of Molecular Genetics, University of Toronto, Toronto, ON Canada; 11 Vector Institute, Toronto, ON Canada

**Keywords:** Acute myeloid leukaemia, Machine learning, Biomarkers, Prognostic markers

## Abstract

In acute myeloid leukemia (AML), molecular heterogeneity across patients constitutes a major challenge for prognosis and therapy. AML with *NPM1* mutation is a distinct genetic entity in the revised World Health Organization classification. However, differing patterns of co-mutation and response to therapy within this group necessitate further stratification. Here we report two distinct subtypes within *NPM1* mutated AML patients, which we label as primitive and committed based on the respective presence or absence of a stem cell signature. Using gene expression (RNA-seq), epigenomic (ATAC-seq) and immunophenotyping (CyToF) analysis, we associate each subtype with specific molecular characteristics, disease differentiation state and patient survival. Using ex vivo drug sensitivity profiling, we show a differential drug response of the subtypes to specific kinase inhibitors, irrespective of the *FLT3-ITD* status. Differential drug responses of the primitive and committed subtype are validated in an independent AML cohort. Our results highlight heterogeneity among *NPM1* mutated AML patient samples based on stemness and suggest that the addition of kinase inhibitors to the treatment of cases with the primitive signature, lacking *FLT3-ITD*, could have therapeutic benefit.

## Abstract (web summary)

Molecular heterogeneity of acute myeloid leukaemia (AML) across patients is a major challenge for prognosis and therapy. Here, the authors show that NPM1 mutated AML is a heterogeneous class, consisting of two subtypes which exhibit distinct molecular characteristics, differentiation state, patient survival and drug response.

## Introduction

Acute myeloid leukemia (AML) is a genetically and biologically heterogeneous disease characterized by the clonal expansion and impaired differentiation of mutant hematopoietic stem and progenitor cells^1^. Among the most common AML driver mutations, stable over time, is a 4 base-pair insertion in exon 12 of the nucleophosmin-1 (*NPM1*) gene, occurring in 20–30% of cases^2,3^. Due to its biological significance and prognostic impact, mutations in *NPM1* represent a distinct leukemic entity in the World Health Organization (WHO) classification of myeloid leukemias and play a significant role in prognosis and treatment decision-making^4^.

*NPM1* mutations are generally associated with a favorable effect on patient survival following induction and consolidation chemotherapy^5^. However, AML with *NPM1* mutation is a clinically heterogeneous group because it almost always exists in the context of other mutations. For example, internal tandem duplications in *FLT3* (*FLT3-ITD*) are approximately twice as frequent in *NPM1*-mutated AML compared to AML with wild-type *NPM1*^6,7^. Such secondary mutations have functional consequences. For example, patients with *NPM1* mutations in the absence of *FLT3-ITD* have a more favorable prognosis, than patients with the *FLT3-ITD*, and are usually not offered allogeneic stem cell transplant^8^. Yet, even among this favorable group, 40% of patients will relapse, indicating unrecognized heterogeneity in this subgroup^6,9^; this may be due to additional genetic and epigenetic changes, contributing to the prognosis of *NPM1*-mutated disease^2,10^. For example, within *NPM1*-mutated AML, the *FLT3-ITD* mutation frequently co-occurs with mutations of *DNMT3A*, which on its own is associated with worse outcome in patients receiving standard induction therapy^11^. Most studies of *NPM1-*mutated AML have focused on the co-occurrence of other mutations, while heterogeneity at a gene expression level among patients with mutant *NPM1*, and its biological significance have not been comprehensively investigated yet.

In this work, we used RNA-seq-based gene expression profiling to characterize the molecular heterogeneity within *NPM1*-mutated AML patients. As a result, we identify two subtypes, referred to as primitive and committed based on the differences in gene expression, and find that each is consistently associated with particular molecular characteristics, disease differentiation state and patient survival across multiple independent AML cohorts. Furthermore, we show that leukemic cells in the primitive subtype are more sensitive to certain kinase inhibitors, even in the absence of *FLT3-ITD*. This suggests that the addition of kinase inhibitors to the treatment of cases with the primitive signature, lacking *FLT3-ITD*, may be of therapeutic benefit.

In this work, we characterize the molecular heterogeneity within NPM1-mutated AML patients. Using RNA-seq-based gene expression profiling we identify two novel subtypes, referred to as primitive and committed. Based on the differences in gene expression, epigenomic (ATAC-seq), and immunophenotyping (CyToF), we associate subtypes with particular molecular characteristics, disease differentiation state and patient survival across multiple independent AML cohorts. Furthermore, we show that leukemic cells in the primitive subtype are more sensitive to certain kinase inhibitors, even in the absence of FLT3-ITD. This suggests that the addition of kinase inhibitors to the treatment of cases with the primitive signature, lacking FLT3-ITD, may be of therapeutic benefit.

## Results

### *NPM1*-mutated AML clusters into two distinct groups

**Fig. 1:**
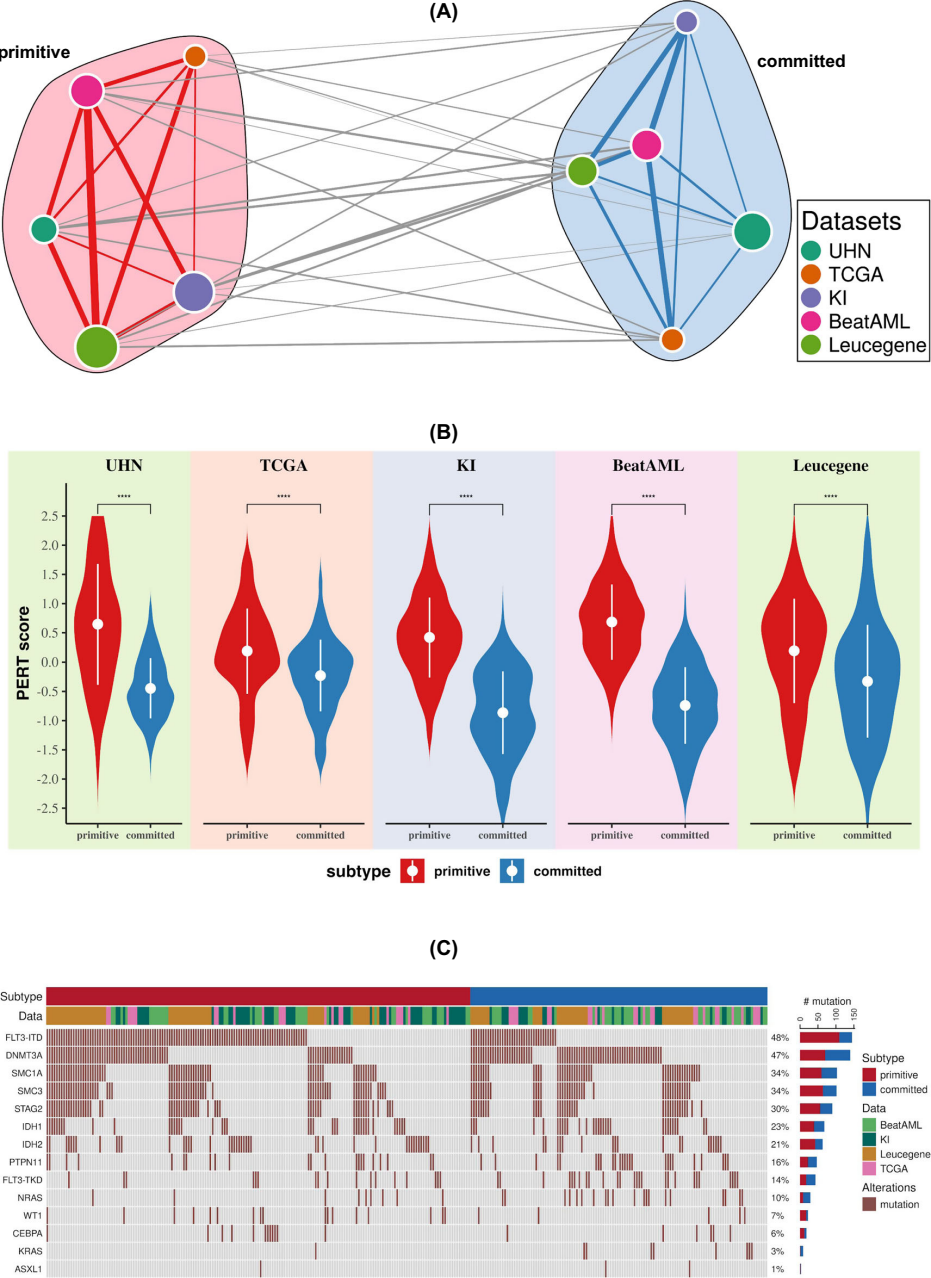
*NPM1* mutated AML patients can be classified into two distinct molecular subtypes. **A** Consensus clustering of gene expression data shows two distinct clusters across five different datasets. Unsupervised machine-learning method was applied to five different patient cohorts independently and an optimal number of clusters (two clusters, supplementary Fig. 1) were discovered in each cohort. In the network, each node represents a cluster from a dataset. The size of the nodes are proportional to the number of the patients in the cluster and are colored according to the dataset. To compare the clusters from different datasets, Pearson correlation coefficients between their centroids was used. In the network, edge width is proportional to the correlation between clusters. This network was further classified into two clusters (meta-clustering), annotated as primitive and committed. For network visualization, Fruchterman–Reingold force directed layout algorithm was applied. **B** The cellular deconvolution shows that primitive clusters are enriched in stem cells (two-sided Wilcoxon rank-sum test *p*-value < 2.2E−16 for UHN, KI, BeatAML, Leucegene dataset, and *p*-value = 4.5E−14 for TCGA dataset). PERT algorithm was applied to compute the stem cell score for all samples. Higher PERT score indicates that the subtype is enriched with stem cells. **C** Mutation status of genes in primitive and committed clusters. In the oncoprint, the top bar indicates primitive and committed subtype and the second bar shows patient cohort.

We investigated whether analysis of gene expression patterns might identify molecular subtypes of *NPM1-*mutated AML. To define consensus molecular subtypes across a large compendium of 391 RNA-sequencing profiles of *NPM1*-mutated AML samples, we applied a meta-clustering approach using the CoINcIDE^12^ framework (Supplementary Table 1, Supplementary Fig. 1 and Supplementary Methods). Our meta-clustering analysis revealed two robust subtypes across our data compendium (Fig. 1A and Supplementary Figs. 1 and 2). Next, the PERT algorithm^13^ was used to elucidate the cellular composition of the AML samples in each cluster. We found that one cluster was significantly enriched for stem cells, hence labeled as *primitive*. In contrast, the other cluster was enriched for gene expression associated with myeloid and hematopoietic differentiation, hence we labeled it *committed* (Fig. 1B). The two clusters did not differ in clinicopathological parameters such as age, karyotypem and white blood cell counts (Chi-square test false discovery rate [FDR] > 5%; Supplementary Tables 2–5). We also investigated the distribution of key driver mutations in the primitive and committed subtypes (Fig. 1C, “Subtype and mutations” section in Supplementary Discussion and Supplementary Tables 6–17). Although subtypes were enriched with certain mutations (*FLT3-ITD* in primitive and *DNMT3A* in the committed group), genetic alterations in driver mutations are poorly predictive of the committed and primitive subtypes (Supplementary Figs. 3–16 and Supplementary Discussion) with low Matthews correlation coefficient (MCC) between gene mutations and subtypes (MCC = 0.32 for *FLT3-ITD* and MCC = −0.16 for *DNMT3A*; Supplementary Fig. 3). Multivariate analysis between mutation and subtype achieved a weak area under precision-recall curve (AUPRC = 0.62) value (Supplementary Fig. 7).

### Molecular basis of clusters of *NPM1*-mutated AML patients

**Fig. 2:**
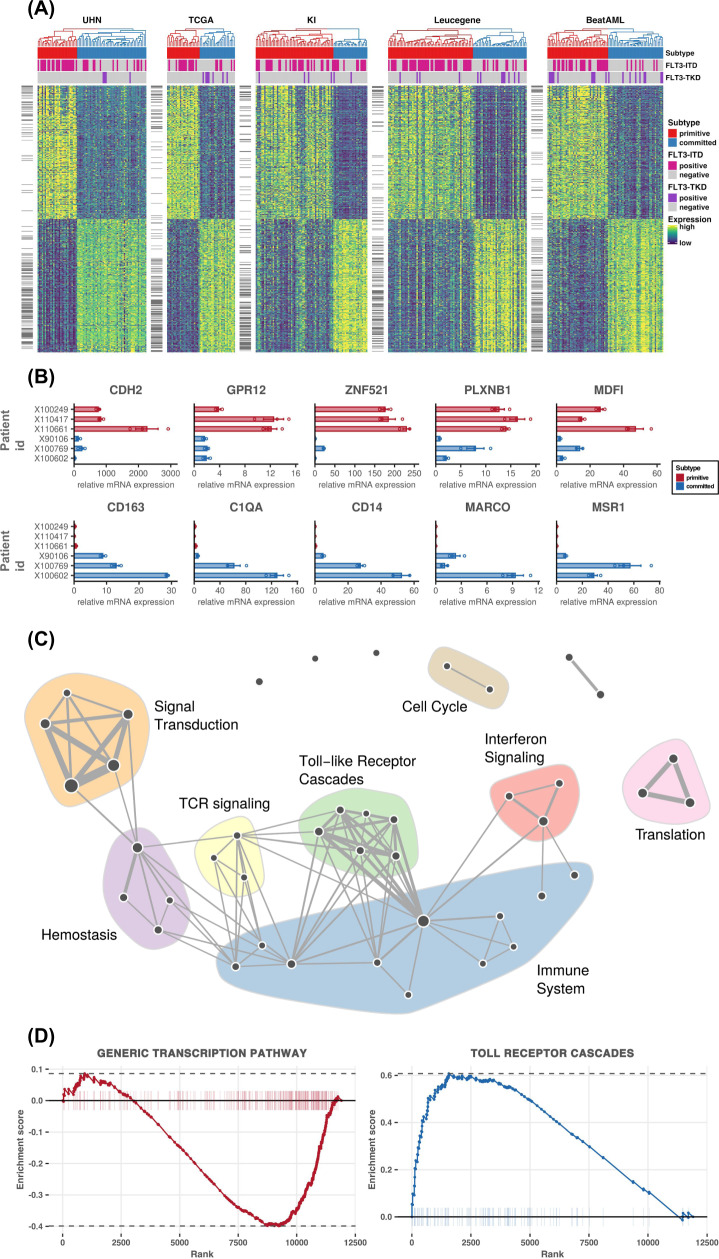
Gene and pathway level analysis elucidates molecular differences between subtypes. **A** Gene expression pattern across five AML cohorts. Patients are represented in columns while genes are represented in rows. The genes that are differentially expressed across all the datasets are shown on the right side of the heatmap with a short horizontal black bar. **B** The expression level of selected genes were confirmed using qPCR analysis (*n* = 6 patient samples, each with three replicates). Bars show relative expression of genes in samples belonging to primitive (in red color bars) and committed (in blue color bars) subtypes. Data are represented as relative mRNA expression and as mean ± SEM. In the primitive subtype samples, *CDH2*, *GPR12*, *ZNF521*, *PLXNB1*, and *MDFI* have high expression. In the committed subtype *CD163, C1QA, CD14, MARCO,* and *MSR1* show high levels of expression. **C** Network visualization for gene set enrichment analysis showing pathways with enrichment FDR <5%. Each node in the network represents a pathway and the edge represents common genes between pathways. Size of the nodes and edges are proportional to the number of genes in the pathway and common genes, respectively. For individual pathway names see Supplementary Fig. S18. **D** Pathway enrichment plot for transcription and Toll receptor cascade pathway. Plots show the running enrichment score and positions of the genes belonging to the pathway in the rank order list of all genes. The transcription pathway is enriched in the primitive subtype while the Toll receptor cascade pathway is enriched in the committed subtype.

We further explored the transcriptomic patterns specific to each subtype by identifying the genes that are consistently differentially expressed across our compendium of RNA-sequencing datasets using the DESeq2 method^14^ in a meta-analysis framework (Fig. 2A and Supplementary Fig. 8). The differential analysis shows that the cadherin-2 (*CDH2*) gene is significantly upregulated in the primitive subtype (Fig. 2B and Supplementary Data 1). A member of the cadherin family, *CDH2* is known to be a regulator of stem cell fate decisions^15^. Similarly, G protein-coupled receptor 12 (*GPR12*) which is upregulated in the primitive subtype, is known to play a role in stem cell maintenance and somatic reprogramming of cancer stem cells^16^. The MyoD family inhibitor (*MDFI*), increasingly expressed in the primitive subtype, has been reported to be a regulator of *WNT* signaling pathway and is exclusively expressed in hematopoietic stem progenitor cells^17^. Interestingly, zinc finger protein 521 (*ZNF521*), a transcription factor whose knockdown has been shown to reduce proliferation in human leukemia cell lines^18^ had significantly higher expression in the primitive subtype.

In the committed subtype, we found an upregulated expression of *CD163*, which has been associated with monocytic differentiation^19,20^. *CD163* is an immunomodulator and member of the macrophage scavenger receptor family, known to be expressed by AML cells of monocytic lineage^21^. Higher expression of other genes in the committed cluster includes immune-related genes such as *C1QA*, *CD14,* and *MARCO*. The *MSR1* gene, a known suppressor of leukemia stem cell proliferation, is also highly expressed in the committed cluster^22^. We confirmed the differential expression of key genes by qPCR (Fig. 2B). Using gene set enrichment analysis (GSEA) we detected that in the committed subtype, immune response pathways such as interferon-gamma-mediated signaling, GPCR signaling, and toll-like receptor (TLR) signaling are upregulated (Fig. 2C, D, Supplementary Figs. 16, 18, and Supplementary Data 2). Concurring with the weak association with *FLT3-ITD* and *DNMT3A* mutations, we found that the differential gene expressions and enriched pathways are uniquely activated in subtypes (Supplementary Figs. 8–16).

### Primitive phenotype and chromatin accessibility

**Fig. 3:**
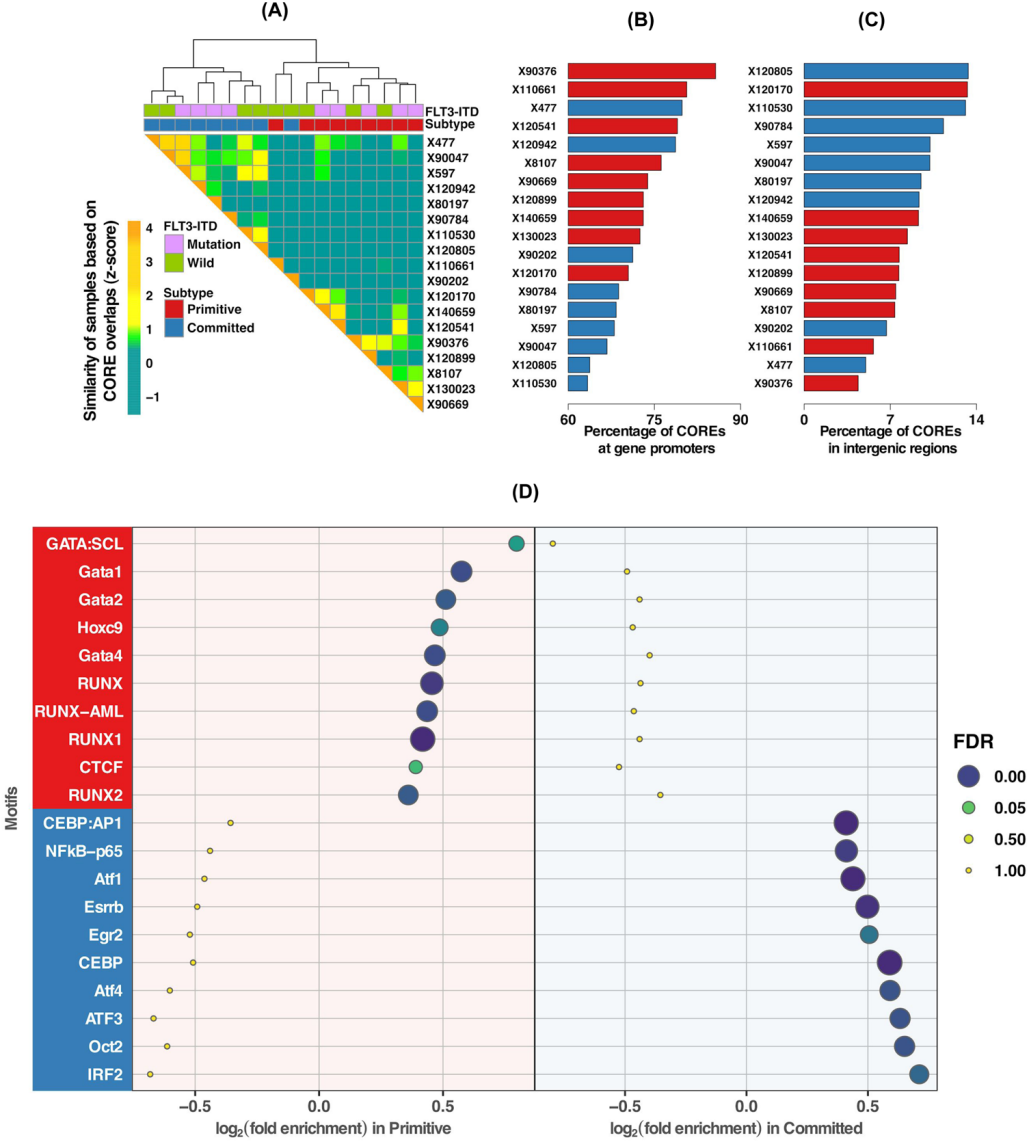
Cis-regulatory landscape of AML samples shows high degree of agreement with gene expression based phenotype (primitive or committed). **A** Clustering of AML samples using the called COREs from ATAC-seq profiles, groups samples into two clusters. These clusters are highly concordant with gene expression-based primitive or committed subtypes. Comparison of distribution of AML COREs at (**B**) promoters and (**C**) intergenic regions within the human genome. COREs are enriched in the promoter regions of the primitive subtype. For the committed subtype, COREs are more enriched in the intergenic regions. **D** Dotplot visualization of top 10 enriched motifs for transcription factor-binding sites (TFBS) in primitive and committed subtypes. The *x*-axis represents the enrichment score, *y*-axis shows names of the TFBS motif and dots’ size and color is proportional to FDR. Motif names with red background (on *y*-axis) have a significant enrichment score (FDR < 0.05) in primitive subtype and with blue have significant enrichment score (FDR < 0.05) in committed subtype.

While gene expression reflects the active state of cell identity, cis-regulatory elements (CREs), including promoters and enhancers underly the determination of cell fate potential^23,24^. Hence, we investigated whether *NPM1*-mutated AML samples would also stratify into primitive and committed clusters based on their cis-regulatory landscape. Using assay for transposase-accessible chromatin sequencing (ATAC-seq) regions of accessible chromatin, typical of CREs, across 18 AML (9 primitive and 9 committed) samples from UHN cohort were identified. CREs are known to form clusters, previously reported as COREs, super-enhancers and stretch-enhancers^25^. Focusing on COREs identified by the CREAM method^26^ resulted in the clustering of the AML samples according to the expression profiles, with one exception (Fig. 3A). Our results indicate that COREs are formed in the promoters in primitive samples as opposed to intergenic regions in committed samples (promoters: FDR = 11%, intergenic: FDR = 2%; Fig. 3B, C and Supplementary Fig. 19). The DNA-binding site motifs for members of RUNX and GATA families, HOXC9 and CTCF were exclusively enriched in the COREs in the primitive subtype, suggesting potential factors in gene regulation in the primitive subtype (Fig. 3D, Supplementary Data 3). Motif-enrichment analysis of exclusively accessible COREs in the committed subtype identified consensus sequences recognized by *CEBP*, ATF family members, *OCT2*, *IRF2*, *NFkB-p65*, *ESRRB*, and *EGR2*, suggesting their potential role in committed subtype (Fig. 3D, Supplementary Data 3).

### Immunophenotyping of primitive and committed subtypes

**Fig. 4:**
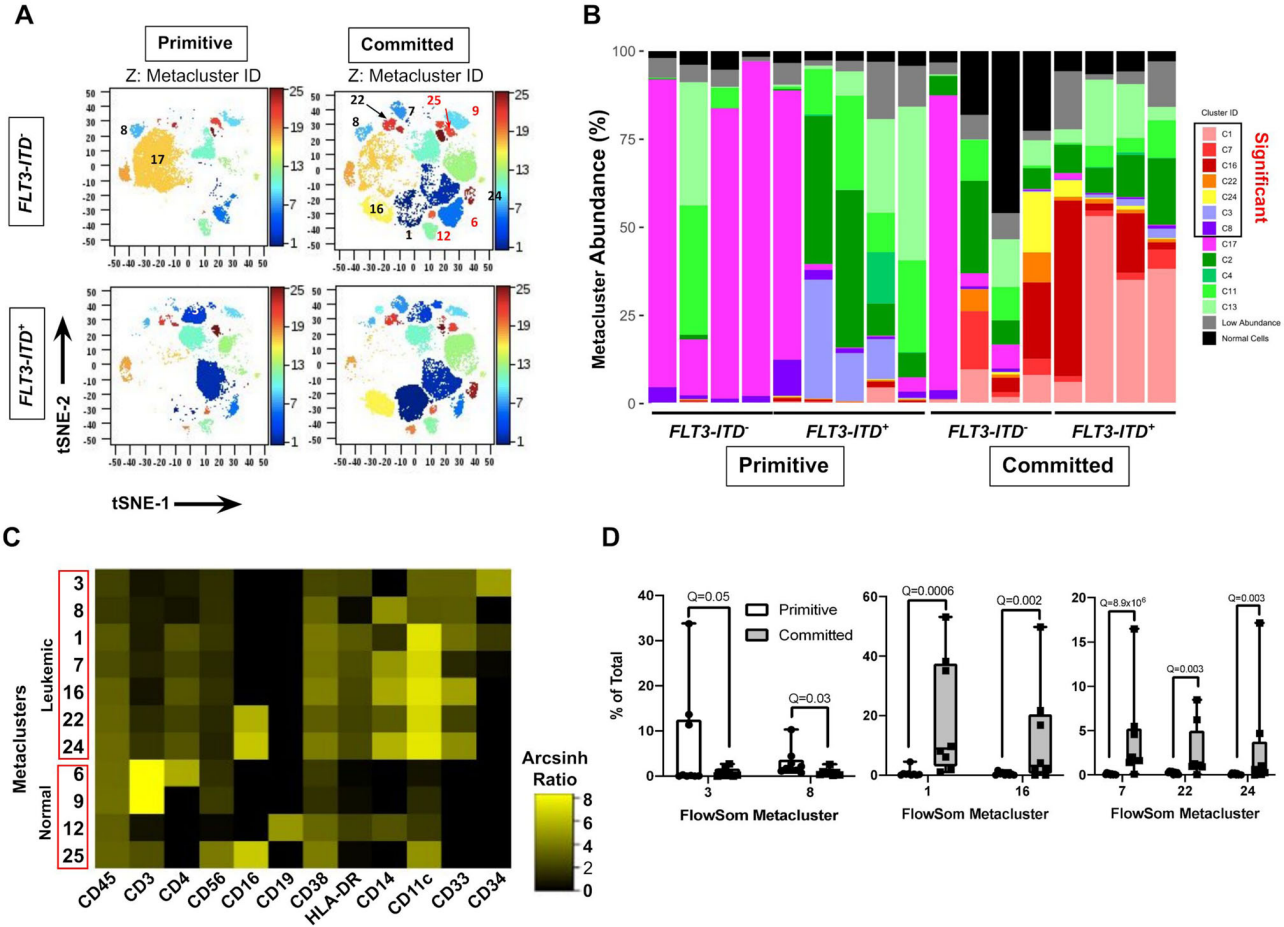
Mass cytometric single cell analysis identifies phenotypic cell clusters that differ significantly between primitive vs. committed *NPM1*-mutated AML cases. **A** tSNE plots of FlowSom immunophenotypic clusters in representative primitive and committed cases with the indicated *FLT3-ITD* genotypes. Twenty-five unique FlowSom immunophenotypic clusters were identified and are colored in the *Z* dimension according to the color scale on the right. The numerical IDs of key leukemic (black numbers) and normal non-leukemic (red numbers) immunophenotypic clusters are labeled. **B** Stacked bar-plot of FlowSom immunophenotypic cluster abundance (% of total) in each sample grouped according to *FLT3-ITD* status within the primitive vs committed groups. immunophenotypic clusters identified by diffcyt as differentially abundant between the two groups (FDR(Q) < 0.05) are boxed as “Significant” in the legend. Leukemic versus normal non-leukemic immunophenotypic clusters were identified based on marker expression (Supplementary Figs. 20, 21). To simplify visualization, very low abundance (<5% of total cells) non-significant leukemic (gray) and all normal non-leukemic immunophenotypic clusters (black) were aggregated. **C** Heatmap representation of the median metal intensity of each marker for each differentially abundant immunophenotypic cluster, represented as the Arcsinh ratio. **D** Box and whisker plots of leukemic immunophenotypic clusters that were differentially abundant between the primitive and committed groups in the diffcyt analysis (*n* = 9 in primitive and *n* = 8 in committed group). The whiskers represent the 1.5 × interquartile range (IQR) extending from the hinges. *Q*-values for each from the diffcyt-DA-edgeR analysis are indicated.

We performed mass spectroscopy-coupled flow cytometer (CyTOF) analysis to explore immunophenotypic differences between 9 primitive and 8 committed AML NPM1-mutated cases at the single-cell level. We used the cytometry (diffcyt)^27^ pipeline to computationally define groups of cells (immunophenotypic clusters) with similar high-dimensional phenotypes. Each immunophenotypic cluster mapped to discrete areas on 2D t-stochastic neighbor embedding (t-SNE) maps (Fig. 4A; Supplementary Figs. 20, 21), confirming that they expressed distinct immunophenotypes. Analysis showed that seven malignant immunophenotypic clusters expressing varying levels of CD45 and markers of hematopoietic progenitors (CD34, CD38) or myelomonocytic differentiation (CD33, CD14, CD11c, CD16, and HLA-DR) typical of AML were differentially abundant between the two subtypes (Fig. 4B, C). Committed cases also contained a higher abundance of non-leukemic immunophenotypic clusters consisting of CD45^hi^ T (CD3^+^), B (CD19^+^) and NK (CD3^−^ CD56^+^ CD16^+^) cells (Supplementary Fig. 20).

Primitive cases of *NPM1*-mutated AML had a significantly higher abundance of immunophenotypic clusters 3 and 8, which were phenotypically primitive, consisting of CD34^+^ CD38^lo^ (3) or CD34^−^ CD38^lo^ (8) cells that expressed low levels of myelomonocytic differentiation markers (Fig. 4C, D, Supplementary Fig. 21). The non-significant leukemic clusters were CD34^−^ but expressed few myelomonocytic markers. In contrast, committed cases of *NPM1*-mutated AML showed a higher abundance of five immunophenotypic clusters (1, 7, 16, 22, and 24) than primitive cases (Fig. 4B, D). These immunophenotypic clusters consisted of CD34^−/lo^ CD38^+^ CD11c^+^ cells that also expressed CD33, CD14, CD16, and HLA-DR in various combinations, revealing aberrant myelomonocytic differentiation (Fig. 4C, Supplementary Fig. 21A). We noted that the total abundance of significant primitive immunophenotypic clusters in primitive cases was lower (mean 9.4 ± 11.4%) than the abundance (mean 53 ± 37%) of the significant differentiated immunophenotypic clusters in the committed cases (Supplementary Fig. 21B). Thus, the primitive cases consistently contained populations of hematopoietic stem/progenitor-like cells together with larger populations that mostly lacked myelomonocytic differentiation and were phenotypically variable within the group. In contrast, committed cases contained a common group of immunophenotypic clusters that compromised larger fractions of the leukemic population and showed advanced myelomonocytic differentiation. Overall these observations demonstrate that leukemic cells from the primitive versus committed cases of *NPM1-*mutated AML exhibit significantly different immunophenotypes at the single-cell level.

Two additional observations suggested that *FLT3-ITD* status also influenced the single-cell phenotypes of *NPM1*-mutated AML. First, although the abundance of immunophenotypic cluster 17, consisting of CD34^−^ CD38^lo^ CD33^lo^ CD14^−^ CD16^−^ CD11c^lo^ CD56^lo^ HLA-DR^lo^ cells, was not significantly different in primitive versus committed cases, it was significantly enriched in primitive versus committed cases specifically within the *FLT3* wild type subset (*P* = 0.05, Fisher’s exact test). Second, immunophenotypic cluster 3 was more abundant in the *FLT3-ITD*-mutated cases overall (Supplementary Fig. 21C), suggesting that this mutation globally promotes accumulation of primitive CD34^+^ CD38^lo^ cells in *NPM1* mutated AML. Nonetheless, among *FLT3-ITD* and *NPM1*-mutated AML, 3/5 of the differentiated immunophenotypic clusters were more abundant in committed versus primitive cases (Supplementary Fig. 21D). Thus, *FLT3-ITD* mutation does not preclude myelomonocytic differentiation in committed *NPM1*-mutated cases. Collectively, these data showed that although *FLT3-ITD* mutation influences the abundance of certain immunophenotypic subsets in *NPM1*-mutated AML cases, the committed subgroup includes more cells with advanced myelomonocytic differentiation independently of *FLT3-ITD* status.

### *NPM1*-mutated AML subtypes are predictive of overall survival

**Fig. 5:**
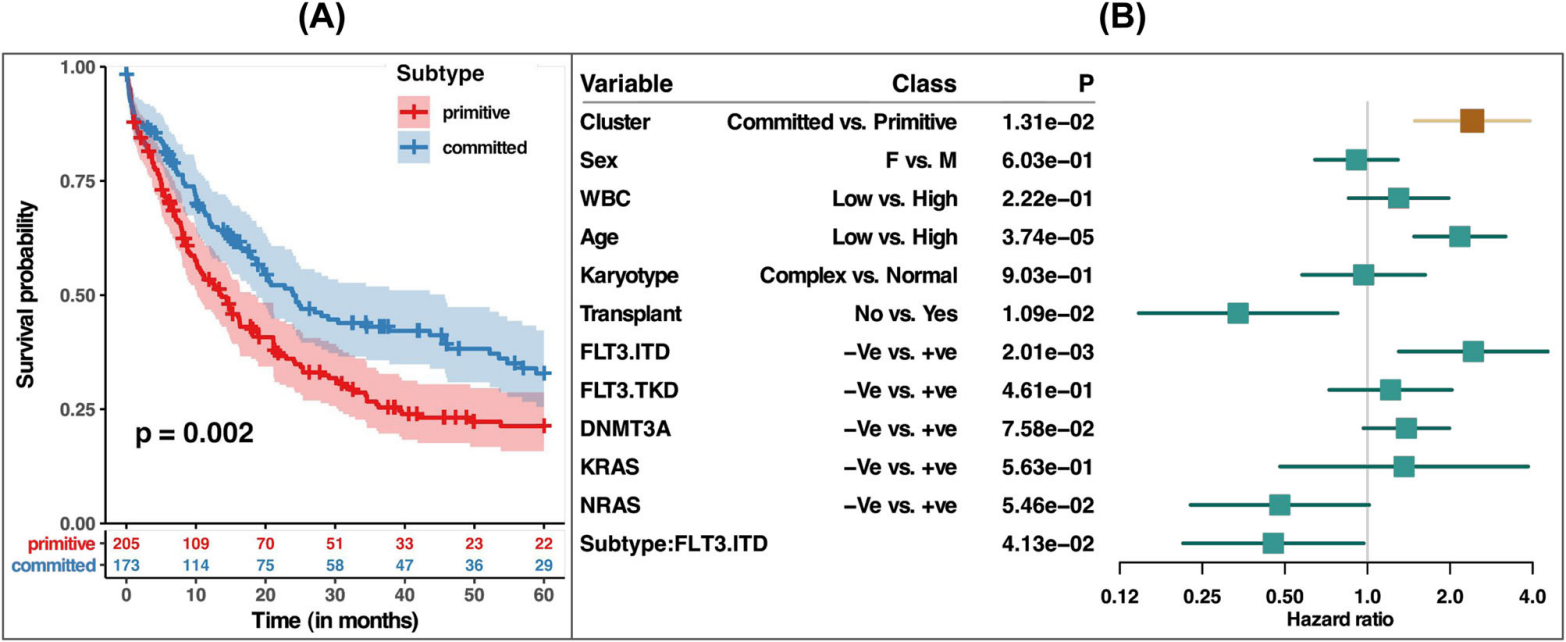
Primitive and committed subtypes are associated with patient survival. **A** Kaplan–Meier estimates of overall survival shows significant differences (log-rank test *p* = 0.002) between primitive and committed subtypes. **B** Forest plot for Cox proportional hazards model-based multivariable analyses of overall survival. In the model, sex, white blood count, age, karyotype status, transplant status, and mutation status of *FLT3*, *DNMT3A KRAS,* and *NRAS* genes are included as covariates.

We next assessed whether the primitive and committed subtypes were associated with patient overall survival (Fig. 5A; Supplementary Fig. 22). We found that the primitive subtype was associated with a significantly worse survival than the committed subtype (Log-rank test *p* = 0.002). To ascertain if our clusters were predictive beyond the established predictive factors, we also fitted a multivariable Cox proportional hazards model, adjusting for clinicopathological parameters, such as sex, white blood cell count, age, karyotype, and mutations, including *FLT3-ITD*, *FLT3-TKD*, *DNMT3A*, *NRAS,* and *KRAS*. This multivariate analysis showed that the primitive and committed subtypes yielded significant complementary prognostic values (*p*-value = 0.01, Fig. 5B).

### The primitive subtype predicts for increased sensitivity to kinase Inhibitors

To identify chemical compounds that may specifically target leukemic cells in the primitive subtype, we used our PharmacoGx platform^28,29^ to mine a large cell line-based pharmacogenomic dataset (CCLE-CTRPv2)^30,31^. This dataset includes over 1000 cancer cell lines treated with up to 544 small molecules along with 68 FDA-approved drugs. Briefly, an ElasticNet model was trained for predicting subtype labels (primitive or committed) using patient data. Using this model, subtype labels for selected cell lines were predicted. Drugs were ranked by the association between their area under the drug dose–response curve (AUC_d_) values and predicted subtype labels across cell lines (detailed methodology for drug ranking is described in Supplementary Discussion and Supplementary Figs. 23–26). We further filtered the drug list by focusing on FDA approved kinase inhibitor drugs as they were ranked high in the list (Supplementary Fig. 23 and Supplementary Data 4 for drug ranking). From the drug ranking list, we selected five kinase inhibitors (Ruxolitinib, Sunitinib, Sorafenib, Quizartinib, Imatinib with FDR < 10%) potentially effective in subtype and Dasatinib as negative control (FDR > 10%) for ex vivo testing.

**Fig. 6:**
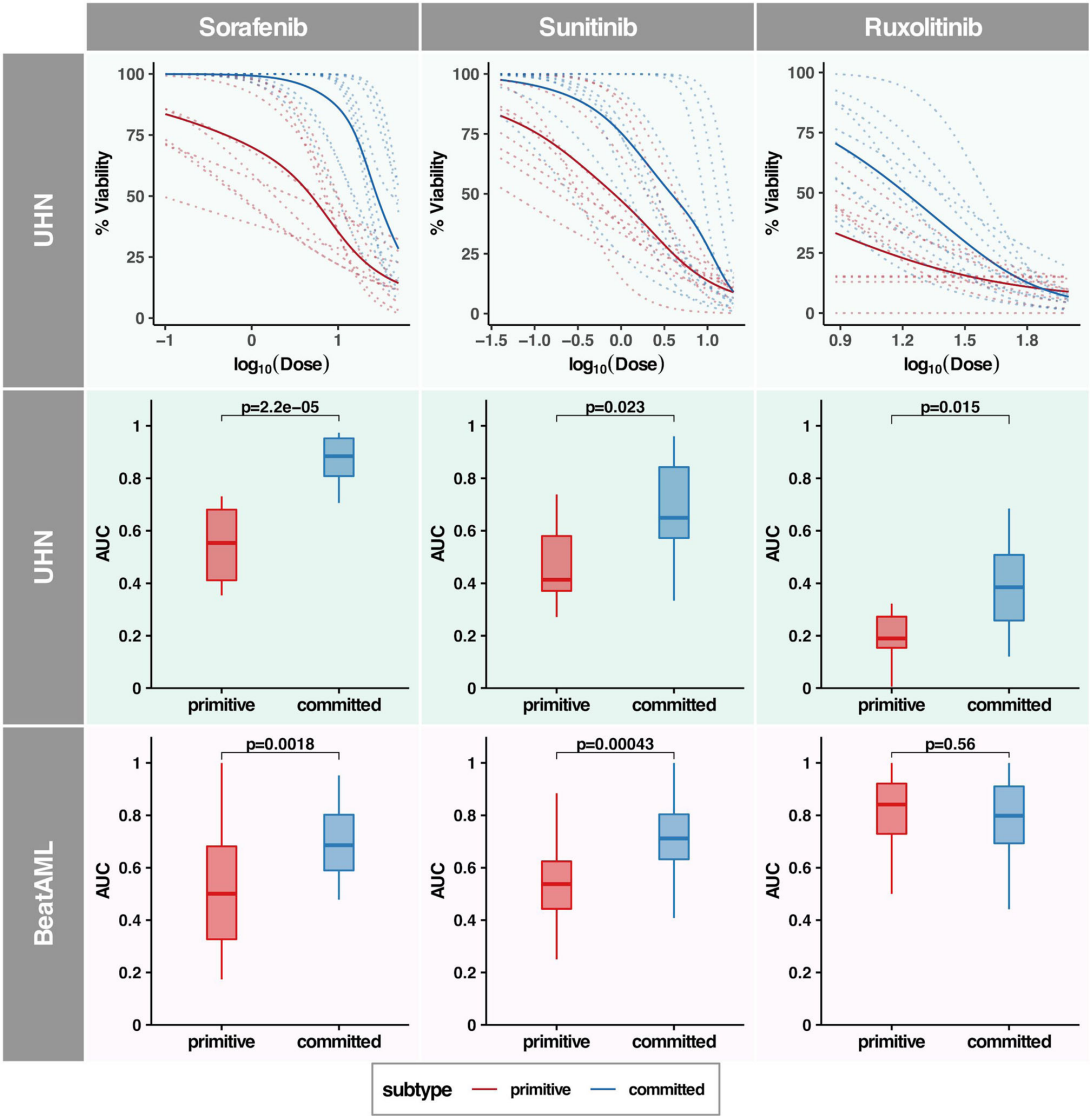
Primitive subtype is more sensitive to kinase inhibitors. Activity of three different kinases inhibitors Sorafenib, Sunitinib, and Ruxolitinib is shown in UHN and BeatAML datasets. Inhibitors were assessed against samples from primitive and committed subtype in patient derived cells (ex vivo drug screening). Top panel shows drug-dose response curves for individual patient samples (in dotted lines) and average across subtypes (in solid line) in the UHN cohort. Lines in red color indicate primitive subtype and blue color indicate committed subtype. The second and third panels show AUC values for ex vivo drug screening in the UHN and BeatAML cohort, respectively. Boxplots in red indicate primitive and in blue indicate committed subtype. Wilcoxon rank-sum test-based *p*-values are indicated on the top of box plots. Samples within the primitive subtype show higher sensitivity (two-sided Wilcoxon rank-sum test *p* < 0.05) against inhibitors Sorafenib, Sunitinib, and Ruxolitinib in the UHN dataset. In the validation cohort (BeatAML), the primitive subtype shows higher sensitivity against Sorafenib and Sunitinib (two-sided Wilcoxon rank-sum test *p* < 0.05).

These six candidate compounds were tested in a subset of the UHN patient samples. For ex vivo screening, patient samples were selected such that *FLT3-ITD* positive and negative samples were represented in each of the two clusters. We investigated whether patient samples with the primitive phenotype have increased sensitivity to kinase inhibitors compared to the committed phenotype. Ex vivo drug screening was performed on 20 patient samples using predetermined dose ranges for all six candidate compounds. Drug dose–response curves were generated and the area under the curve (AUC_d_) was compared between subtypes for each compound (Fig. 6, Supplementary Fig. 27 and Supplementary Data 5 for individual drug dose–response curve). Ex vivo drug screening revealed that patient samples from the primitive cluster were more sensitive to Sorafenib, Sunitinib, and Ruxolitinib compared to the committed subtype (Wilcoxon rank-sum test *p*-value = 2E−5, 0.02, and 0.01, respectively; Fig. 6). Weak differential responses were observed between subtypes for Quizartinib, while Imatinib and Dasatinib showed no differences between subtypes in UHN patient samples (Supplementary Fig. 27).

Next we assessed whether *FLT3-ITD* status and the leukemia stem cell score (LSC17)^32^ predicted drug response independent of the committed/primitive subtypes. Among the six drugs only Dasatinib showed a weak association with *FLT3-ITD* status (Wilcoxon rank-sum test *p*-value = 0.04; Supplementary Fig. 28). No significant association was found between the LSC17 score and drug response (Supplementary Fig. 29). Overall, these results indicate that our subtyping approach can improve stratification of targeted drug sensitivity in *NPM1-*mutated AML patients.

### Validation of subtype association with drug response

We sought to validate the associations between the primitive and committed subtypes with response to kinase inhibitors using the BeatAML^33^ dataset which contains ex vivo drug screening for *NPM1*-mutated AML patient samples. We observed good agreement between the UHN and BeatAML datasets, with samples in the primitive cluster showing a higher sensitivity for Sorafenib and Sunitinib (Wilcoxon rank-sum test *p*-value = 0.002 and 0.0004, respectively; Fig. 6). However, we did not find statistically significant differences in sensitivity to Ruxolitinib in BeatAML cohort (Fig. 6). Interestingly Quizartinib which had a weak differential response in the UHN cohort, showed a statistically significant difference in BeatAML cohort (Wilcoxon rank-sum test *p*-value = 0.001 Supplementary Fig. 27).

## Discussion

Mutations in the *NPM1* gene are the second most common driver genetic abnormalities in AML after lesions in the *FLT3* gene. Due to their distinct clinicopathological and molecular features, *NPM1*-mutated AMLs are considered a separate entity in the genomic classification of AML as well as by the WHO. Despite being well characterized, heterogeneity within *NPM1-*mutated AML has been largely unexplored at the molecular level.

In this study, we characterized the transcriptomic heterogeneity within the *NPM1*-mutated AML patient samples and highlighted the existence of two molecular subtypes across multiple RNA-sequencing datasets. Subsequent analyses revealed that one subtype was enriched for a “primitive” phenotype while the other subtype exhibited a “committed” phenotype. Differential gene expression analysis across all five RNA-sequencing datasets showed that hedgehog-interacting protein (HHIP) gene, an important regulatory component of cell differentiation and hedgehog signaling pathway, was upregulated in the primitive subtype. Immunomodulatory genes such as *CD163* and *CD14* were upregulated in the committed subtype. Furthermore, CyTOF immunophenotypic analysis showed that the committed subtype exhibited more advanced myelomonocytic differentiation. The primitive subtype was also enriched for the presence of the *FLT3-ITD* mutation, while *NRAS*, and *FLT3-TKD* mutations were enriched in the committed subtype. It is important to note that, within the primitive subtype, 36% of the AML samples did not harbor *FLT3-ITD* mutation, yet their transcriptomic profile strongly resembled those of the *FLT3-ITD*-mutated samples. In the committed subtype, 29% of samples showed a gene expression pattern akin to *FLT3* wild type while containing *FLT3-ITD* mutation. Previous studies have shown that within the *NPM1*-mutated patient group, *FLT3-ITD* mutations are associated with a reduced chance of achieving remission with chemotherapy treatment and high risk (70%) of relapse for those achieving remission^9,34^. Conversely, in the absence of a *FLT3-ITD* mutation, patients with *NPM1* mutations are considered to have a favorable outcome with standard induction chemotherapy alone. However, within the *NPM1* mutated and *FLT3* wildtype group, ~40% of patients relapse. The high risk of relapse among these patients cannot be fully explained by the presence of additional genetic mutations. Interestingly, we have found that 37% of *FLT3* wild type samples fall into a primitive subtype and have a signature similar to that of cases with a *FLT3-ITD* mutation. Despite not containing the *FLT3-ITD* mutation, transcriptomic profiles of these samples are very similar to *FLT3-ITD-*mutated samples and exhibited a poor prognosis (Supplementary Fig. 30).

Pathway enrichment analysis revealed that the TLR-signaling pathway is upregulated in the committed subtype; this finding likely reflects the more advanced myelomonocytic differentiation. Comparison of stem and bulk cells with differentiated cells have shown that immune-mediated signaling pathways such as TLR are associated with cell differentiation^35^. Specifically, the increased activity of the TLR signaling pathway has been associated with hematopoietic and AML differentiation^36,37^. Activating TLRs using agonists represents a promising avenue for cancer immunotherapy and there is accumulating evidence supporting a potential role for such agonists in the treatment of AML^38,39^. The differential activity of the TLR pathway among the subtypes might present an opportunity for TLR agonist-based therapy. One possible application would be for patients with the differentiated form of *NPM1* mutant AML who have evidence of residual disease following induction chemotherapy or as maintenance following chemotherapy.

The cis-regulatory landscape of the AML samples strongly concurs with the molecular subtypes defined from gene expression profiles. This is supported by the clustering on the ATAC-seq profiles resulting in 17 out of 18 (94%) of the samples being classified identical to gene expression profiles. The ATAC-seq profiling also reveals that promoter regions are highly enriched in the primitive subtype while intergenic regions are enriched in the committed subtype.

Immunophenotypic profiling showed that in primitive cases two significant immunophenotypic clusters (3 and 8) comprised a much lower fraction of leukemic cells than the non-significant immunophenotypic clusters. In contrast, the significant immunophenotypic clusters in committed cases exhibited clear myelomonocytic differentiation and compromise a much larger fraction of the total. This observation is consistent with the leukemia stem cell paradigm, supported by many studies from our group^40,41^ and independently^42,43^, positing that some myeloid leukemias are initiated by rare stem cell-like cells that retain some capacity to generate more leukemic blasts arrested at a later differentiation stage. These myeloid leukemias retain some aspects of the normal hematopoietic hierarchy, with primitive stem-like cells being much less abundant than their more differentiated progeny. Our finding that the primitive gene expression signature of *NPM1-*mutated AML cases was enriched for stem/progenitor genes supports the idea that the significant immunophenotypic clusters in primitive cases may be enriched for leukemia stem cells. These data highlight that while other genetic lesions can interact with *NPM1* mutations to influence the differentiation status of AML, *FLT3-ITD* status does not override the distinction between primitive and committed cases. The high concordance between RNA- and ATAC-based subtypes together with their distinct immunophenotypic profiles indicate strong biological differences between the primitive and committed subtypes.

To analyze whether subtypes show differential response towards drugs, we conducted ex vivo drug testing using patient samples. The primitive cluster showed higher sensitivity for four different kinase inhibitors. This pattern of differential drug response was also observed in an external dataset, where three drugs (Sorafenib, Sunitinib, and Quizartinib) showed differential response across molecular subtypes. These drugs are potent receptor and intracellular tyrosine kinase inhibitors designed to block tumor cell growth. Sorafenib is known to be effective in samples with *FLT3-ITD* mutations^44^. Furthermore, our results match those observed in earlier studies. Samples with the *FLT3-ITD* mutation show higher sensitivity towards Sorafenib and Sunitinib (Supplementary Fig. 31). However, it is of note that cases within the *FLT3-ITD* mutant group showed a statistically significant difference in drug sensitivity regardless of whether they were from the primitive or committed subgroups (Supplementary Fig. 31, Wilcoxon rank-sum test *p*-value < 0.05 for Sorafenib and Sunitinib).

We discovered that the primitive subtype of *NPM1*-mutated AML is more sensitive to the multikinase inhibitors Sorafenib and Sunitinib compared to committed subtype samples. While the reasons for the increased sensitivity will need further study, gene and pathway level analysis provide potential mechanistic insights that may explain the distinct drug response between the subtypes. The target genes of both Sorafenib and Sunitinib, such as *PDGFRβ* and *KIT*^45–47^ were found to be enriched in the primitive subtype, suggesting the sensitivity of the kinase inhibitors in this subtype. We also observed higher expression of Sorafenib and Sunitinib effector genes such as *CASP3*, *MYCN*, and *MMP2* in the primitive subtype^45,48,49^. Conversely, the committed subtype is enriched in genes and pathways that are implicated in drug resistance. For example, anti-apoptotic *MCL1*, as well as the interleukin signalling pathway and associated markers *IL-6* and *IL-8* are associated with resistance to Sunitinib^50,51^ and their expression is increased in the committed subtype. Thus, while further experiments are required, our data suggest that differences in gene expression between the subtypes may explain the differential sensitivity to kinase inhibitors.

The identification of new molecular subtypes within *NPM1*-mutated AML patients is relevant in the prediction of treatment response and outcome. Currently, most patients with a *NPM1* mutation and without a *FLT3-ITD* mutation, are treated with conventional induction and consolidation chemotherapy. These patients usually do not receive allogeneic hematopoietic stem cell transplant (allo-HSCT) due to their “better” chance of long-term survival and yet a significant proportion of these patients will relapse. Patients with the *FLT3-ITD* mutation, on the other hand receive induction chemotherapy, including *FLT3* inhibitors, followed by stem cell transplant (if other clinical parameters permit), due to their very high relapse rates. The primitive and committed phenotypes identified in this study may provide new parameters for making treatment decisions, as the primitive phenotype contains patients with *FLT3-ITD* negative disease but show a *FLT3-ITD* like transcriptomic profile. As demonstrated in this study, this difference in the transcriptomic profile has predictive implications, with the primitive phenotype showing significantly lower overall survival due to disease recurrence at 5 years compared to the committed phenotype. Given the *FLT3-ITD* phenotype and the survival difference compared to patients without a primitive phenotype, it would be interesting to consider if these patients would benefit from allo-HSCT at first remission, in the same way that is done for patients with *FLT3-ITD*. Furthermore, our study highlights the differential sensitivity of the identified phenotypes to certain kinase inhibitors, which warrants investigation into the potential benefit of including these kinase inhibitors in the *NPM1* treatment regimen or as maintenance therapy, for patients with the identified phenotype.

In conclusion, the present study provides evidence that the *NPM1-*mutated AML can be stratified into primitive and committed subtypes that have different 5-year survival and drug sensitivity to agents targeting signaling pathways. The transcriptomic, ATAC-seq, and immunophenotypic profiles identified in this study have implications in predicting response to therapy and potential changes in treatment decisions. This includes the consideration of stem cell transplant after first remission for the primitive subset of *NPM1-*mutated AML cases whose tumor cells lack a *FLT3-ITD* mutation. We also contemplate the possibility of adding kinase inhibitors to the current *NPM1-*mutated AML treatment regimen for selected cases with a primitive pattern of gene expression or TLR-targeted immunotherapy.

## Methods

### AML cohorts

In this study, we used five different AML patient cohorts. The UHN cohort consists of 77 *NPM1*-mutated AML patients and was used as a discovery cohort (data available at European Genome-Phenome Archive accession id EGAD00001006669). A written informed consent was obtained in accordance with the Declaration of Helsinki and University Health Network (UHN) institutional review board. The studies protocol was approved by the ethics board of University Health Network, Toronto Canada. For validation of clustering and pathway analysis results, the TCGA-LAML^52^, Karolinska Institutet (KI)^53^, BeatAML^33^ and Leucegene^54^ patient cohorts were used. The TCGA, KI, BeatAML, and Leucegene cohorts consist of 48, 79, and 77 and 97 *NPM1*-mutated AML patients, respectively. We performed transcriptomic RNA sequencing on the UHN cohort (details below). For the TCGA cohort, raw RNAseq and mutational data was retrieved from the data portal of TCGA (https://portal.gdc.cancer.gov/projects/TCGA-LAML). Transcriptomic RNA sequencing, somatic mutation panel, and bioinformatics analysis of the KI AML cohort was performed at Karolinska Institutet, Sweden (data available at 10.5281/zenodo.292986. Raw transcriptomic profiling and somatic mutation data for the BeatAML^33^ cohort was obtained from 10.1038/s41586-018-0623-z. Leucegene^54^ cohort data was obtained from the NCBI-GEO ids GSE49642, GSE52656, GSE62190, GSE66917, GSE67039.

### RNA extraction and preparation

For the UHN cohort, fresh bone marrow or peripheral blood cells were collected from AML patients at diagnosis according to the protocol approved by the University Health Network institutional review board. RNA was extracted from 77 *NPM1=*mutated samples. As a normal control, RNA was obtained from discarded mobilized peripheral blood CD34+ mononuclear cells. These samples were obtained following separate REB approval. RNA from OCI-AML2 (*NPM1* wild type) and OCI-AML3 (*NPM1* mutant) cell lines was also obtained for comparison. RNA was extracted from (1 × 10^7^) cells using the RNeasy Plus Mini Kit (74134, Qiagen Sciences), and was quantified using the Quant-iT RNA Assay Kit, Broad Range (Q10213, Invitrogen). Library preparation was prioritized based on RNA quality, as judged by RNA electrophoresis on a 1% agarose gel. The gel was run at 60 V for 1.5 h or until the dye was half way down the gel. Quality of RNA was analyzed based on the presence of two rRNA bands and an mRNA smear on the gel.

### Library preparation and sequencing and data processing

Libraries were prepared for Illumina Sequencing using the Illumina TruSeq RNA Sample Preparation Kit v2 (RA-122-2001, Illumina). Up to eight samples were processed at a time in a 96-well plate. DNA libraries were quantified using the Quant-iT dsDNA Assay Kit, Broad Range (Q33130, Invitrogen), with fluorescence values measured at excitation/emission maxima of 485/538 nm. Library quality check was performed by Bioanalyzer at The Centre for Applied Genomics (TCAG) at Sickkids Research Institute using the DNA1000 kit (Agilent Technologies). The following equation was used to calculate nanomolar (nM) concentrations so that libraries could be diluted to 4 nM:

1 $${\mathrm{nM}}\,{\mathrm{concentration}} = \frac{10^6 \times ({\mathrm{{ng}}/{\mathrm{{\mu}}}{\mathrm{l}}\, {\mathrm{{concentration}}}})}{ (660\,\times\,{\mathrm{average}}\,{\mathrm{fragment}}\,{\mathrm{length}}\,{\mathrm{in}}\,{\mathrm{basepairs}})}$$

Samples were pooled such that 2 μL of the 4 nM dilution was combined for each of 12 libraries with unique barcoding indexes. Seven pools of 12 libraries were sent to the University of British Columbia (UBC) Sequencing Centre, where they were re-quantified using the Qubit dsDNA BR Assay Kit (Q32850, Invitrogen). This reading was used to further dilute pools, accounting for any discrepancies in quantification methods. Sequencing was performed at UBC Sequencing Centre in paired-end reads of 100 bp on the Illumina HiSeq 2500. Primary data analysis was performed at the Centre for Computational Medicine (CCM) at SickKids Research Institute.

Filtered reads were aligned to Ensembl human reference genome GRCh37. Raw reads were counted using HTSeq count version 0.6.1^55^. Expression levels from RNAseq data were obtained using Kallisto version 0.45.0^56^. Features which have a read count equal to zero in more than 10% of samples were removed.

### Clustering analysis

Detailed description of method employed for clustering and subtype annotation is provided in Supplementary Methods. A consensus clustering-based unsupervised learning approach was applied to all five gene expression datasets independently^57^. The consensus clustering approach performs repeated clustering on the randomly selected part of the data and aggregates the results to discover robust clusters. At each round of clustering 80% of samples and 80% of the features were selected and the K-medoids clustering algorithm was applied on the selected data, where the value of *k* (number of clusters) varies from two to eight clusters. In the first step, the K-medoids clustering algorithm randomly selects *k* data-points and uses them as medoids of the clusters. The algorithm assigns each sample to a cluster such that the distance from the medoid to the sample is minimal. In the next step, the algorithm recalculates medoids for newly formed clusters and reassigns the samples to the clusters based on the distance. The algorithm repeats these steps until it converges. We repeated this clustering procedure 100 times and created consensus matrices by aggregation of the results. Final clustering was performed on the consensus matrices. The optimal number of clusters was discovered using silhouette distance which is a measure of how close each point in a cluster is to points in the neighboring clusters. Thus a higher silhouette value for a sample indicates that the sample is very near to the cluster it is assigned to and far away from its neighboring cluster. We applied the same clustering approach to all four datasets independently.

Finding robust clusters across several datasets is challenging as first batch correction techniques need to be applied to remove dataset-specific noise. However, such transformation of data can potentially smooth out true signaling patterns. To overcome this challenge, we applied the CoINcIDE framework^12^ which requires no between-dataset transformations. Using the CoINcIDE approach, we computed similarities between clusters from different datasets. First centroids of every cluster within a dataset are derived. Then these centroids were compared to the centroid of the cluster from other datasets. For distance measure between cluster centroids the Pearson correlation coefficient was used. The distance between clusters originating from different datasets was represented as a network. Labels propagation-based community detection algorithm was applied on this network to detect the groups that consist of densely connected nodes. Network analysis was done using igraph R package (version 1.2.4.2).

### Differential gene expression and pathway analysis

Differentially expressed genes between the clusters were determined using the DESeq2 package (version 1.18.1) in R/Bioconductor^58^ in each cohort independently. For each gene, a meta estimate was obtained by combining log fold change values from all four cohorts. For meta estimate calculation we used the combine.est function in the survcomp package (version 1.28.5)^59^. Using the meta estimate value, pathway enrichment analysis was performed. Overrepresentation of pathways was tested using the hypergeometric model. The pathways were defined using the Reactome pathway database. Analysis was performed using the R Piano package (version 1.20.1). Cellular composition deconvolution was performed using PERT deconvolution method^13^ and batch-corrected linear RMA-normalized data from the GSE24759 (DMAP) were used as the reference profile. The vector theta from the PERT output was used to estimate the percentage of reference populations.

### ATAC-seq and CORE analysis

A subset of patient samples (*n* = 20) was analyzed using ATAC-seq (data available at European Genome-Phenome accession id EGAD00001006670). Library preparation was performed on 30,000 blast cells (CD45+CD33+CD3−CD19−), sorted from patient samples, using the Nextera DNA samples Preparation kit (FC-121-1030, Illumina) according to previously reported protocol^60^. Libraries were sequenced with HiSeq 2500 System (Illumina) to generate single-end 50 bp reads. Raw single-end reads were aligned to hg19 using BWA (version 0.7.17) with default parameters. Any reads mapping to the mitochondrial chromosome, or to a set of hg19 blacklisted regions were removed. Any reads with a quality score *Q* < 30 were also removed and duplicate reads were marked. MACS2 was used to identify peaks of enriched chromatin accessibility at a *q*-value ≼ 0.05, using default parameters plus -SPMR, -nomodel, and The CREAM R package (version 1.1.1)^26^ was used to call clusters of cis-regulatory elements (COREs) using the ATAC-seq profiles of the AML samples. Overlap of the COREs between the AML samples were used to identify the jaccard index for similarity of each pair of samples. We then used the jaccard index to cluster the samples. Function assignChromosomeRegion from R library ChIPpeakAnno was used in combination with R library TxDb.Hsapiens.UCSC.hg19.knownGene to determine the genomic distribution of COREs. The categories considered were Promoters, immediate Downstream, 5′UTRs, 3′UTRs, Exons, and Introns. Genes within 10 kb proximity of COREs are considered as the genes associated with COREs.

We identified 1569 COREs found only in primitive subtypes, and shared across at least two primitive samples. 2578 COREs were also determined only in committed subtype, and shared across at least two committed samples. Then, using *findMotifsGenome.pl* from HOMER v4.7^61^, we detected transcription factor-binding site (TFBS) motifs enriched in COREs exclusively accessible in primitive and committed subtypes. Regions exclusively accessible in each subtype were used as the background set when identifying enrichments of TFBS motifs in the other subtype. The top 10 TFs were selected if DNA recognition site motifs were enriched in each subtype at FDR <5%. We filtered out the motifs if they were present ≤2% of the target sequences. Fold enrichment of the TFBS motif was calculated by taking the log2 ratio of percentage of the target sequences with motif to the percentage of the background sequences with motif.

### Computational drug prioritization

Comprehensive method for drug prioritization is described in Supplementary Discussion and Supplementary Figs. S23–S26. First, using the gene expression data from the UHN dataset as features, we trained Elastic Net-based supervised machine learning models. The labels from consensus clustering-based unsupervised learning were used as the target. On the UHN dataset, ten fold cross validation was performed and Elastic Net parameters (alpha and lambda) were optimized. To divide the data into training and test sets, we applied a random but class balanced split approach. Other patient datasets, TCGA, KI, Leucegene, and BeatAML were used as independent external test sets. Performance of the models was assessed in terms of accuracy to predict subtype labels. The best performing model was used to classify each cell-line into the CCLE-CTRPv2 dataset. Along with the class label, the Elastic Net model also provides class probability. Using the R PharmacoGx package (version 3.8)^28^, we computed the concordance index (CI) between the predicted cell-line labels and area under the drug dose–response curve (AUC), for each drug. The value of CI is used to rank and prioritize compounds for ex vivo testing (Supplementary Fig. 26).

### Drug sensitivity testing and validation

A subset of patient samples (*n* = 20) from the UHN cohort were selected for drug sensitivity testing, using parameters of clustering robustness and *FLT3-ITD* status. Kinase inhibitors were chosen to cover a range of targets, including *FLT3*, and were obtained as powder from SelleckChem or Sigma-Aldrich, from which stock solutions were prepared in dimethyl sulfoxide (DMSO). Patient samples were prepared in a 1:10 ratio of sample to thawing solution (5 mL XVIVO10 Media, 5 ML fetal calf serum (FCS), and 200 μL of (1 mg/mL) DNase). Pelleted patient cells were resuspended in Iscove’s modified Dulbecco’s medium (IMDM) and transferred to Long-Term Culture Media (Myelocult H5100 media supplemented with 1% penicillin/streptomycin, 10–6 M hydrocortisone sodium succinate, and the following 7 cytokines: SCF, IL-7, IL-6, IL-3, FLT3-L, G-CSF, and CM-CSF) for plating. Optimal dose concentration ranges were selected such that at least 80% viability was achieved at the lowest dose and ~0% viability was achieved at the highest dose when tested in *NPM1* wild type OCI-AML2 cells. Ten dose concentrations of Sorafenib, Ruxolitinib, Sunitinib, Quizartinib, Imatinib, and Dasatinib were tested, in triplicate, in each of the selected patient samples, and cell viability was measured with the Cell-Titer Fluor Assay (G6082, Promega) after 72 h incubation. The drug dose response data was processed in the R PharmacoGx package (version 3.8)^28^. For the validation of drug response, BeatAML data was obtained from the publication^33^. Raw drug dose-response curves were processed using a similar pipeline as to UHN drug-response data. To compute AUC, all dose–response curves were fitted to the following Hill equation:

2 $$y = 1/(1 + \left( {x/{\mathrm{{EC}}}_{50}} \right)^H)$$

where EC_50_ is the half-maximal effective concentration and *H* is the Hill coefficient. Drug AUC values were computed using the computeAUC function of the PharmacoGx package (version 3.8)^28^.

### Cell staining for mass cytometry analysis

Cryopreserved diagnostic AML patient samples were thawed and washed twice in pre-warmed complete (c) RPMI (RPMI, 10% FBS, 25 mM Hepes, 55 μM β-mercaptoethanol, 0.1 mM non-essential amino acids, 1 mM sodium pyruvate, 2 mM l-glutamine) containing Benzonase (Millipore Sigma, Catalog # E1014) and MgCl_2_. Cells (2 × 10^6^ cells/ml) were rested in cRPMI in a humidified 5% CO_2_ incubator for 85 min at 37 °C, prior to distributing to cluster tubes. Cells were then stained with 3 μM Cisplatin (BioVision Inc., USA) for 5 min prior to fixing with 1.6% ultra-pure formaldehyde (Analychem Corp. Catalog # 18814-20) for 10 min at room temperature (RT). Cells were then washed thrice with a staining buffer (PBS + 1% BSA) prior to barcoding with the Cell-ID 20-Plex Pd Barcoding Kit (Fluidigm, Catalog: 201060) according to the manufacturer’s instructions. Six to seven bar-coded samples were then combined into single 15 ml polypropylene tubes for multiplexed staining with a panel of 12 metal-tagged antibodies specific for cell surface markers (Supplementary Table 4) as previously described^62^. After a final wash, cells were resuspended in PBS containing 0.3% saponin, 1.6% formaldehyde, and 100 nM ^191/193^Iridium (Fluidigm, Catalog #201192B) to stain nuclear DNA for up to 48 h at 4 °C. Prior to analyzing stained cells on the Helios, they were washed and re-suspended in Maxpar Cell Acquisition Solution (Fluidigm, Markham ON, Canada) at 2–5 × 10^5^/ml followed by addition of five-element EQ normalization beads (Fluidigm, Markham ON, Canada) according to the manufacturer’s instructions. Samples were acquired on a Helios with a wide-bore injector according to Fluidigm’s protocols. CD45-89Y was purchased conjugated from Fluidigm. The remaining antibodies were purchased and metal-tagged in-house (CJG lab) using Fluidigm Maxpar Metal Conjugation Kits according to the manufacturer’s instructions. Details of the antibody panel can be found in Supplementary Data File 7.

### CyTOF data analysis

Raw CyToF FCS data and protocol information are available at FlowRepository accession ID: FR-FCM-Z36E. The Helios software (v6.7.1014) was used to generate and normalize FCS 3.0 datafiles which were then uploaded into Cytobank (Santa Clara, CA). After Arcsinh transformation of each parameter (scale argument = 5), FCS files were de-barcoded using an open-source algorithm^63^. Two samples were omitted from further analysis due to high cell death. The de-barocded FCS 3.0 files were then re-uploaded to Cytobank to gate out doublets and dead cells. FCS 3.0 files containing 42,054 live single cells were exported and the diffcyt package (version 1.8.6)^27^ in R version 3.1.1 was used to assess differences in single cells between the primitive and committed groups. Within the diffcyt workflow we first used FlowSom^64^ (*K* = 25) and the cell type markers CD45, CD19, CD3, CD4, CD11c, HLA-DR, CD33, CD56, CD34, CD16, CD38, and CD14 (Arcsinh transformation of each parameter (scale argument = 5)) to define immunophenotypic clusters of cells with similar high-dimensional phenotypes. We used FlowFrame from the ‘flowCore’ Bioconductor R package (version 2.0.1) to create new FCS files that include the FlowSOM immunophenotypic cluster ID for each cell. These modified FCS 3.0 files were then uploaded to Cytobank to perform t-SNE dimensionality reduction using the cell type markers and FlowSom immunophenotypic cluster IDs with the following run parameters: iterations: 4000, perplexity: 30, theta: 0.5. Finally, we used diffcyt-DA-edgeR^27^ to identify differentially abundant immunophenotypic clusters (false discovery rate (FDR) < 0.05). Out of 13 differentially abundant immunophenotypic clusters, we excluded #15 from further analysis since it represented <0.5% of total cells in all samples. Prism v8.2.0 was used to generate boxplots and compute statistics comparing FlowSOM immunophenotypic cluster abundance.

The stacked barplot visualization of FlowSom immunophenotypic cluster abundance was generated using the ggplot2 package in R version 3.1.1. Median metal intensity by each immunophenotypic cluster was calculated using the diffcyt R package. Arcsinh ratios were calculated in R and the heatmap.2 function from the gplots package was used to generate the heatmap. The Arcsinh ratio represents the Arcsinh (scale argument = 5) transformed ratio of the median marker intensity in each cluster divided by the median marker intensity of the sample with the lowest expression in the group.

### Reporting summary

Further information on research design is available in the Nature Research Reporting Summary linked to this article.

## Supplementary information

Supplementary Information

Description of Additional Supplementary Files

Supplementary Data Files 1–6

Reporting Summary

## Data Availability

The raw RNA-seq and ATAC-seq data generated from the UHN AML patient cohort have been deposited at the European Genome-Phenome Archive under the accession id EGAD00001006669 for RNAseq and EGAD00001006670 for ATACseq data. Access to these data can be obtained through applying to the Data Access Committee for the dataset. Information on how to apply for access is available at each EGA dataset link. CyToF FCS files, other related data and protocol information are available at FlowRepository ID: FR-FCM-Z36E. Published datasets used in this manuscript are available through the following websites or accession numbers: (1) TCGA-LAML; (2) BeatAML 10.1038/s41586-018-0623-z; (3) KI 10.5281/zenodo.292986; (4) Leucegene NCBI-GEO accession ids GSE49642, GSE52656, GSE62190, GSE66917, GSE67039. Source data are provided with this paper and as a docker image at 10.24433/CO.3416958.v1. The authors declare that all other data supporting the findings of this study are available within the article and its Supplementary Information files, or are available from the authors upon request. Source data are provided with this paper.

## Source data

Source Data

## Code availability

A complete software environment through CodeOcean containing all necessary data and code to reproduce the analysis and figures described in this manuscript is available at 10.24433/CO.3416958.v1. All the codes and data are also available via github at https://github.com/bhklab/NPM1-AML.
