# Supplementary material for: Biological and therapeutic implications of a unique subtype of NPM1 mutated AML
Source: Nat Commun. 2021 Feb 16;12:1054. doi: 10.1038/s41467-021-21233-0 (PMC7886883; doi:10.1038/s41467-021-21233-0)
Supplement: Supplementary file 1 — Supplementary Information [file 41467_2021_21233_MOESM1_ESM.pdf]

Supplementary Information

Biological and Therapeutic Implications of a Unique Subtype of NPM1 Mutated AML

Arvind Singh Mer[1,2,3], Emily M Heath[1], Seyed Ali Madani Tonekaboni[1,2], Nergiz Dogan-Artun[1], Sisira Kadambat Nair[1], Alex Murison[1], Laura Garcia-Prat[1], Liran Shlush[4], Rose Hurren[1], Veronique Voisin[5], Gary D. Bader [5,6], Corey Nislow[7], Mattias Rantalainen[3], Soren Lehmann[3], Mark Gower[8], Cynthia J. Guidos[8], Mathieu Lupien[1,2,9], John Dick[1], Mark D. Minden[**1,$**], Aaron D. Schimmer[**1,$**], Benjamin Haibe-Kains[1,2,6,9,10,$]

[1] *Princess Margaret Cancer Centre, University Health Network, Toronto, Ontario, Canada*

[2] *Department of Medical Biophysics, University of Toronto, Toronto, Ontario, Canada*

[3] *Karolinska Institute, Stockholm, Sweden*

[4] *Department of Immunology, Weizmann Institute of Science, Israel*

[5] *The Donnelly Centre, University of Toronto, Toronto, Ontario, Canada*

[6] *Department of Computer Science, University of Toronto, Toronto, Ontario, Canada*

[7] *Faculty of Pharmaceutical Sciences, The University of British Columbia, Canada*

[8] *The Hospital for Sick Children, Toronto, Ontario, Canada*

[9] *Ontario Institute for Cancer Research, Toronto, Ontario, Canada*

[10] *Vector Institute, Toronto, Ontario, Canada*

[$] **<u>Joint corresponding authors</u>**

Aaron Schimmer

Princess Margaret Cancer Centre, University Health Network, Toronto, Ontario M5G 1L7 Canada

Phone: +1 416-946-4568 E-mail: Aaron.Schimmer@uhn.ca

Benjamin Haibe-Kains,

Princess Margaret Cancer Centre, University Health Network, Toronto, Ontario M5G 1L7 Canada

Phone: +1 (416) 581-7628 E-mail: bhaibeka@uhnresearch.ca

Contents

1 Supplementary Methods 6

1.1 Patient cohorts 6

1.2 Clustering of individual cohorts 6

1.3 Meta clustering 9

1.4 Subtype annotation 10

2 Supplementary Discussion 11

2.1 Subtype and Mutations 11

2.2 Subtype and Mutation Gene Signatures 17

2.3 Subtype and Mutations Pathway Analysis 26

2.4 Drug Prioritization 34

List of Figures

1 Clustering and subtype discovery pipeline 8
2 Silhouette width for clusters in all datasets 9
3 Correlation between mutations and subtypes 12
4 Mutation frequency vs. subtypes 13
5 Allele burden of FLT3-ITD 14
6 Allele burden of DNMT3A 15
7 ROC curve for multivariate mutation analysis 16
8 Volcano plot for subtypes 18
9 Volcano plot for FLT3-ITD mutation 19
10 Volcano plot for DNMT3A mutation 20
11 Expression of C1QA 21
12 Expression of MARCO 22
13 Expression of ZNF521 23
14 Expression of CDH2 24
15 Venn diagrams for subtype versus mutation groups 25
16 Pathway enrichment analysis 27
17 Upset plot of differentially expressed genes 28

18 Network for pathway enrichment 29
19 Comparison of COREs 30
20 CyTOF analysis of AML samples 31
21 Meta clusters from CyTOF analysis 32
22 KM plot for datasets 33
23 Pharmacogenomic pipeline for drug ranking 34
24 Cell lines to patient correlation 35
25 Subtype model accureacy 37
26 Drug prioritization pipeline 38
27 *Ex vivo* drug response 39
28 FLT3-ITD vs. drug response 40
29 LSC vs. drug response 41
30 KM plot for subtype and FLT3-ITD 42
31 Drug response by subtype and FLT3-ITD 43
32 Cross validation of meta clustring 44

List of Tables

1 Description of AML datasets used in the study . . . 6
2 Subtypes vs. Age . . . 45
3 Subtypes vs. WBC . . . 45
4 Subtypes vs. karyotype . . . 45
5 Subtypes vs. Sex . . . 46
6 Subtypes vs. transplant . . . 46
7 Subtypes vs. FLT3-ITD . . . 46
8 Subtypes vs. NRAS . . . 47
9 Subtypes vs. DNMT3A . . . 47
10 Subtypes vs. KRAS . . . 47
11 Subtypes vs. FLT3-TKD . . . 48
12 Subtypes vs. PTPN11 . . . 48
13 Subtypes vs. WT1 . . . 48
14 Subtypes vs. IDH2 . . . 49
15 Subtypes vs. CEBPA . . . 49
16 Subtypes vs. ASXL1 . . . 49
17 Subtypes vs. RUNX1 . . . 49

1. Supplementary Methods

1.1 Patient cohorts

This study describes two novel subtypes of NPM1 mutated AML patients. These subtypes are called primitive and committed, respectively. For the discovery and validation of these novel subtypes we have used five different AML patient cohorts. Details about these patient cohorts are available in table 1.

Cohort Name	# Samples	Source
UHN	77	Internal
TCGA	48	[1]
KI	79	[2]
Beat-AML	77	[3]
Leucegene	97	[4]

Supplementary Table 1: Description of AML datasets used in the study

1.2 Clustering of individual cohorts

Clustering and subtype discovery pipeline is shown in figure 1. Unsupervised machine learning (clustering) was performed on each patient cohort separately. To find robust clusters we applied a consensus clustering approach [5]. Using the consensus clustering approach, clustering is performed on the randomly selected part of the data. This is done repeatedly to ensure robust clustering. In the final step results from all clustering rounds are aggregated to create a final consensus matrix. This process is done assuming

the possible number of clusters in the dataset ranging from 2 to 8. To find the optimal number of clusters we used silhouette width which measures how well data points are clustered. A higher value of silhouette width indicates a better quality of clustering. Figure 2 shows silhouette width in each dataset at different numbers of clusters. Here we observe that for all datasets, at $K = 2$ (number of clusters) the silhouette width is maximum. Therefor two clusters is the optimal partition for all five datasets.

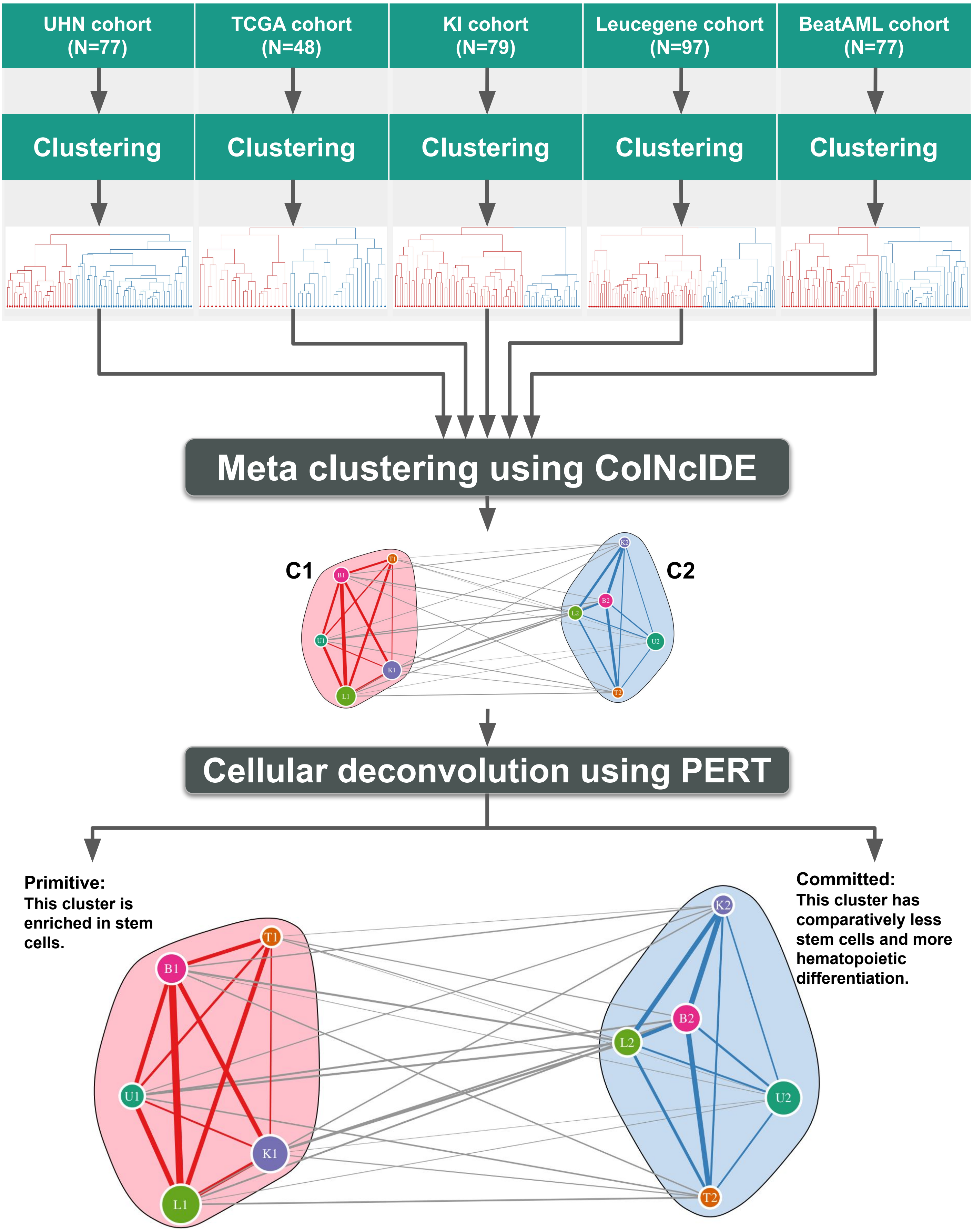

Supplementary Figure 1: Clustering and subtype discovery pipeline. Clustering was performed on each dataset separately. Next clusters from different cohorts were merged together using the CoINcIDE framework. This provided two robust clusters. Samples in both clusters were analyzed using the PERT cellular deconvolution method. PERT analysis showed that one cluster is enriched in stem cells, hence termed as primitive and the other cluster is termed as committed.

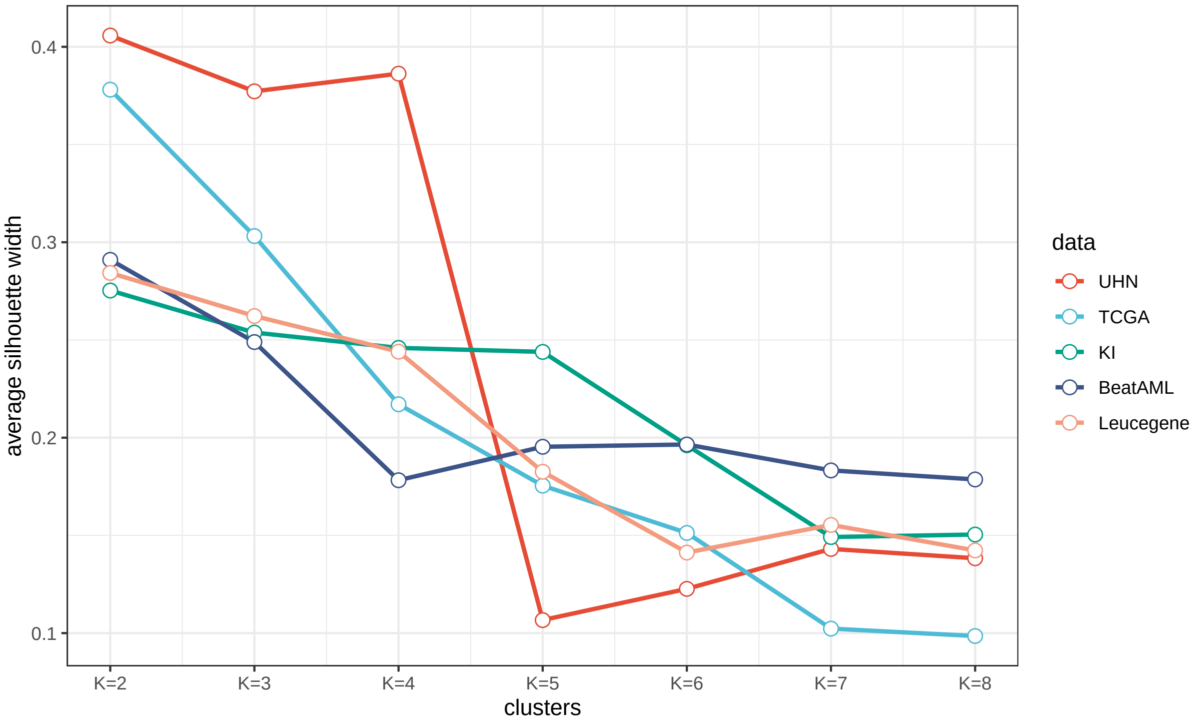

Supplementary Figure 2: Silhouette width for clusters in all datasets. The silhouette width estimates the mean distance between clusters. The average silhouette width for different numbers of clusters (K) in four datasets. A higher silhouette width indicates a better quality of clustering. At K=2 silhouette width is highest for all five datasets.

1.3 Meta clustering

From the previous clustering step, we obtained two clusters in each dataset. This is indicated as red and blue color branches of the dendrogram in figure 1. Although using the silhouette width we have established that there are two clusters in each dataset (figure 2), the question remains which cluster from a dataset is more similar to a cluster from the other dataset. For example, whether the red cluster from the UHN dataset is more analogous to the red or blue cluster from the TCGA dataset. To resolve this, we mapped clusters from each cohort into clusters from other cohorts. This is done using the CoINcIDE framework [6]. This approach allowed us to find clusters across datasets

without the need of any batch correction technique or between-dataset transformations. From the CoINcIDE based meta clustering we deduce two clusters, indicated as $C1$ and $C2$ in figure 1.

1.4 Subtype annotation

After establishing two distinct clusters from meta-clustering (called $C1$ and $C2$), we further characterized these clusters. For this purpose we used the PERT cellular deconvolution algorithm [7]. The PERT algorithm is specifically designed for deconvolution of human hematopoietic samples. It works by comparing a given sample's gene expression profile to several well established reference cell population. Deconvolution through this algorithm indicates that one meta-cluster is enriched in stem cells. The enrichment of stem cells indicates that this cluster is still in the primitive state of haematopoietic stemness [8, 9]. Therefore this meta-cluster is named *primitive*. Our analysis also indicates that the other cluster has less stem cells and more haematopoietic differentiation. This implies a rigid commitment to haematopoietic differentiation hierarchy, accordingly this cluster is named *committed*.

2. Supplementary Discussion

2.1 Subtype and Mutations

We analyzed the association between subtypes and mutation status of key genes. Oncoprint in the main Figure 2C (in manuscript) shows the mutation status of genes along with predicted primitive or committed subtypes of samples. Matthews correlation coefficient (MCC) analysis between subtypes and mutation shows a weak association (figure 3). For FLT3-ITD and DNMT3A, a MCC value of 0.32 and -0.16 was observed respectively. However Chi-square test based FDR for FLT3-ITD, DNMT3A, NRAS and KRAS is statistically significant ($FDR < 0.05$, for details see Supplementary Table S6-S17). Together these analyses indicate that there is a weak but statistically significant association between subtypes and few key genes. This prompted us to ask the question if subtypes are solely driven by mutations.

Subtype and Mutation Frequency

First we investigated whether samples belonging to one of the subtypes have higher mutation frequency (figure 4). We found that primitive and committed subtypes are not different in terms of mutation frequency (two-sided Wilcoxon rank-sum test $p - value = 0.9$).

Subtype and Mutation Allele Burden

Next we analyzed if allele burden is associated with subtypes (figure 5 and 6). Allele burden was defined as the ratio of mutant versus wild type reads of the gene [10, 11].

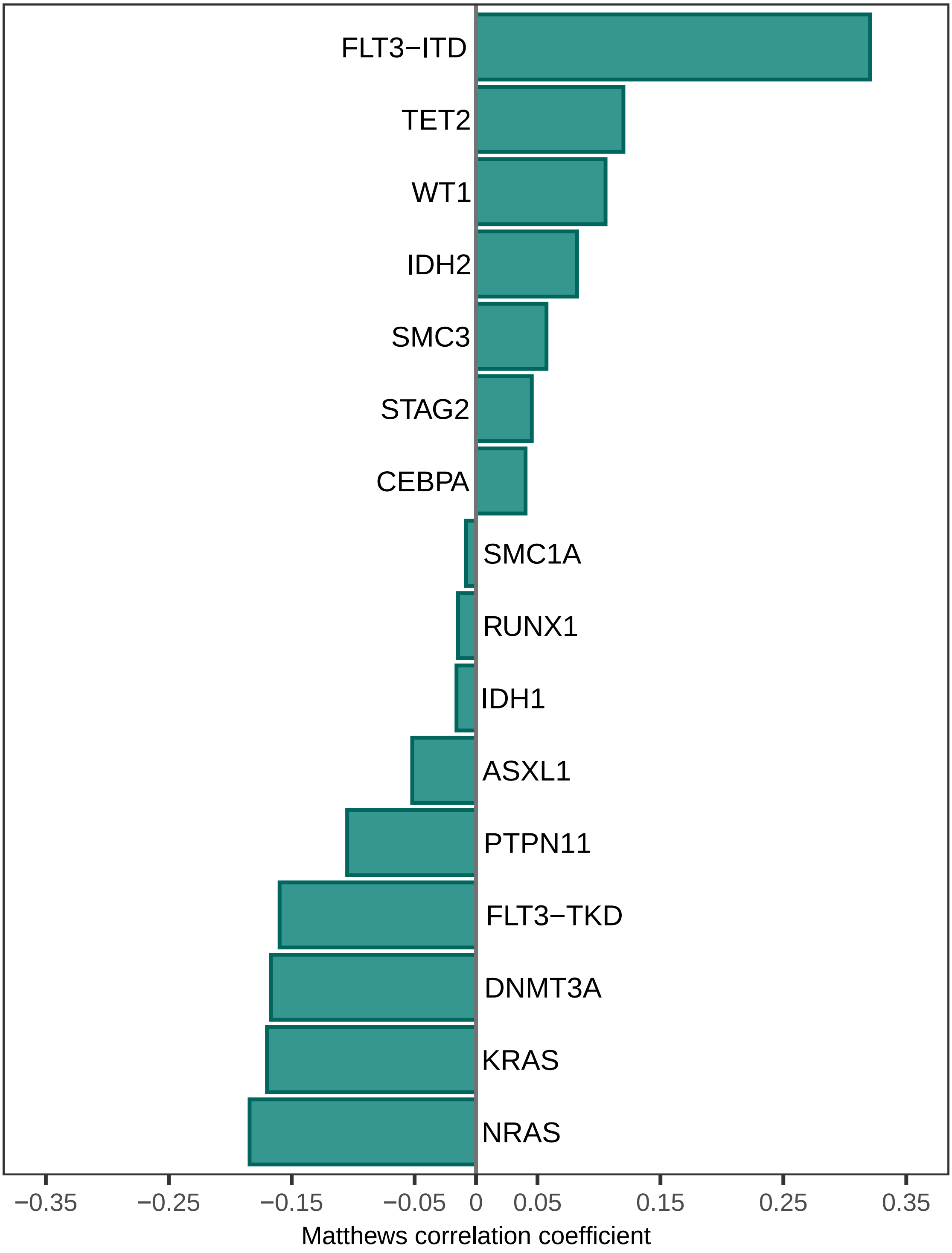

Supplementary Figure 3: Matthews correlation coefficient (MCC) between mutation status of different genes and subtypes.

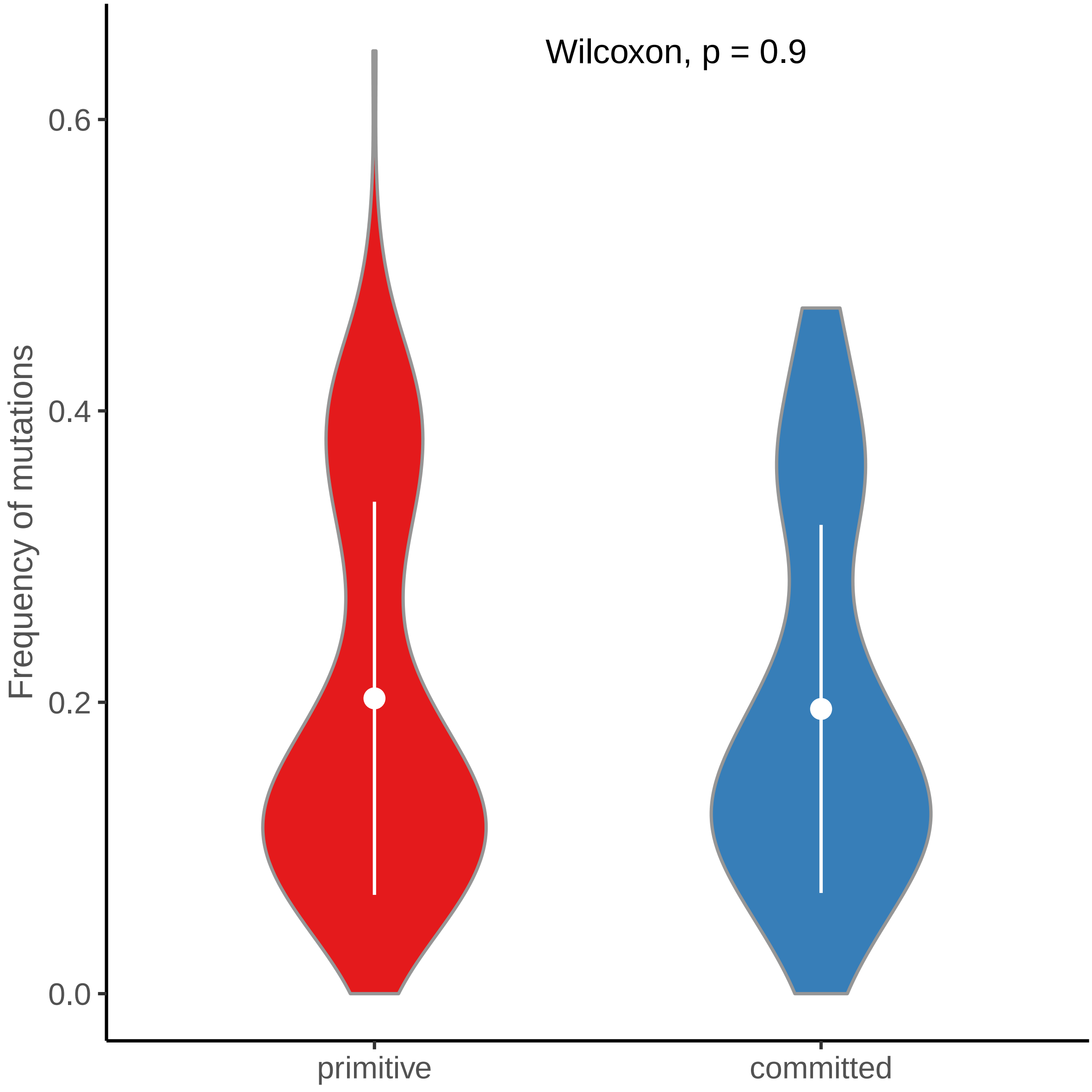

Supplementary Figure 4: Violin plot shows frequency of mutations in primitive and committed subtypes. X-axis shows primitive and committed subtypes and Y-axis represents frequency of mutations. Analysis shows that primitive and committed subtypes have the same frequency of mutation (two-sided Wilcoxon rank-sum test $p - value = 0.9$).

Such data was available only for the BeatAML cohort. Figure 5 and figure 6 show allele burden for FLT3-ITD and DNMT3A, respectively. In both cases we did not find any statistically significant difference in allele burden in primitive and committed subtypes (two-sided Wilcoxon rank-sum test $p - value > 0.05$ for both FLT3-ITD and DNMT3A).

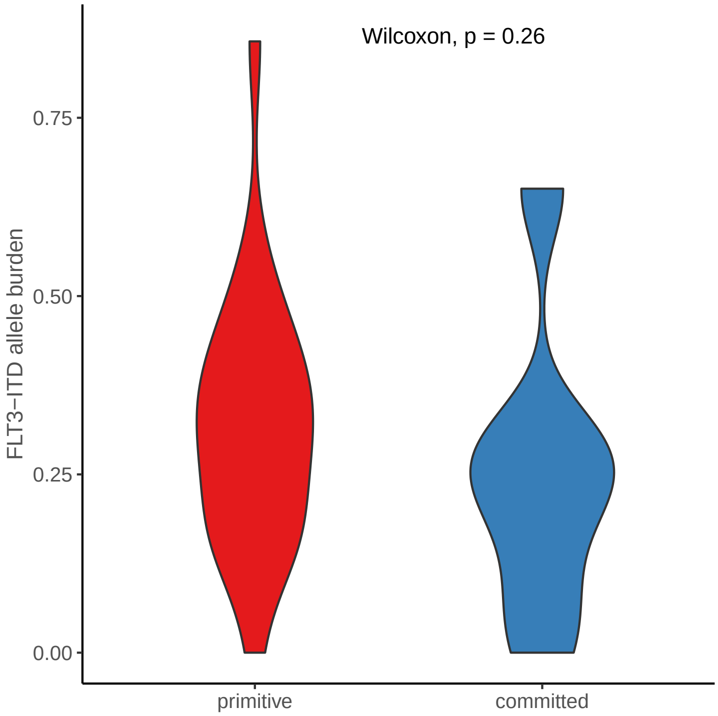

Supplementary Figure 5: Allele burden of FLT3-ITD in primitive and committed subtypes. The allele burden of FLT3-ITD is not associated with primitive and committed subtypes (two-sided Wilcoxon rank-sum test $p - value = 0.26$).

Multivariate Model for Subtypes

Next we trained a logistic regression model to predict the subtype label from mutation data. Performance of the model was assessed using leave-one-dataset-out cross-validation. Figure 7 shows performance of the model during the cross-validation using the receiver operating characteristic (ROC) curve. Multivariate mutation model shows moderate performance in predicting subtypes ($ROC AUC = 0.62$), indicating that mutations alone

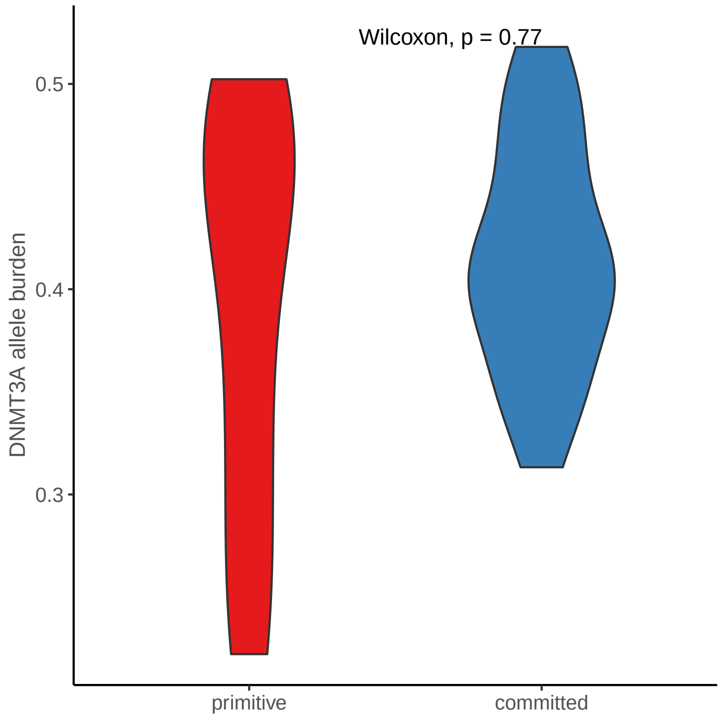

Supplementary Figure 6: Allele burden of DNMT3A in primitive and committed subtypes. The allele burden of DNMT3A is not associated with primitive and committed subtypes (two-sided Wilcoxon rank-sum test $p - value = 0.77$).

cannot define primitive and committed subtypes adequately.

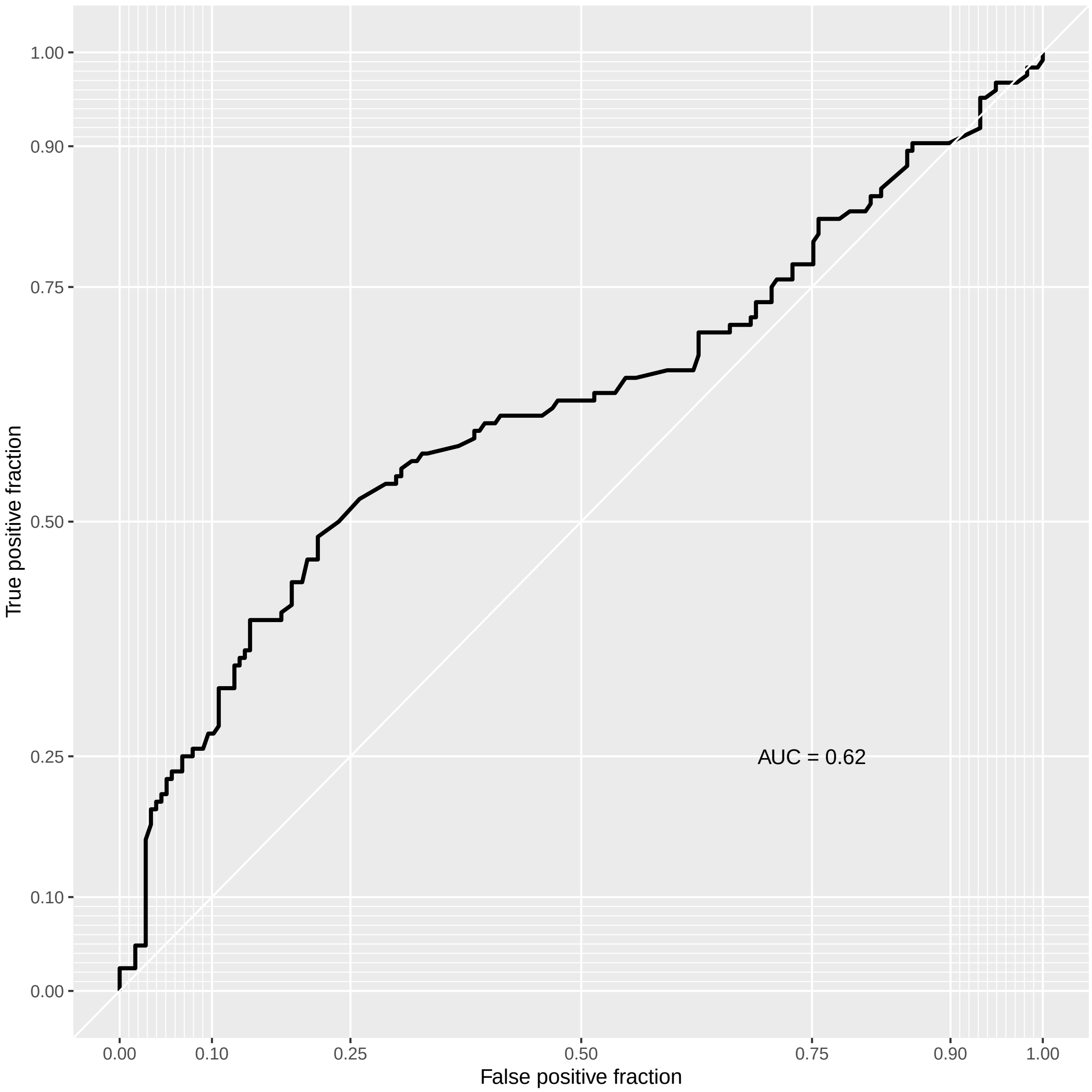

Supplementary Figure 7: Receiver Operating Characteristic (ROC) curve for multivariate mutation analysis. Logistic regression models were trained on mutation information to predict the subtypes. For the training and testing of the model a leave-one-dataset-out cross-validation strategy was applied. ROC AUC (area under curve) was computed to assess the performance of the model. Mutation based models show moderate performance in predicting subtypes (AUC=0.62).

2.2 Subtype and Mutation Gene Signatures

Differential gene expression analysis was performed using the DESeq2 R package [12, 13]. Figure 8 shows the result of differential gene expression analysis for primitive versus committed subtypes. Top differentially expressed genes such as CD14, MARCO, VCAN, FAM84A etc. are annotated in the figure. Figure 9 shows the result of differential gene expression analysis for FLT3-ITD mutated versus wild type samples. In this figure, top differentially expressed genes from subtypes (figure 8) are annotated. Similarly the volcano plot in figure 10 shows the result for DNMT3A mutated versus wild type samples.

Genes that are differentially expressed between primitive-committed subtypes are not differentially expressed between FLT3-ITD mutated-wild type groups. Same is true for DNMT3A mutated-wild type groups. This is further evident from figure 11, figure 12, figure 13 and figure 14, which show expression of genes *C1QA*, *MARCO*, *ZNF521* and *CDH2* in individual patient cohorts. Venn diagrams in figure 15 summarize the number of differentially expressed genes in subtype and mutation groups.

From these results it is evident that primitive-committed subtypes have unique gene signature which are not captured by FLT3-ITD or DNMT3A mutations.

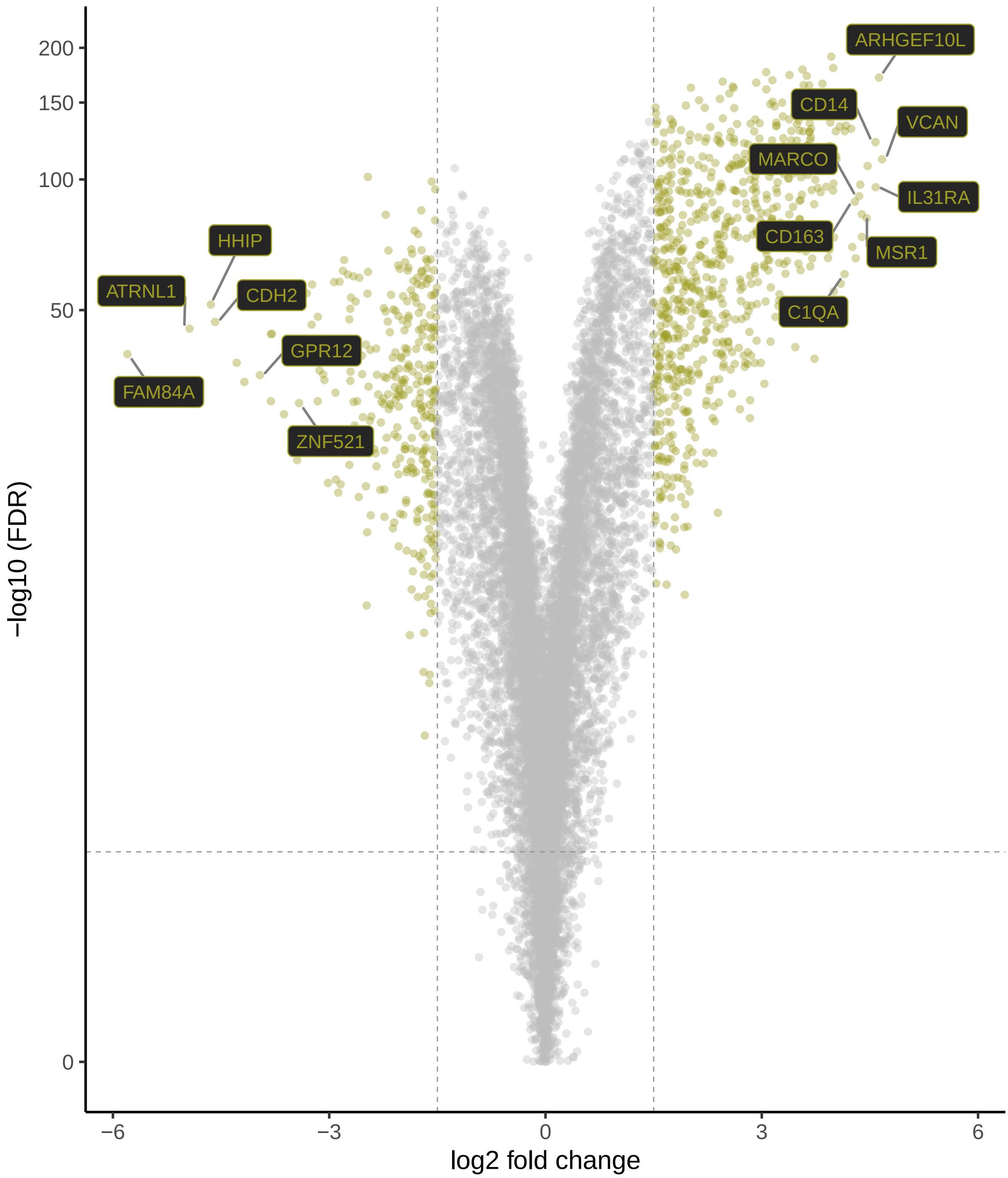

Supplementary Figure 8: Volcano plot for primitive vs. committed subtypes. Differential gene expression analysis was performed between primitive and committed subtypes. The x-axis represents $\log_2$ fold change and y-axis shows $\log_{10}$ FDR values for genes. Each point in the plot represents one gene. Points in lime color indicate genes with high fold change and $FDR < 0.01$. Top genes are annotated in the plot.

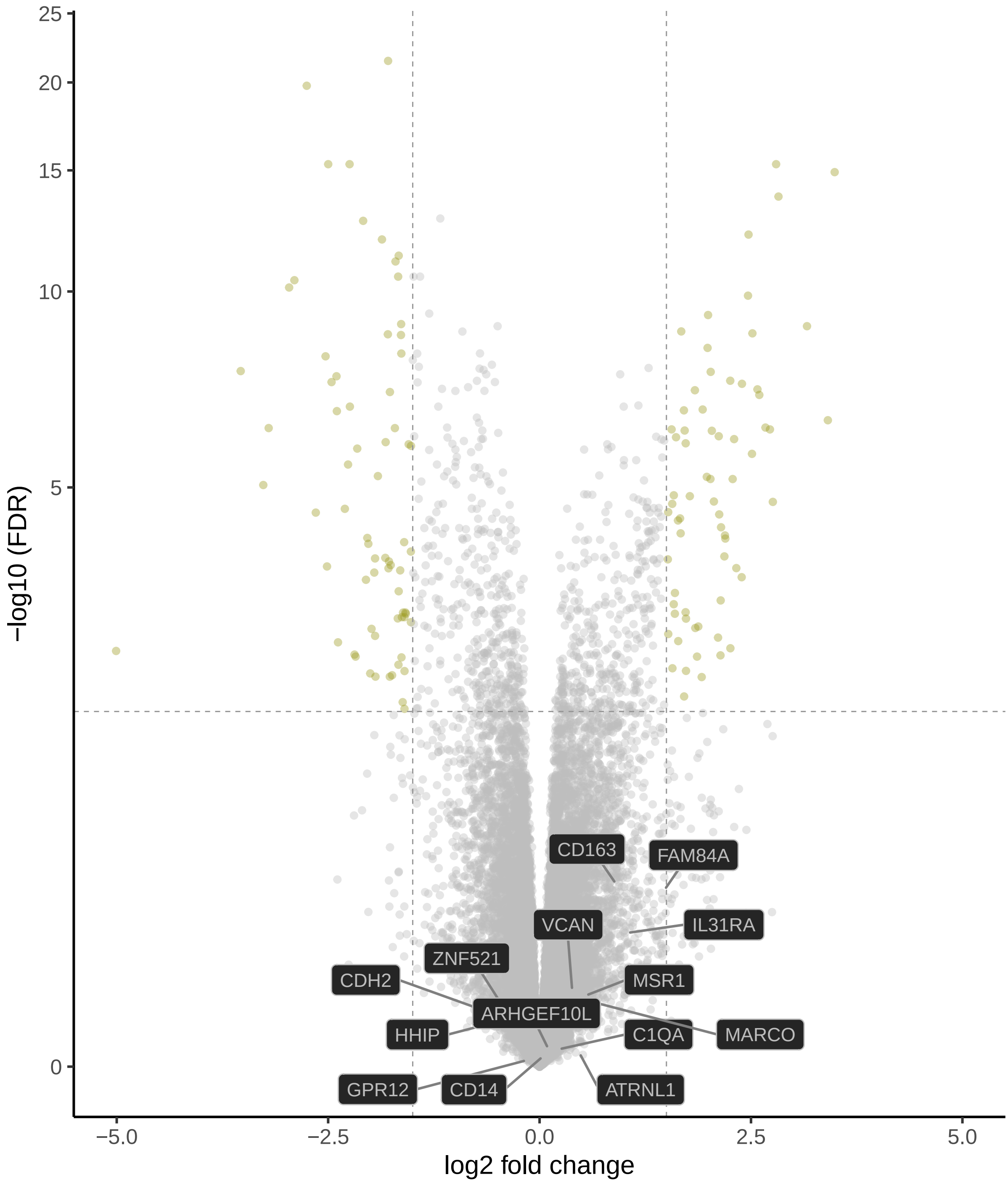

Supplementary Figure 9: Volcano plot for FLT3-ITD mutated vs. wild type samples. The x-axis represents $\log_2$ fold change and y-axis shows $\log_{10}$ FDR values for genes. Each point in the plot represents one gene. Points in lime color indicate genes with high fold change and $FDR < 0.01$. Genes specific to the primitive and committed subtypes (see Supplementary Figure 8) are annotated in the plot.

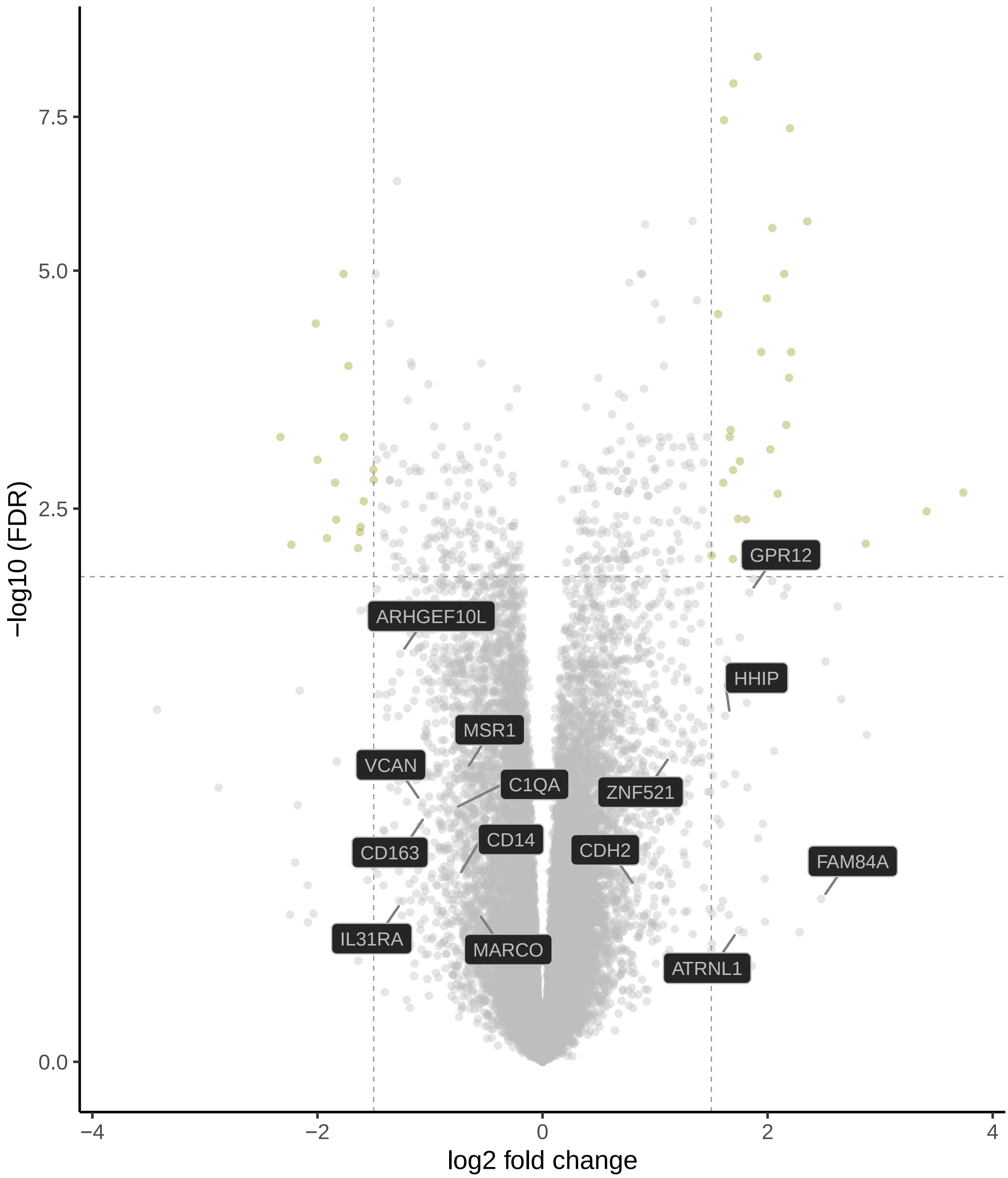

Supplementary Figure 10: Volcano plot for DNMT3A mutated vs. wild type samples. The x-axis represents $\log_2$ fold change and y-axis shows $\log_{10}$ FDR values for genes. Each point in the plot represents one gene. Points in lime color indicate genes with high fold change and $FDR < 0.01$. Genes specific to the primitive and committed subtypes (see Supplementary Figure 8) are annotated in the plot.

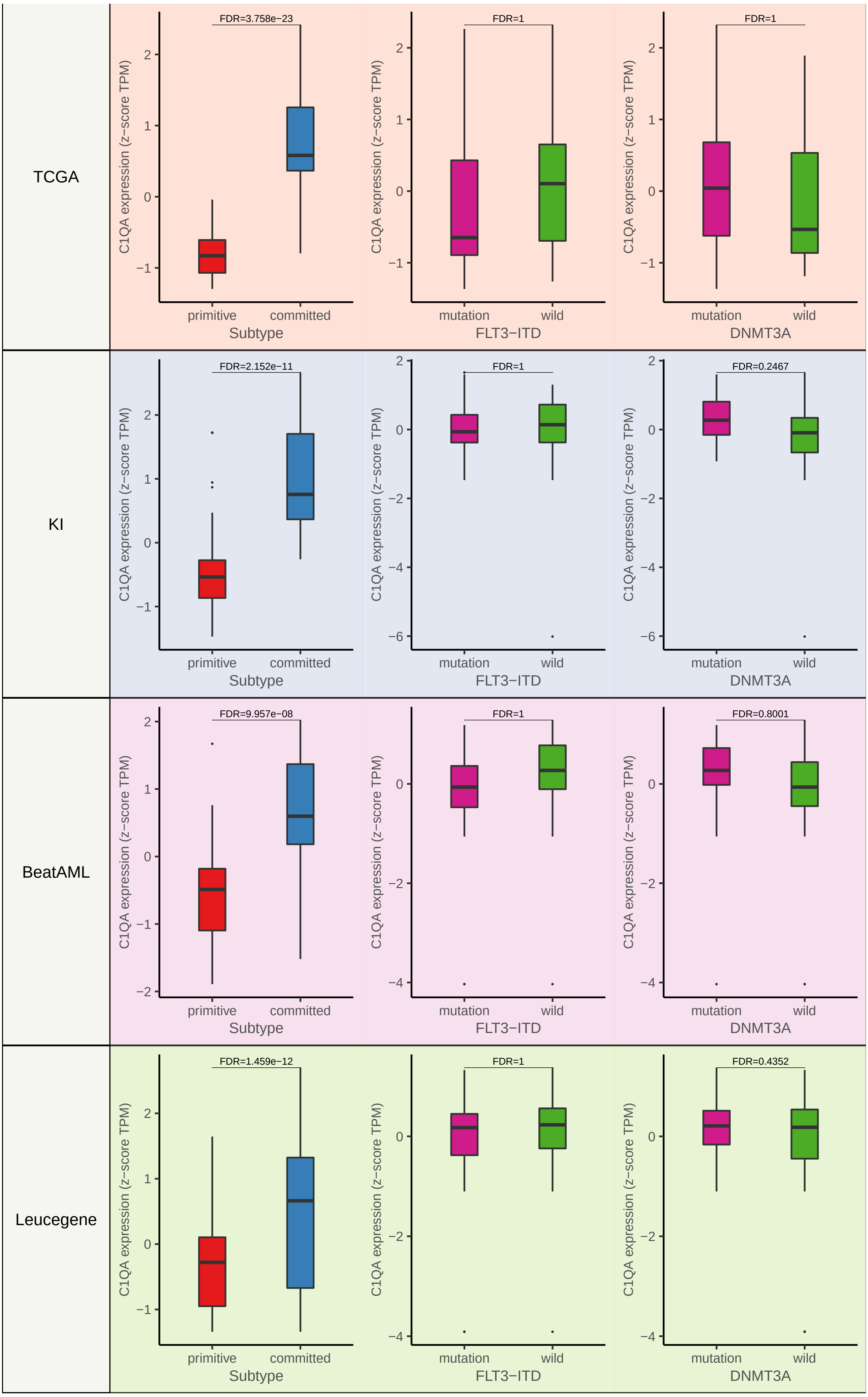

Supplementary Figure 11: Expression of C1QA in four datasets. Each boxplot represents expression of the gene (TPM values) in a group (TCGA primitive $n = 23$; TCGA committed $n = 25$; KI primitive $n = 55$; KI committed $n = 24$; BeatAML primitive $n = 43$; BeatAML committed $n = 39$; Leucegene primitive $n = 60$; Leucegene committed $n = 38$). First barplots are for subtypes, second barplots for FLT3-ITD and third barplots for DNMT3A groups. The values on the top of the barplots indicate FDR of differential gene expression analysis (two-sided t-test). In the boxplots, middle line indicates median. The lower and upper hinges of the boxes correspond to the 25th and 75th percentiles. The whiskers represent 1.5 x IQR from the hinge (where IQR is the inter-quartile range). Data beyond the end of the whiskers are outlying points that are plotted individually.

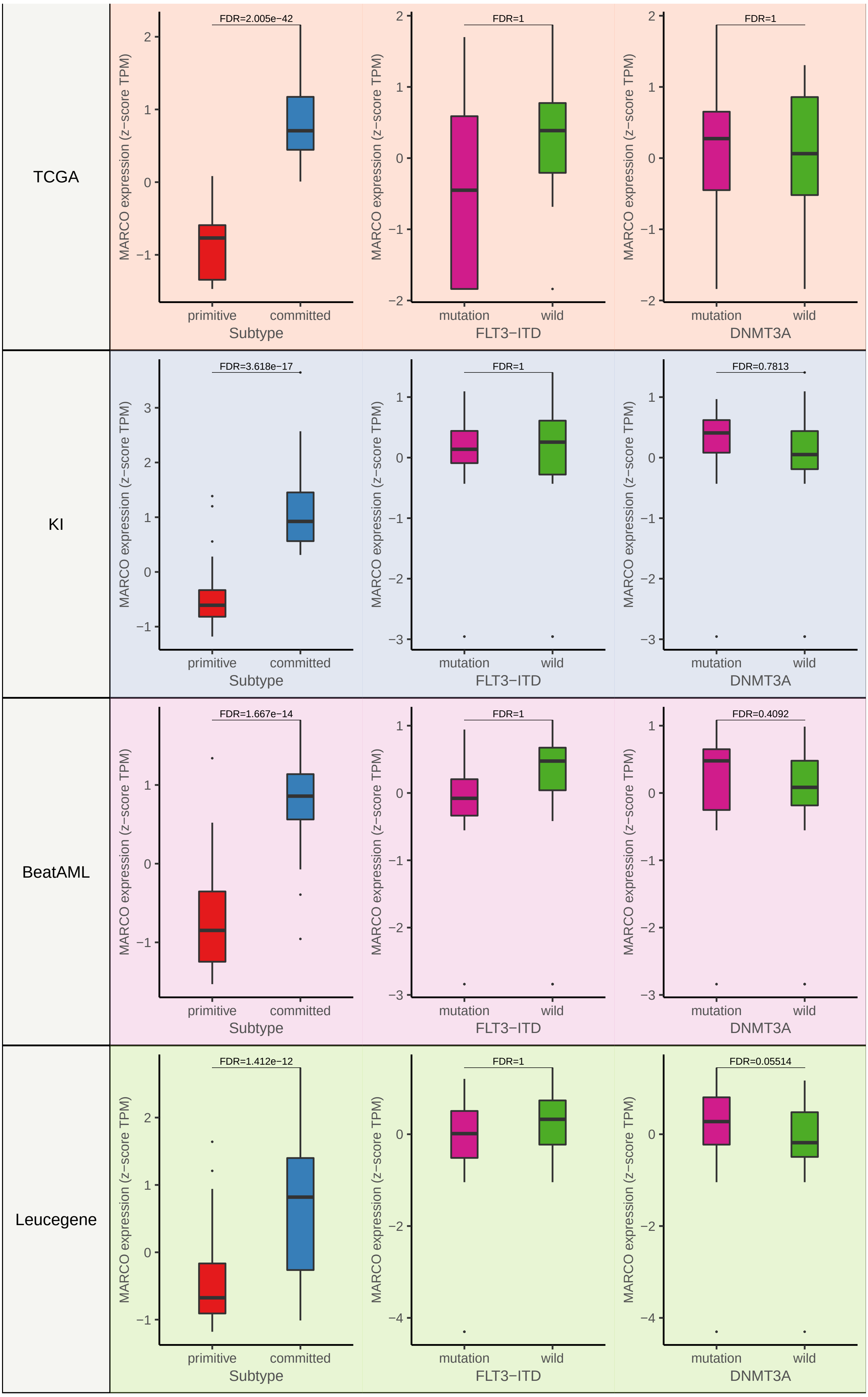

Supplementary Figure 12: Expression of MARCO in four datasets. Each boxplot represents expression of the gene (TPM values) in a group (TCGA primitive *n = 23*; TCGA committed *n = 25*; KI primitive *n = 55*; KI committed *n = 24*; BeatAML primitive *n = 43*; BeatAML committed *n = 39*; Leucegene primitive *n = 60*; Leucegene committed *n = 38*). First barplots are for subtypes, second barplots for FLT3-ITD and third barplots for DNMT3A groups. The values on the top of the barplots indicate FDR of differential gene expression analysis (two-sided t-test). In the boxplots, middle line indicates median. The lower and upper hinges of the boxes correspond to the 25th and 75th percentiles. The whiskers represent 1.5 x IQR from the hinge (where IQR is the inter-quartile range). Data beyond the end of the whiskers are outlying points that are plotted individually.

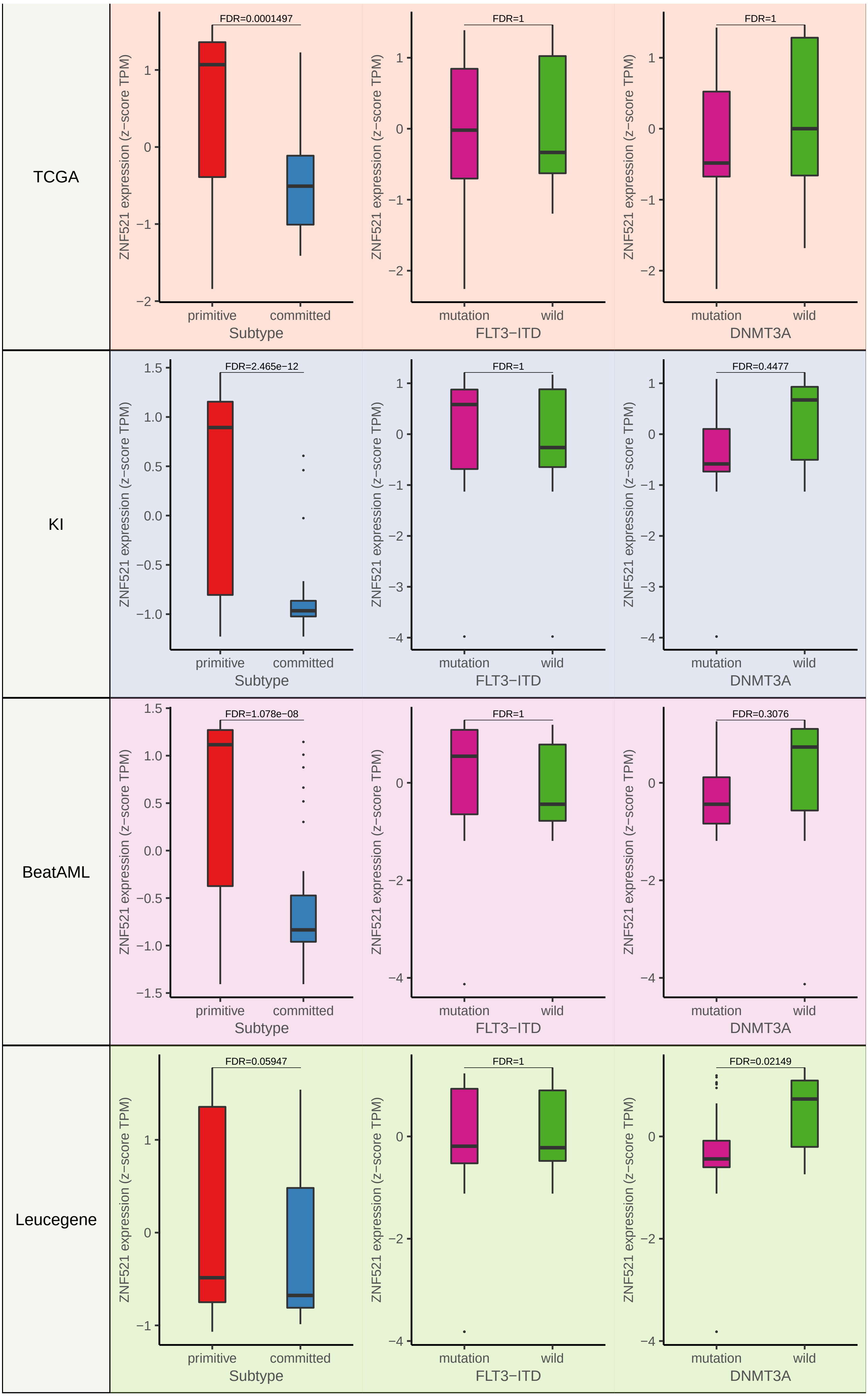

Supplementary Figure 13: Expression of ZNF521 in four datasets. Each boxplot represents expression of the gene (TPM values) in a group (TCGA primitive *n = 23*; TCGA committed *n = 25*; KI primitive *n = 55*; KI committed *n = 24*; BeatAML primitive *n = 43*; BeatAML committed *n = 39*; Leucegene primitive *n = 60*; Leucegene committed *n = 38*). First barplots are for subtypes, second barplots for FLT3-ITD and third barplots for DNMT3A groups. The values on the top of the barplots indicate FDR of differential gene expression analysis (two-sided t-test). In the boxplots, middle line indicates median. The lower and upper hinges of the boxes correspond to the 25th and 75th percentiles. The whiskers represent 1.5 x IQR from the hinge (where IQR is the inter-quartile range). Data beyond the end of the whiskers are outlying points that are plotted individually.

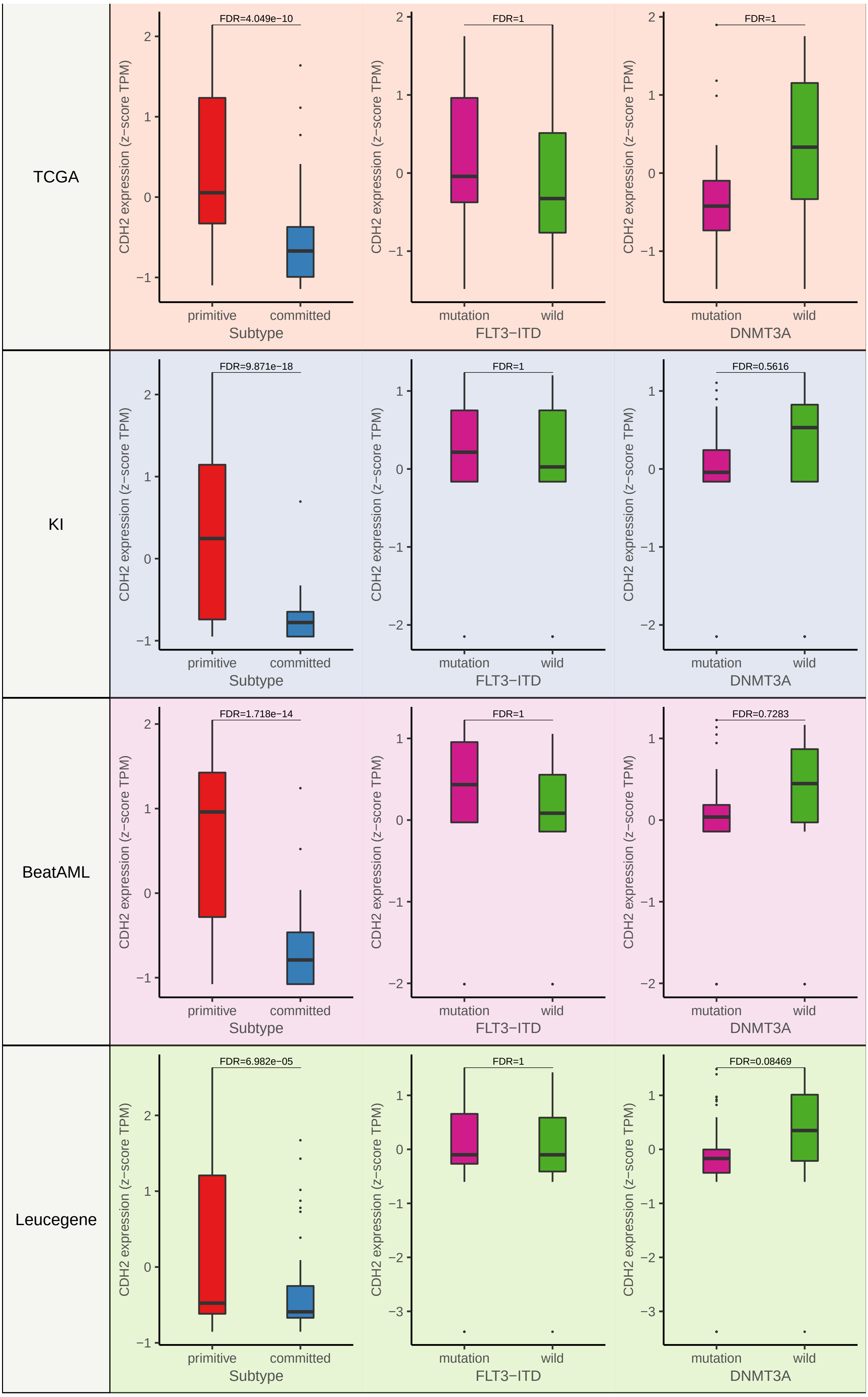

Supplementary Figure 14: Expression of CDH2 in four datasets. Each boxplot represents expression of the gene (TPM values) in a group (TCGA primitive *n = 23*; TCGA committed *n = 25*; KI primitive *n = 55*; KI committed *n = 24*; BeatAML primitive *n = 43*; BeatAML committed *n = 39*; Leucegene primitive *n = 60*; Leucegene committed *n = 38*). First barplots are for subtypes, second barplots for FLT3-ITD and third barplots for DNMT3A groups. The values on the top of the barplots indicate FDR of differential gene expression analysis (two-sided t-test). In the boxplots, middle line indicates median. The lower and upper hinges of the boxes correspond to the 25th and 75th percentiles. The whiskers represent 1.5 x IQR from the hinge (where IQR is the inter-quartile range). Data beyond the end of the whiskers are outlying points that are plotted individually.

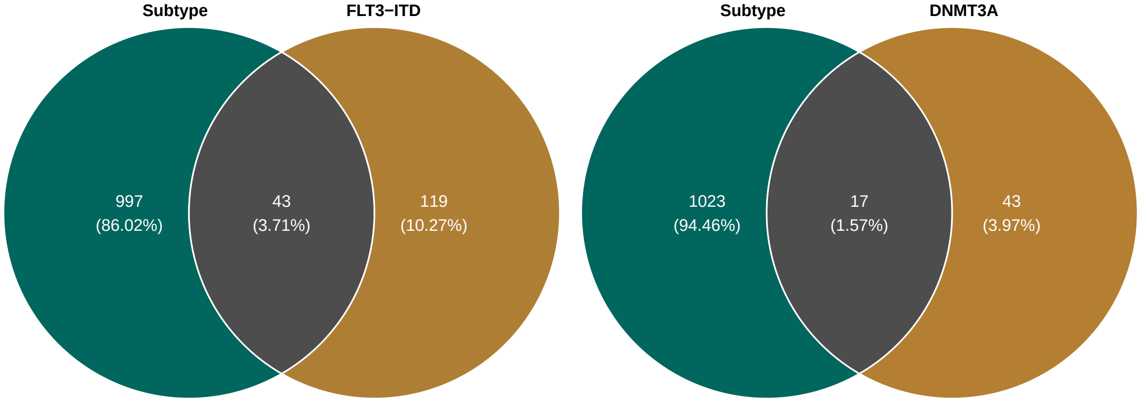

(a) (b)

Supplementary Figure 15: Venn diagrams show number of differentially expressed genes in subtypes (primitive versus committed) and mutation groups (mutated versus wildtype). In total 1040 genes are differentially expressed between primitive and committed subtypes. Figure (a) shows that between FLT3-ITD mutated and wild type, 162 genes are differentially expressed out of which only 43 genes are common with subtype specific differentially expressed genes. Figure (b) shows that between DNMT3A mutated and wild type, 60 genes are differentially expressed out of which only 17 genes are common with subtype specific differentially expressed genes.

2.3 Subtype and Mutations Pathway Analysis

Gene set enrichment analysis was performed for subtypes, FLT3-ITD mutation and DNMT3A mutation. Figure 16 shows pathways that are significantly enriched ($FDR < 0.05$) in subtypes. It also shows the corresponding enrichment score and p-value of selected pathways (enriched in subtypes), in the FLT3-ITD and DNMT3A mutation groups. Pathways that have a high enrichment score for subtypes are not enriched in FLT3-ITD or DNMT3A mutation groups ($FDR > 0.05$ for all pathways). These results show that subtypes have uniquely activated pathways compared to FLT3-ITD or DNMT3A mutation.

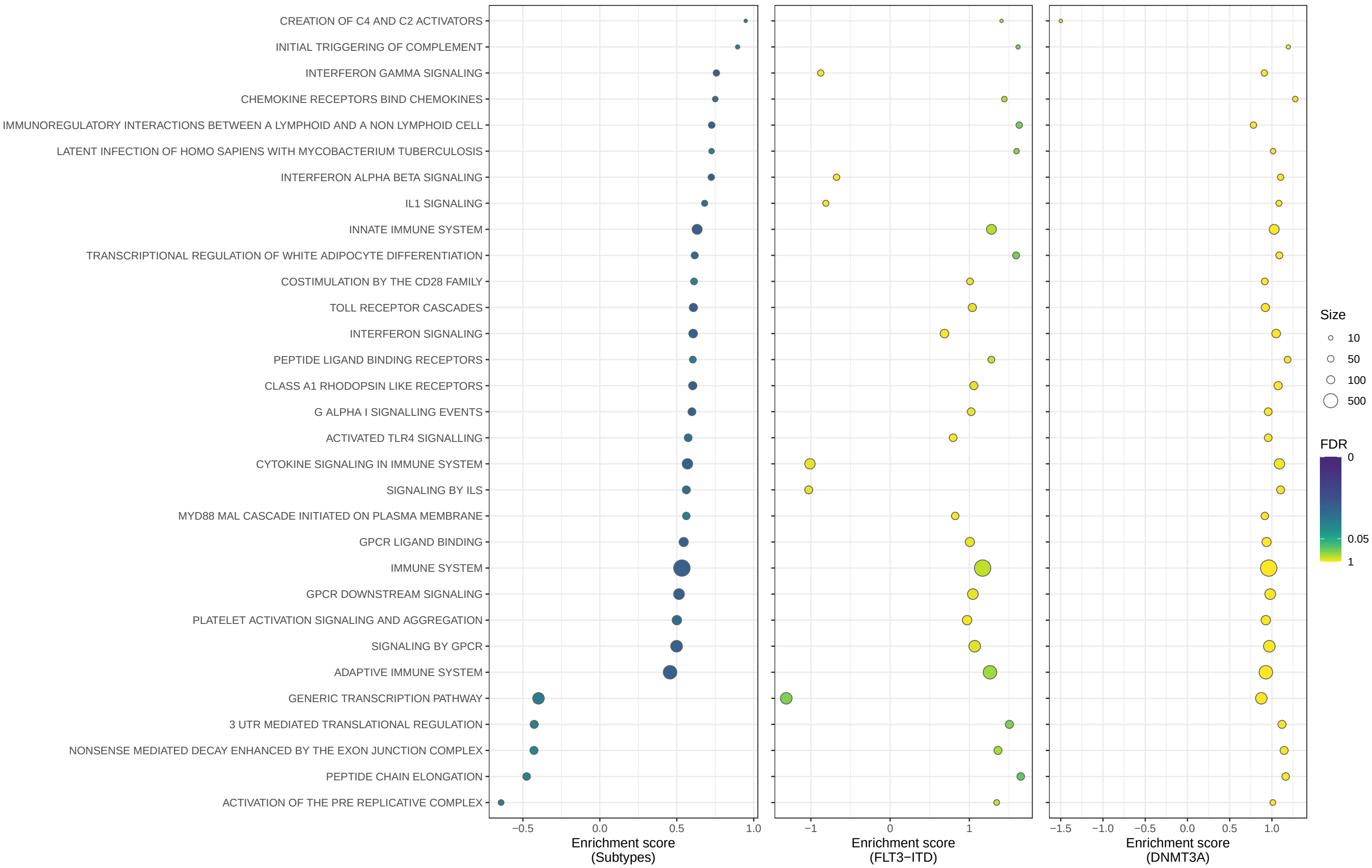

Supplementary Figure 16: Dotplots for pathway enrichment analysis. Top pathways that are enriched in subtypes ($FDR < 0.05$) are shown on the y-axis. The x-axis represents enrichment score for the pathway. Size of dots is proportional to the number of genes in the pathway. Color of the dots represents FDR value for the pathway. First panel shows enrichment of the pathways in subtypes (primitive versus committed). Dotplots in second panel show enrichment of the pathways in FLT3-ITD mutated versus wild type groups. Third panel shows enrichment of the pathways in DNMT3A mutated versus wild type groups. Pathways that are enriched in subtypes do not show significant enrichment in FLT3-ITD or DNMT3A groups. This indicates that subtypes are driven by distinct pathways.

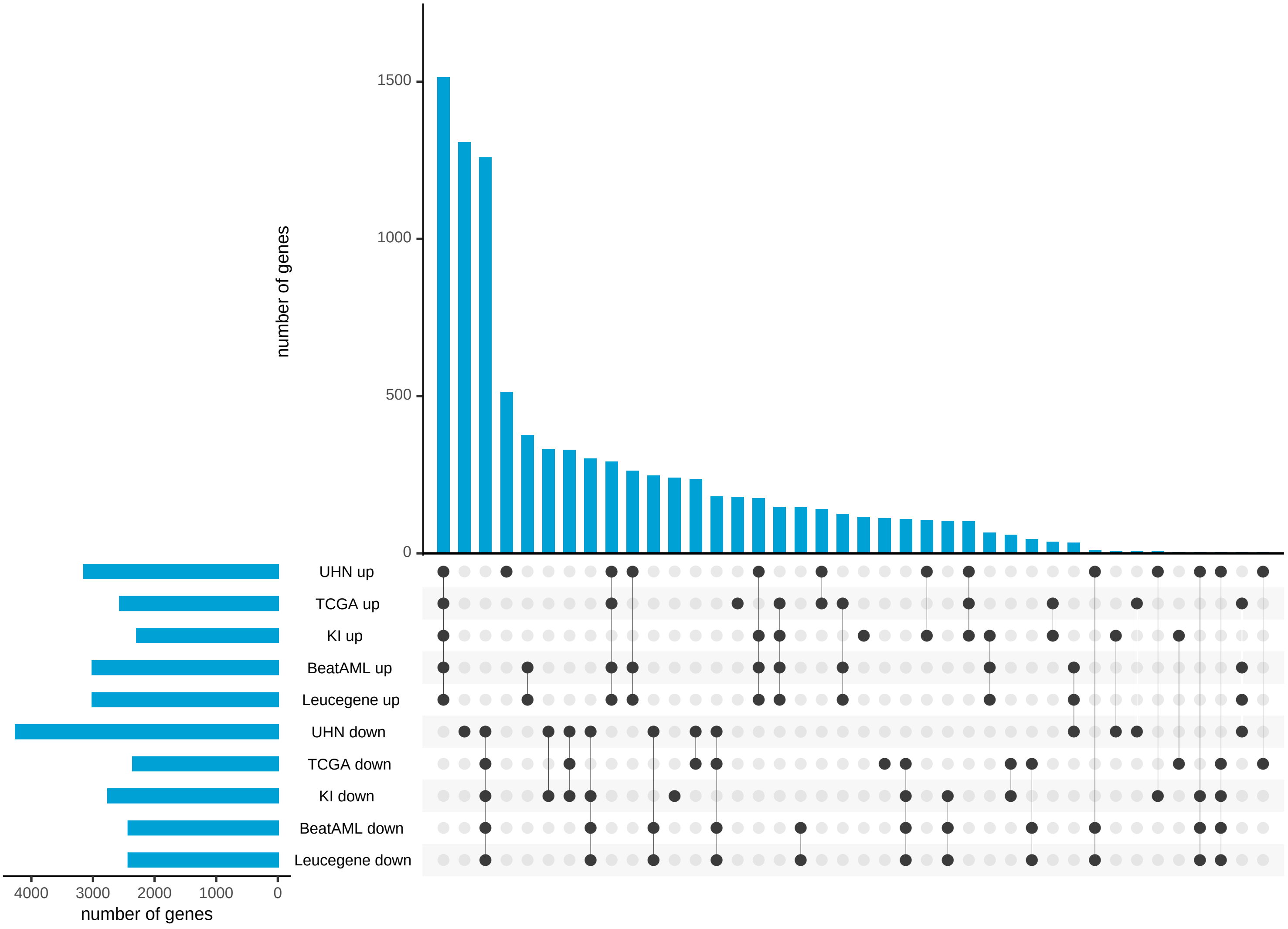

Supplementary Figure 17: Upset plot showing differentially expressed genes across subtypes and five datasets.

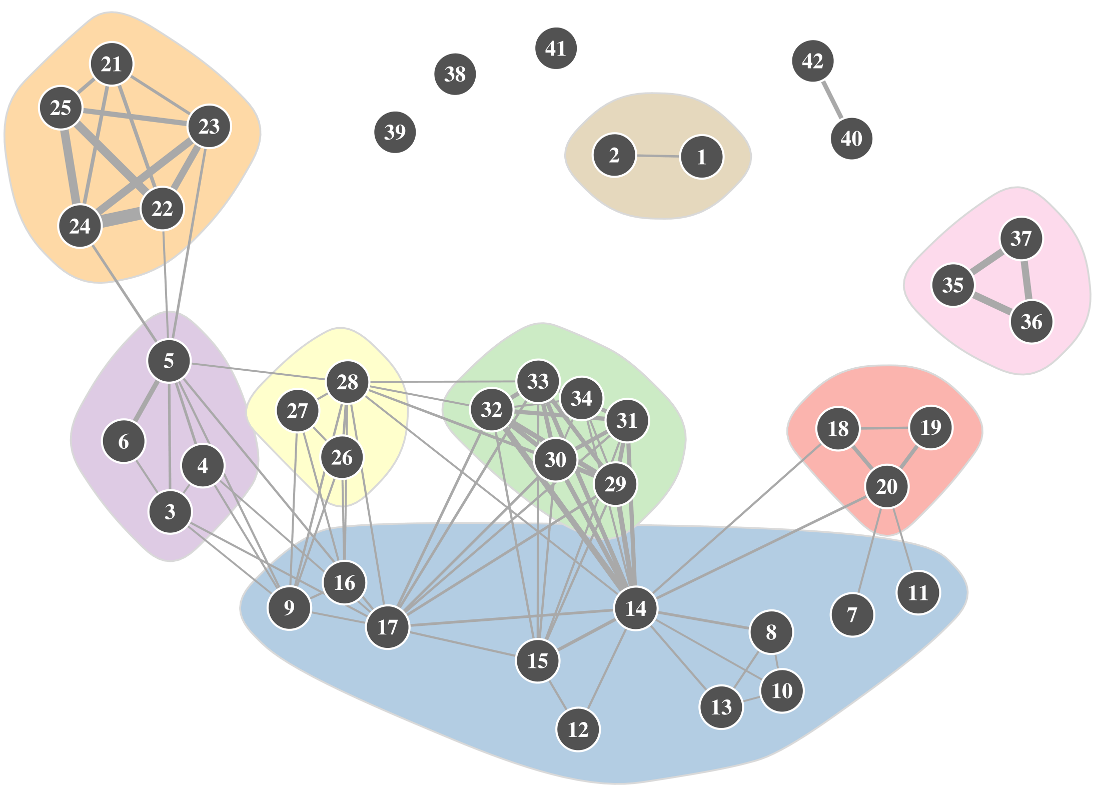

index	REACTOME pathway
1	ACTIVATION OF ATR IN RESPONSE TO REPLICATION STRESS
2	ACTIVATION OF THE PRE REPLICATIVE COMPLEX
3	CELL SURFACE INTERACTIONS AT THE VASCULAR WALL
4	GPVI MEDIATED ACTIVATION CASCADE
5	PLATELET ACTIVATION SIGNALING AND AGGREGATION
6	RESPONSE TO ELEVATED PLATELET CYTOSOLIC CA2
7	ANTIGEN PROCESSING CROSS PRESENTATION
8	COMPLEMENT CASCADE
9	COSTIMULATION BY THE CD28 FAMILY
10	CREATION OF C4 AND C2 ACTIVATORS
11	IMMUNOREGULATORY INTERACTIONS BETWEEN A LYMPHOID AND A NON LYMPHOID CELL
12	INFLAMMASOMES
13	INITIAL TRIGGERING OF COMPLEMENT
14	INNATE IMMUNE SYSTEM
15	NUCLEOTIDE BINDING DOMAIN LEUCINE RICH REPEAT CONTAINING RECEPTOR NLR SIGNALING PATHWAYS
16	PD1 SIGNALING
17	SIGNALING BY ILS
18	INTERFERON ALPHA BETA SIGNALING
19	INTERFERON GAMMA SIGNALING
20	INTERFERON SIGNALING
21	CHEMOKINE RECEPTORS BIND CHEMOKINES
22	CLASS A1 RHODOPSIN LIKE RECEPTORS
23	G ALPHA I SIGNALLING EVENTS
24	GPCR LIGAND BINDING
25	PEPTIDE LIGAND BINDING RECEPTORS
26	GENERATION OF SECOND MESSENGER MOLECULES
27	PHOSPHORYLATION OF CD3 AND TCR ZETA CHAINS
28	TCR SIGNALING
29	ACTIVATED TLR4 SIGNALLING
30	MYD88 MAL CASCADE INITIATED ON PLASMA MEMBRANE
31	NFKB AND MAP KINASES ACTIVATION MEDIATED BY TLR4 SIGNALING REPERTOIRE
32	TOLL RECEPTOR CASCADES
33	TRAF6 MEDIATED INDUCTION OF NFKB AND MAP KINASES UPON TLR7 8 OR 9 ACTIVATION
34	TRAF6 MEDIATED INDUCTION OF TAK1 COMPLEX
35	3 UTR MEDIATED TRANSLATIONAL REGULATION
36	NONSENSE MEDIATED DECAY ENHANCED BY THE EXON JUNCTION COMPLEX
37	PEPTIDE CHAIN ELONGATION
38	TRANSCRIPTIONAL REGULATION OF WHITE ADIPOCYTE DIFFERENTIATION
39	LATENT INFECTION OF HOMO SAPIENS WITH MYCOBACTERIUM TUBERCULOSIS
40	RESPIRATORY ELECTRON TRANSPORT ATP SYNTHESIS BY CHEMIOSMOTIC COUPLING AND HEAT PRODUCTION BY UNCOUPLING PROTEINS
41	THE ROLE OF NEF IN HIV1 REPLICATION AND DISEASE PATHOGENESIS
42	RESPIRATORY ELECTRON TRANSPORT

Supplementary Figure 18: Network based visualization of pathway enrichment analysis for subtypes (related to *main Figure-2C*). Each node represents a pathway and the edge represents common genes between pathways. Size of the nodes and edges is proportional to the number of genes in the pathway and common genes, respectively. Pathways represented by each node are listed in the table.

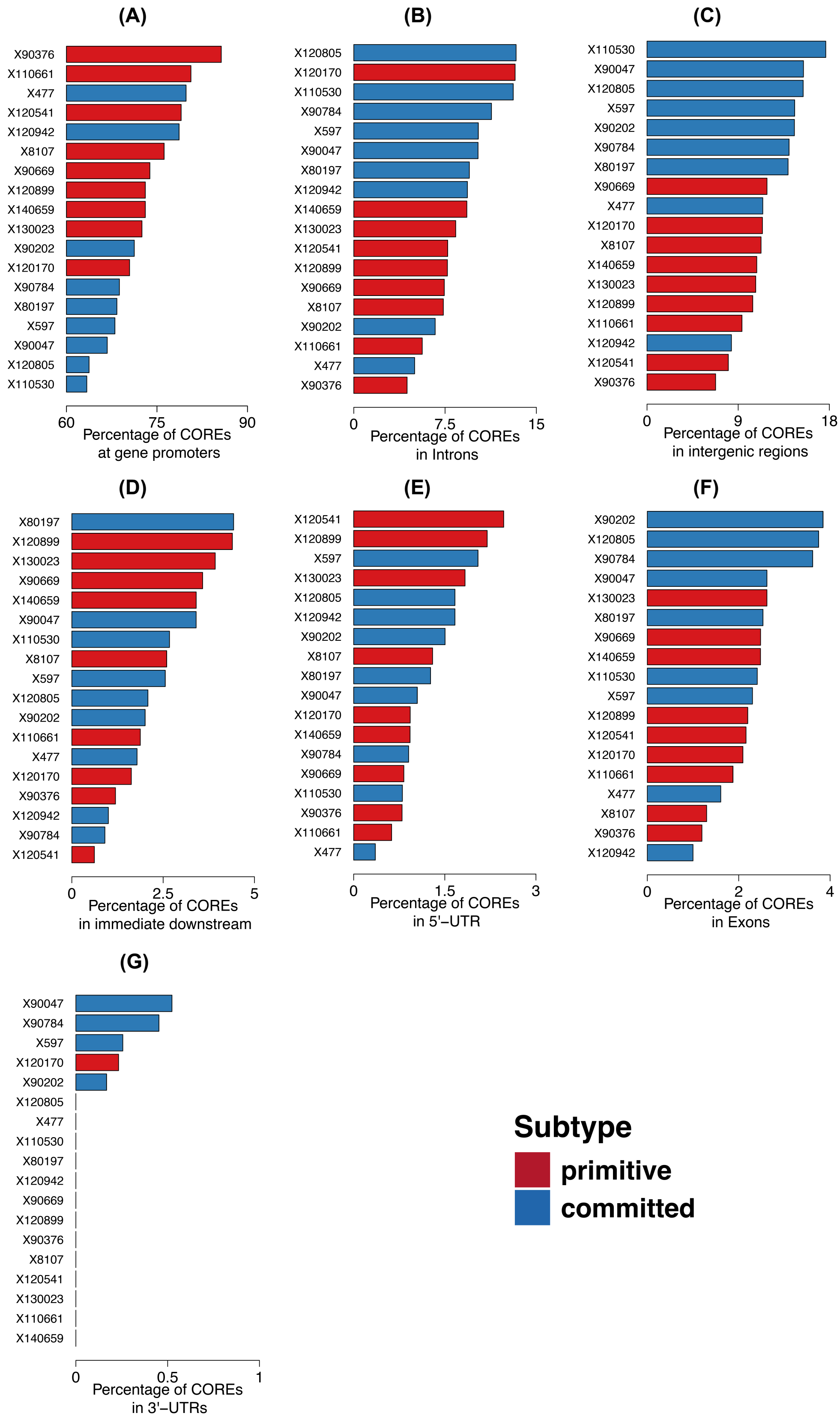

Supplementary Figure 19: Comparison of proportion of COREs at different genomic regions in primitive versus committed subtypes in the ATAC-seq profile. (A) Promoters (FDR=0.11), (B) introns (FDR=0.47), (C) intergenic region (FDR=0.02) (D) immediate downstream (FDR=1), (E) 5' UTR (FDR=1), (F) Exons (FDR=0.47) and (G) 3' UTR (FDR=0.47)

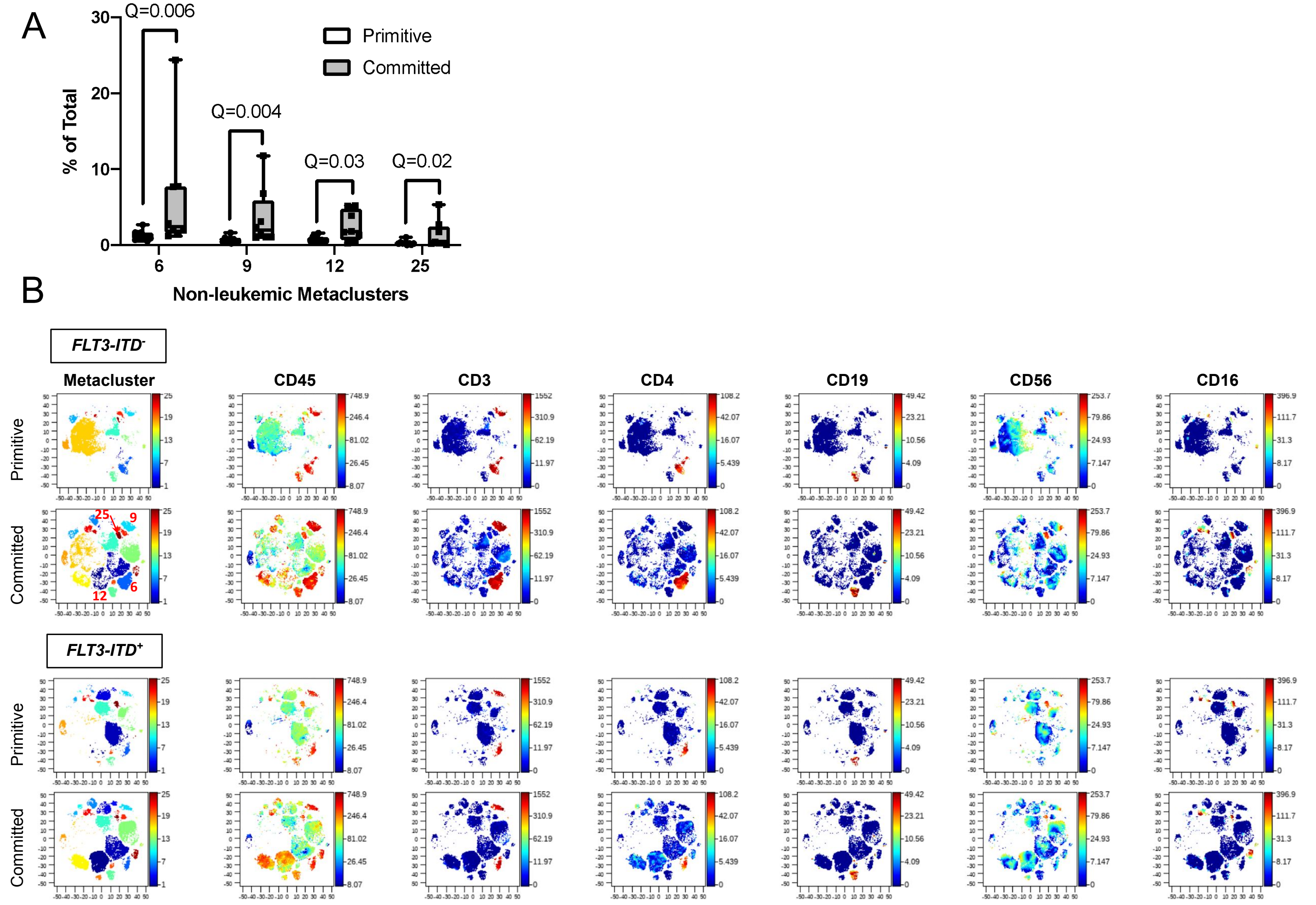

Supplementary Figure 20: Quantification and t-SNE visualization of normal non-leukemic FlowSom metaclusters in representative primitive versus committed $FLT3\text{-}ITD^{-}$ and $FLT3\text{-}ITD^{+}$ cases of NPM1 mutated AML. (A) Box and whisker plots of non-leukemic metaclusters that were differentially abundant between the primitive and committed groups in the *diffcyt* analysis [14] (*n = 9* in primitive and *n = 8* in committed group). The *Q-values* for each from the *diffcyt-DA-edgeR* analysis are indicated. In the boxplots, middle line indicates median. The lower and upper hinges of the boxes correspond to the 25th and 75th percentiles. The whiskers represent 1.5 x IQR from the hinge (where IQR is the inter-quartile range). Data beyond the end of the whiskers are outlying points that are plotted individually. (B) t-SNE plots are colored in the Z dimension by FlowSom [15] Metacluster ID or the indicated cell surface markers. The intensity scale (lowest: dark blue; highest: red) for each surface marker is shown on the right of each plot. From these plots it can be seen that these metaclusters consist of $CD45^{hi}$ cells that are either $CD3^{+}$ $CD4^{+}$ T-cells (#6), $CD3^{+}$ $CD4^{-}$ T-cells (#9), $CD19^{+}$ B-cells (#12) or $CD3^{-}$ $CD56^{+}$ $CD16^{+}$, NK cells (#25).

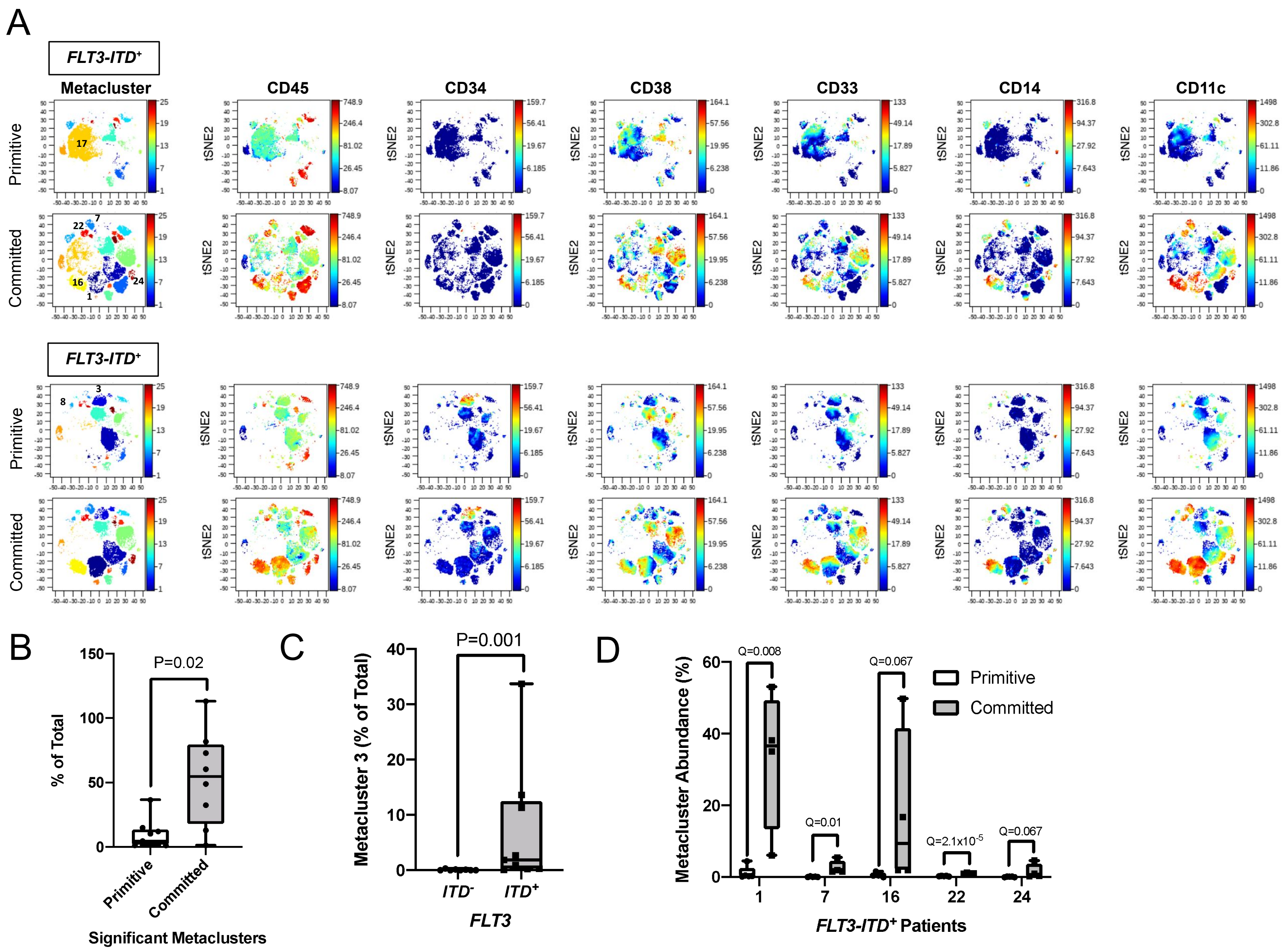

Supplementary Figure 21: Quantification and t-SNE visualization of leukemic FlowSom [15] metaclusters in representative primitive versus committed $FLT3\text{-}ITD^{-}$ and $FLT3\text{-}ITD^{+}$ cases of NPM1 mutated AML. (A) t-SNE plots are colored in the Z dimension by FlowSom [15] Metacluster ID or the indicated myelomonocytic markers typically expressed by AML. The intensity scale (lowest: dark blue; highest: red) for each surface marker is shown on the right of each plot. (B) Box and whisker plots show abundance of the clusters that were significantly higher in primitive cases (3 and 8) compared to those that were significantly higher in committed cases (1, 7, 16, 22 and 24). P-value computed by Mann-Whitney and *n = 9* in primitive and *n = 8* in committed group. (C) Box and whisker plots show abundance of metacluster 3 in $FLT3\text{-}ITD^{-}$ (*n = 8*) and $FLT3\text{-}ITD^{+}$ (*n = 9*) cases. P-value computed by Mann-Whitney. (D) Box and whisker plots show abundance of the indicated metaclusters primitive versus committed in $FLT3\text{-}ITD^{+}$ cases (*n = 5* in primitive and *n = 4* in committed group). Q values were computed in Prism by multiple t-tests with FDR correction by the two-stage step-up method of Benjamini, Hochberg and Yekutieli. $Q\text{-}values < 0.05$ were considered significant. In the boxplots of figure (B), (C) and (D), middle line indicates median. The lower and upper hinges of the boxes correspond to the 25th and 75th percentiles. The whiskers represent 1.5 x IQR from the hinge (where IQR is the inter-quartile range). Data beyond the end of the whiskers are outlying points that are plotted individually.

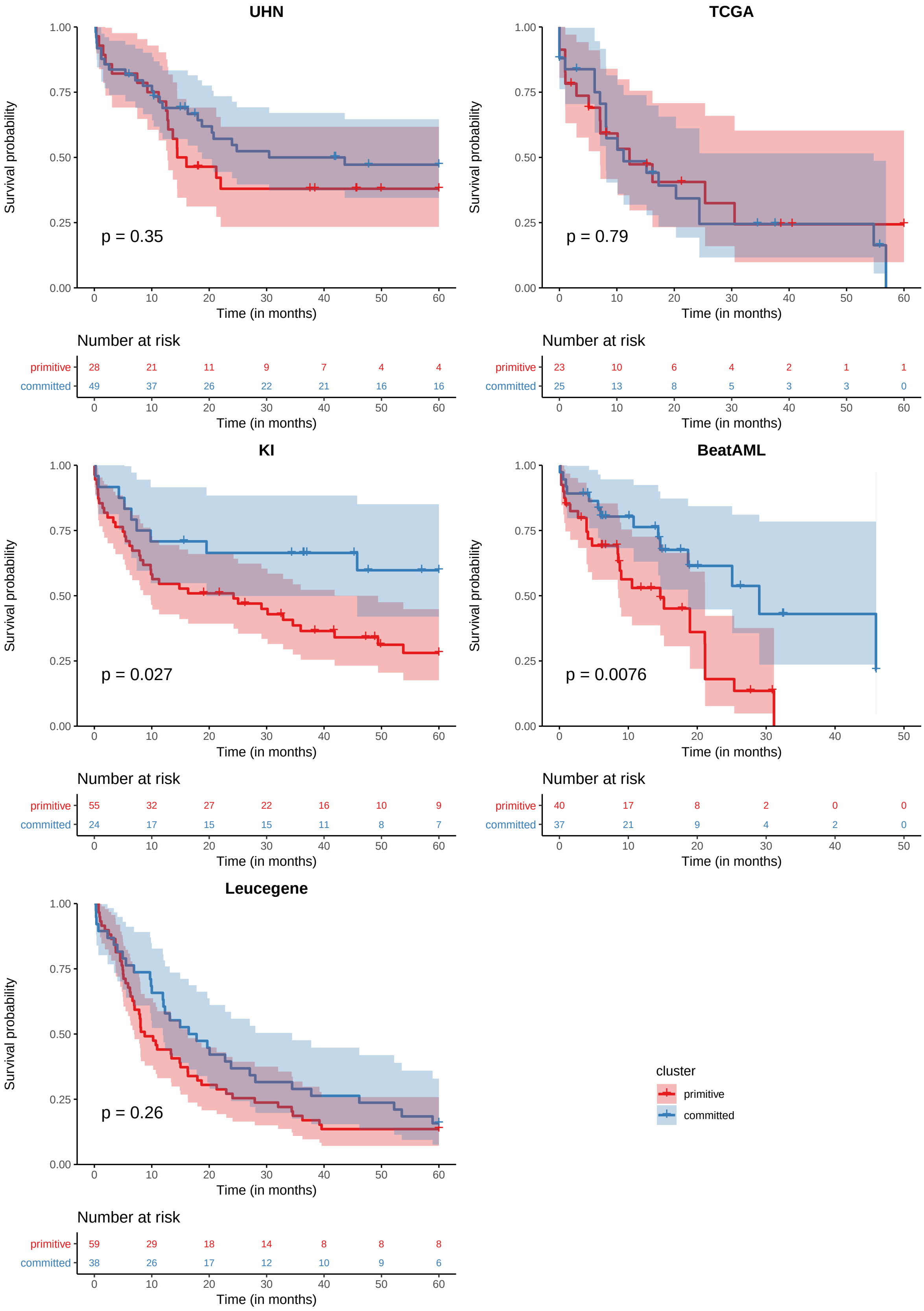

Supplementary Figure 22: Kaplan-Meier plot stratified by primitive and committed subtype in the UHN, TCGA, KI, BeatAML and Leucegene cohort. Log-rank (Mantel-Cox) test p-values are indicated for each dataset in the plot.

2.4 Drug Prioritization

We aimed to select drugs which could be potentially effective for the primitive subtype and which can be further evaluated using ex vivo drug screening. Towards this end we utilized the CCLE-CTRPv2 dataset [16]. The pipeline for drug ranking is shown in figure 23.

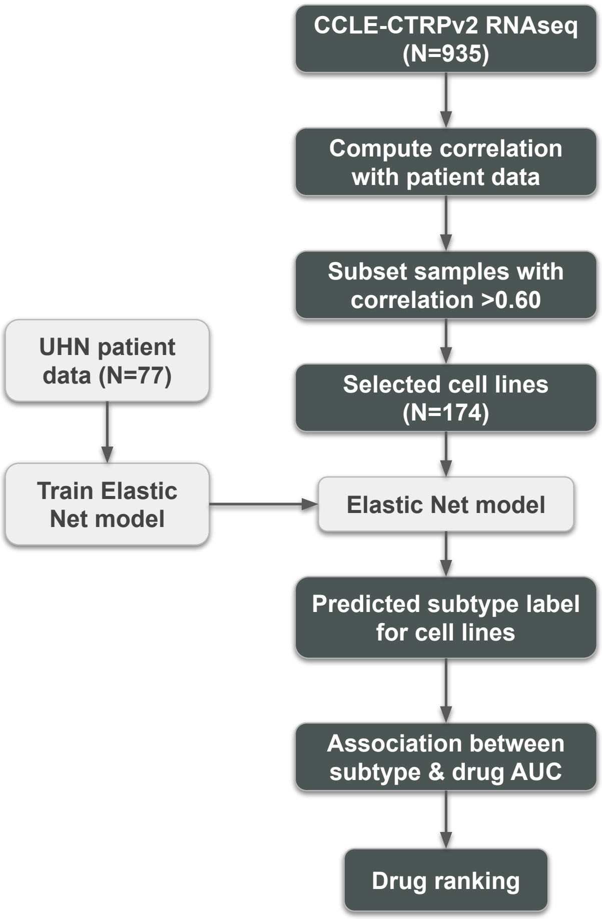

Supplementary Figure 23: Pharmacogenomic pipeline for drug ranking. CCLE-CTRPv2 pharmacogenomic data was used for the

The data was downloaded and processed using the R PharmacoGx package version 3.8 [17, 18]. The CCLE-CTRPv2 dataset contains gene expression data from 935 cell lines encompassing 25 different tissue types. However AML cell lines constitute only a small amount (< 30) of all cell lines. Furthermore not all of these cell lines have been tested

for a large number of drugs. Due to these limitations we decided to include all those cell lines in the analysis which have a high correlation with the UHN dataset. Figure 24 shows a histogram of correlations between CCLE-CTRPv2 cell lines and a centroid of the UHN dataset. We selected only cell lines with a correlation coefficient > 0.60 for further analysis (174 cell lines in total). Notably all cell lines with high correlation to the patient cohort are haematopoietic cell lines. Given the unique nature of blood cancers, it is not surprising that cell lines belonging to solid tumors do not show a strong correlation to AML patient data.

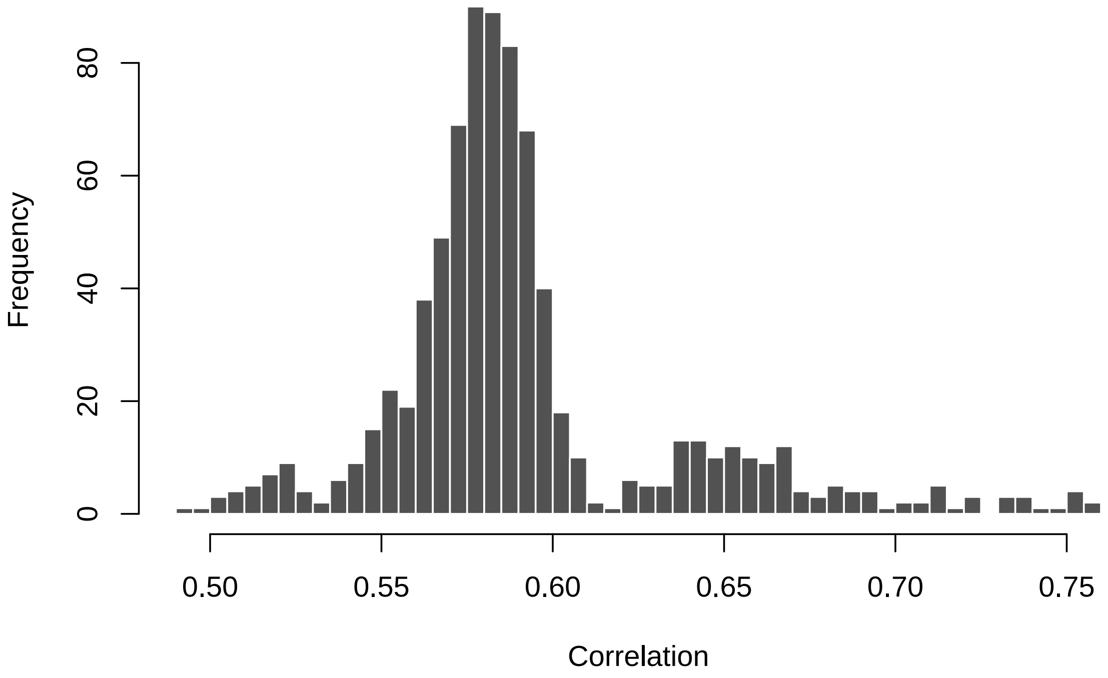

Supplementary Figure 24: Cell lines to patient correlation. Histogram shows distribution of Pearson correlation coefficient between CCLE-CTRPv2 cell lines and UHN patient data.

These selected cell lines were classified into primitive and committed subtype using a Elastic Net machine learning model. For this purpose first we trained the model on the UHN dataset to predict the primitive and committed subtype from gene expression data (figure 25). This model was directly applied to the cell line gene expression data.

Next we computed the association between predicted cell line labels and area under curve (AUC) values for each drug using the nonparametric measure concordance index (CI). Supplementary data file X sheet-2 shows results of this association analysis. We used these CI values to rank the drugs from potentially most to least effective on the primitive subtype. The ranked list of compounds contained 448 compounds which we further narrowed down as shown in figure 26. First we created a subset of the drug list by selecting only FDA approved drugs. Next we removed those compounds whose target genes are not differentially expressed between primitive and committed subtype. Evidently the resulting list was enriched with kinome class of drugs, therefore we decided to focus on this class of drugs only. We used the p-value cutoff of 0.1 to further narrow down the drugs. From the resulting list, five compounds were selected based on their availability. These compounds are *Ruxolitinib, Sunitinib, Sorafenib, Quizartinib* and *Imatinib*. We also included *Dasatinib* as a negative control. In total six drugs were selected for ex vivo drug screening.

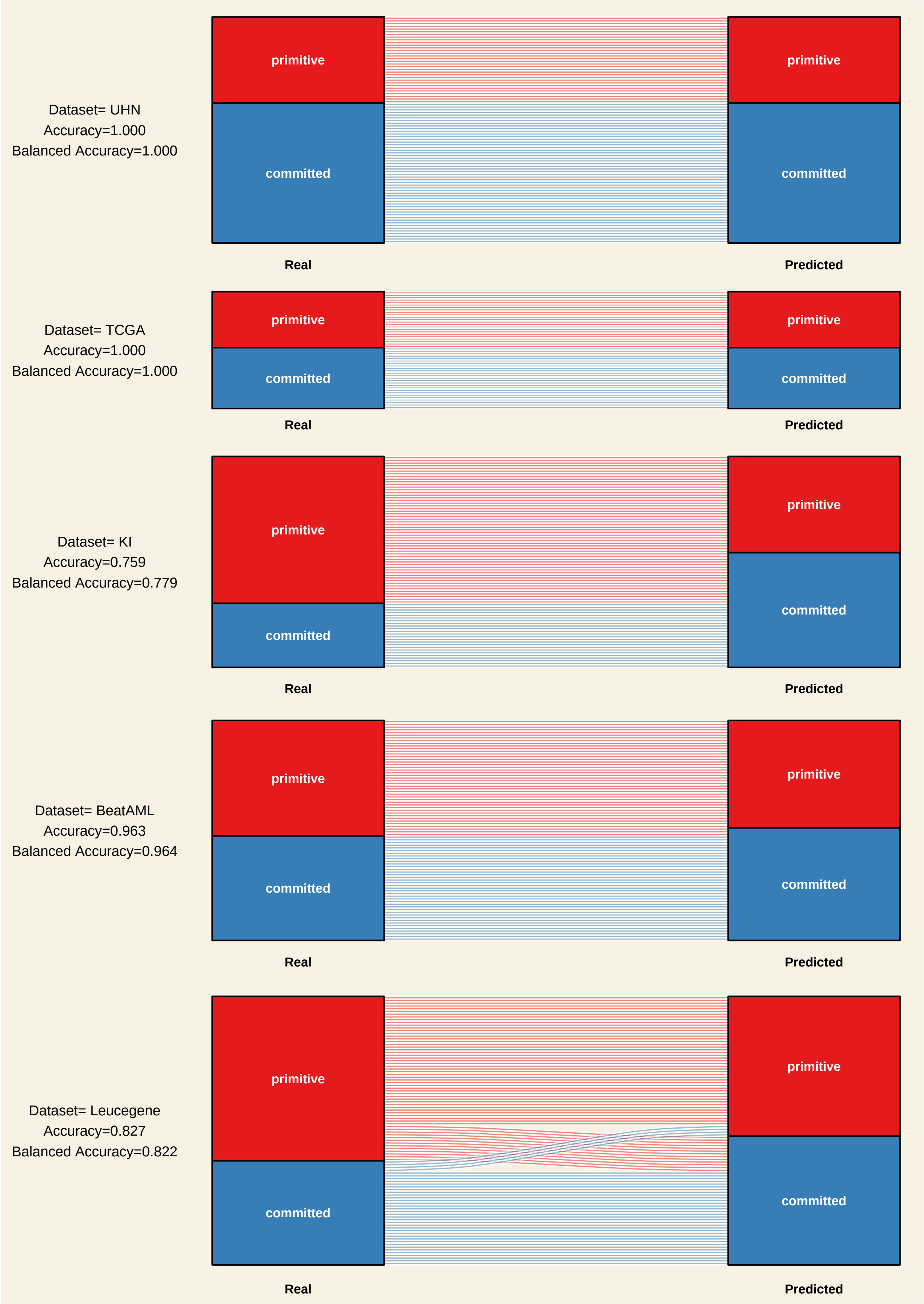

Supplementary Figure 25: Training and validation of supervised multivariate machine learning model on different AML patient cohorts. For each dataset, the left side represents samples with primitive and committed labels and the predicted labels are represented on the right side.

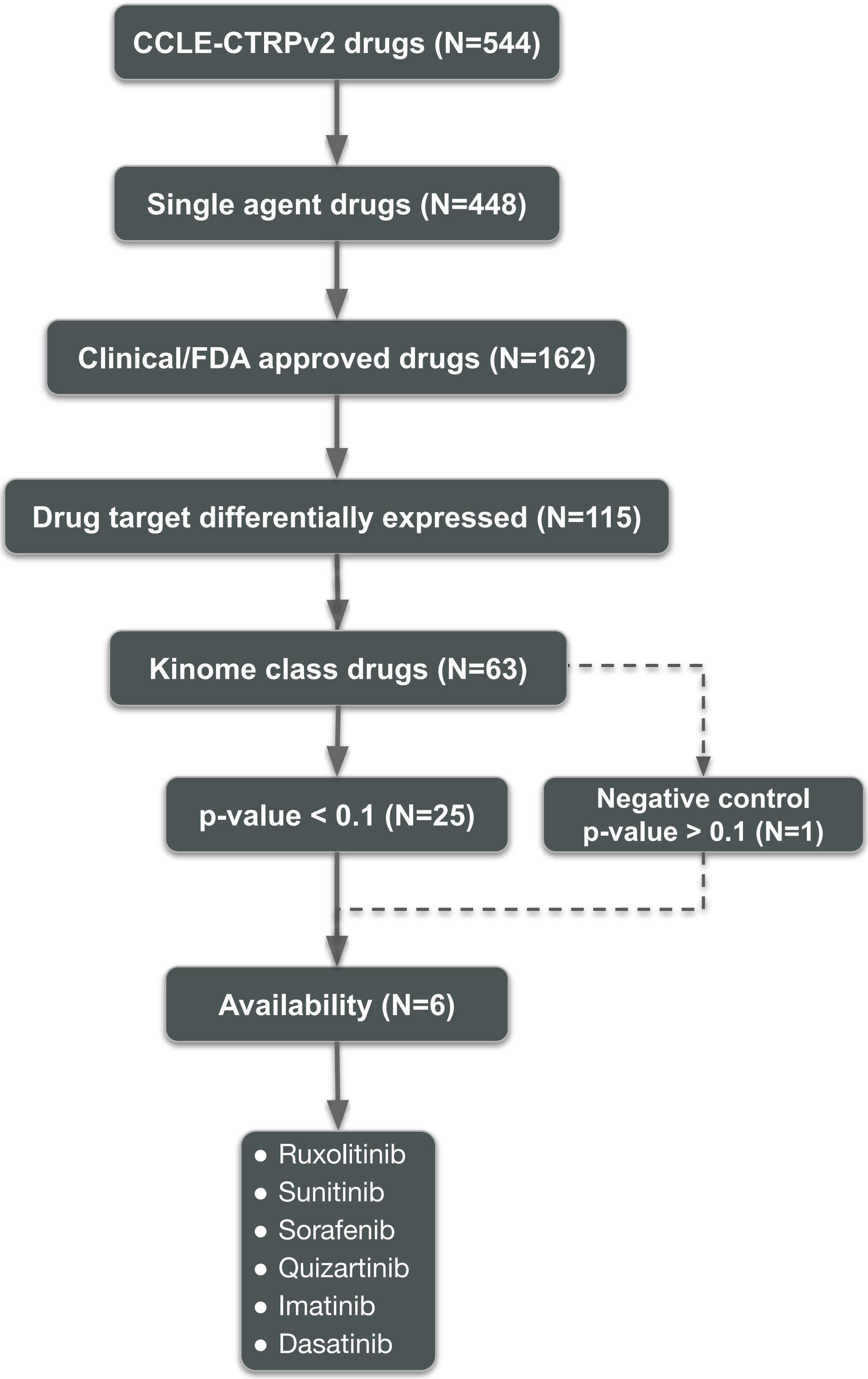

Supplementary Figure 26: Drug prioritization pipeline. For drug prioritization we used the CCLE-CTRPv2 dataset using the

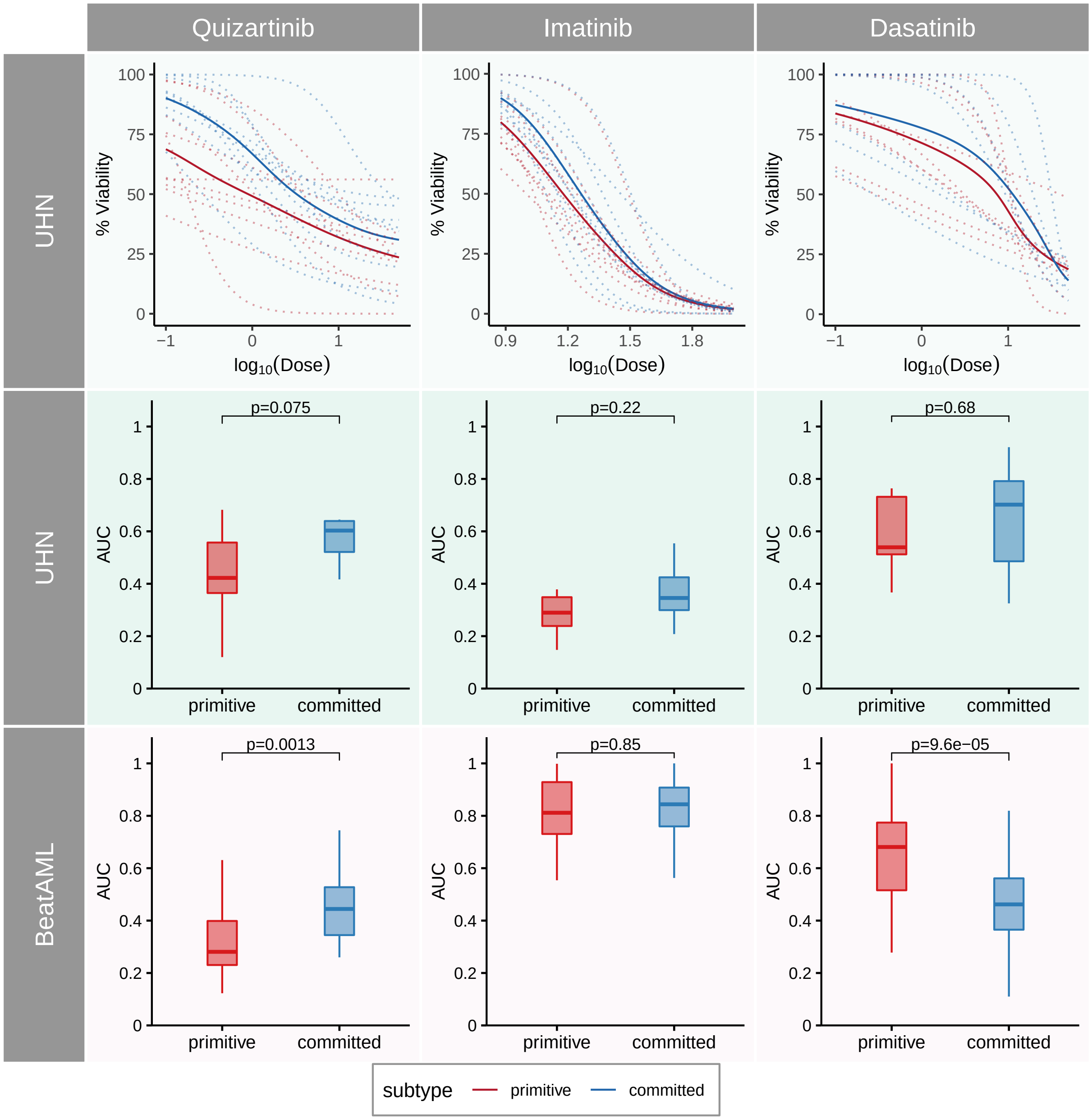

Supplementary Figure 27: Activity of three different kinase inhibitors Quizartinib, Imatinib and Dasatinib in two different *ex vivo* screenings. Top panel shows drug-dose response curves for patients and second panel shows AUC values for *ex vivo* screening in UHN patient cohort. The third panel shows response of the drug in BeatAML patient cohort. All p-values are for two-sided Wilcoxon rank-sum test. The primitive subtype is more sensitive towards Quizartinib in the UHN and BeatAML cohort. For the UHN cohort, $n = 10$ in primitive and $n = 10$ in committed subtype. For the BeatAML cohort, $n = 33$ in primitive and $n = 29$ in committed subtype. In the boxplots, middle line indicates median. The lower and upper hinges of the boxes correspond to the 25th and 75th percentiles. The whiskers represent 1.5 x IQR from the hinge (where IQR is the inter-quartile range). Data beyond the end of the whiskers are outlying points that are plotted individually.

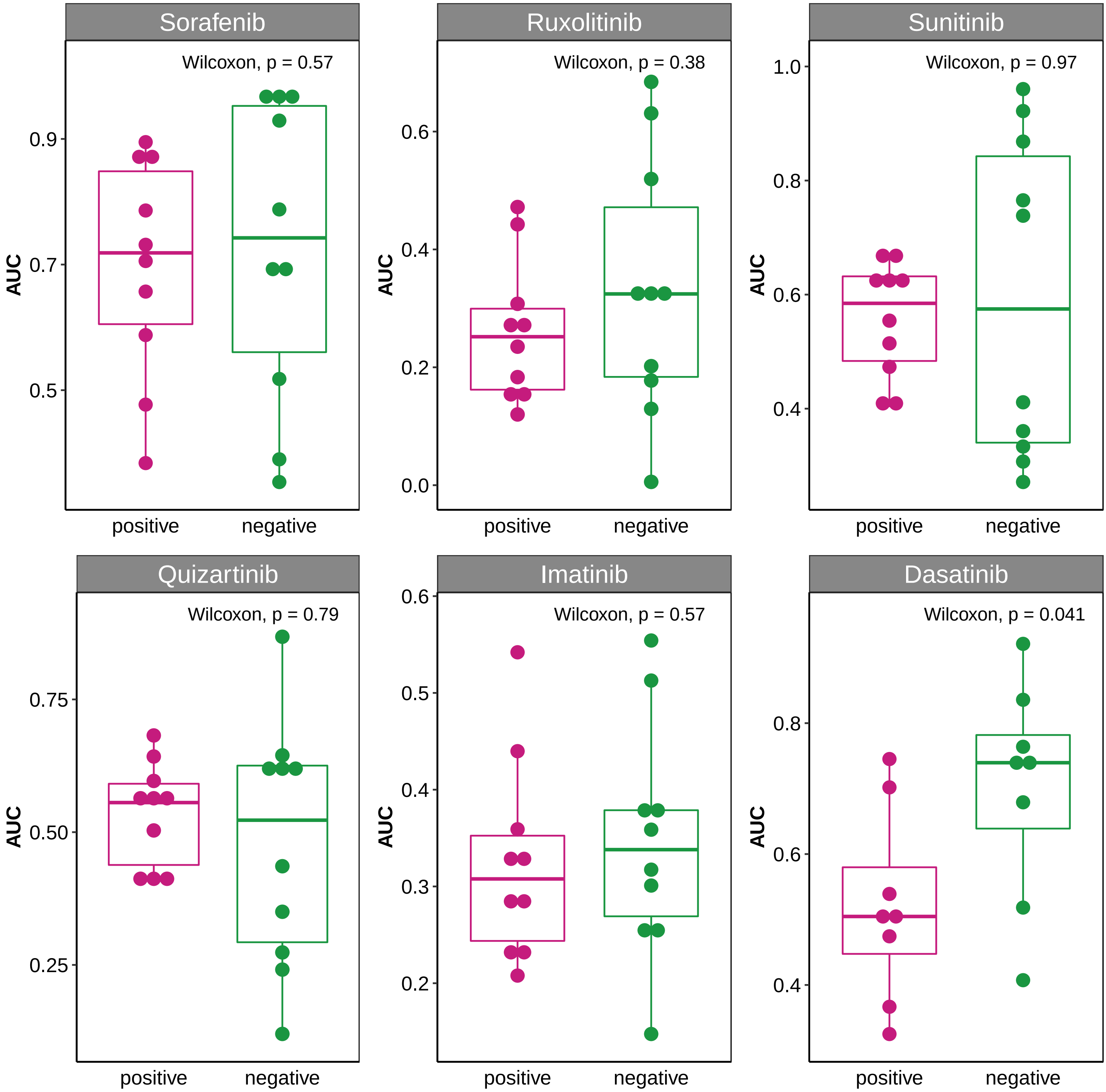

Supplementary Figure 28: FLT3-ITD status and the response of Sorafenib, Sunitinib, Quizartinib, Ruxolitinib, Imatinib and Dasatinib in the UHN dataset. Boxplots show AUC values for *ex vivo* screening in the UHN cohort ($n = 10$ in FLT3-ITD positive and $n = 10$ in FLT3-ITD negative group). In the boxplots, middle line indicates median. The lower and upper hinges of the boxes correspond to the 25th and 75th percentiles. The whiskers represent 1.5 x IQR from the hinge (where IQR is the inter-quartile range). Data beyond the end of the whiskers are outlying points that are plotted individually. Two-sided Wilcoxon rank-sum test was used for pairwise comparison and p-vales are indicated in the plot.

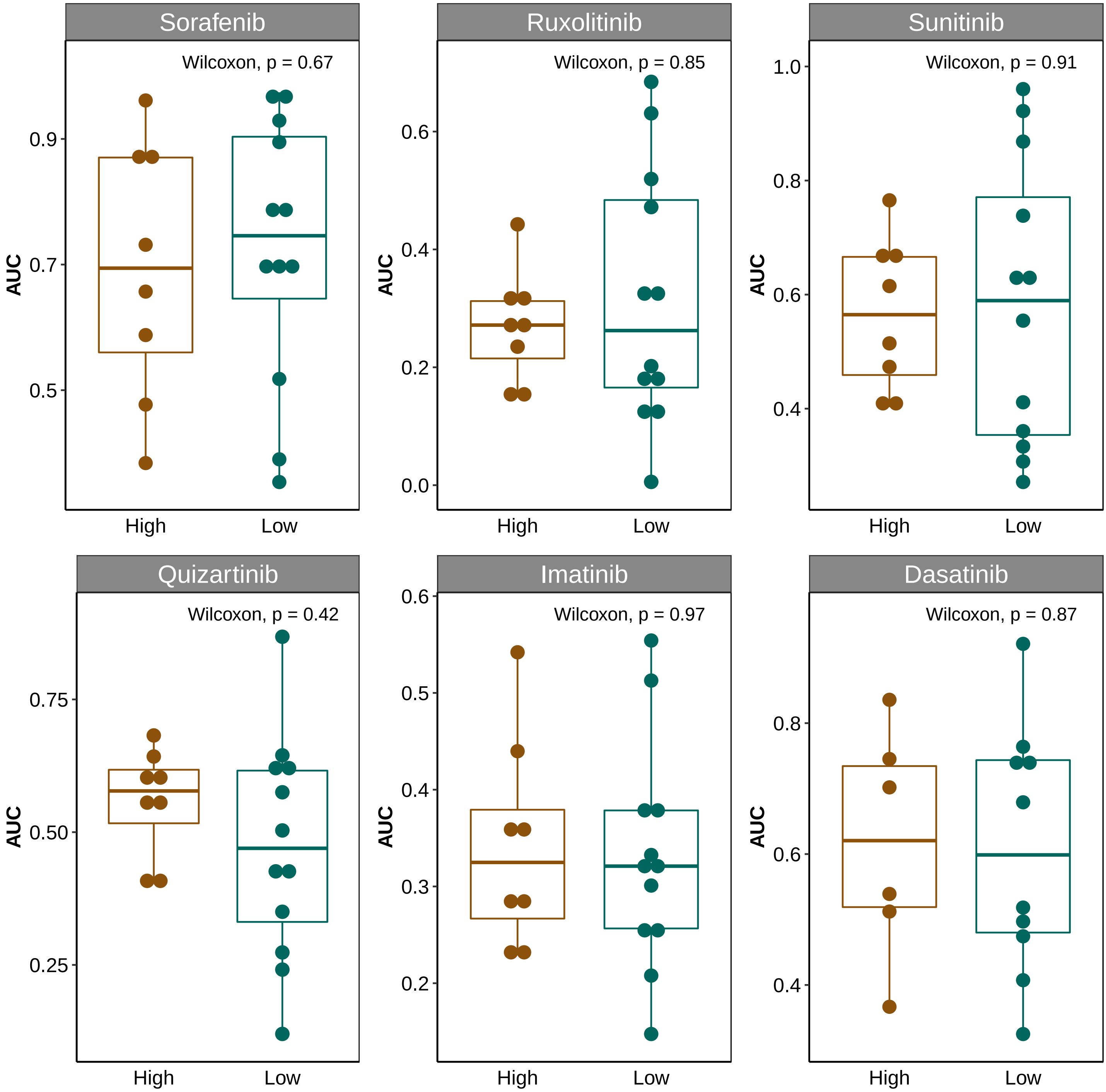

Supplementary Figure 29: Leukemia stem cell score (LSC) and the response of Sorafenib, Sunitinib, Quizartinib, Ruxolitinib, Imatinib and Dasatinib in the UHN dataset. Boxplots show AUC values for *ex vivo* screening in the UHN cohort ($n = 8$ in LSC high and $n = 12$ in LSC low group). In the boxplots, middle line indicates median. The lower and upper hinges of the boxes correspond to the 25th and 75th percentiles. The whiskers represent 1.5 x IQR from the hinge (where IQR is the inter-quartile range). Data beyond the end of the whiskers are outlying points that are plotted individually. Two-sided Wilcoxon rank-sum test was used for pairwise comparison and p-vales are indicated in the plot.

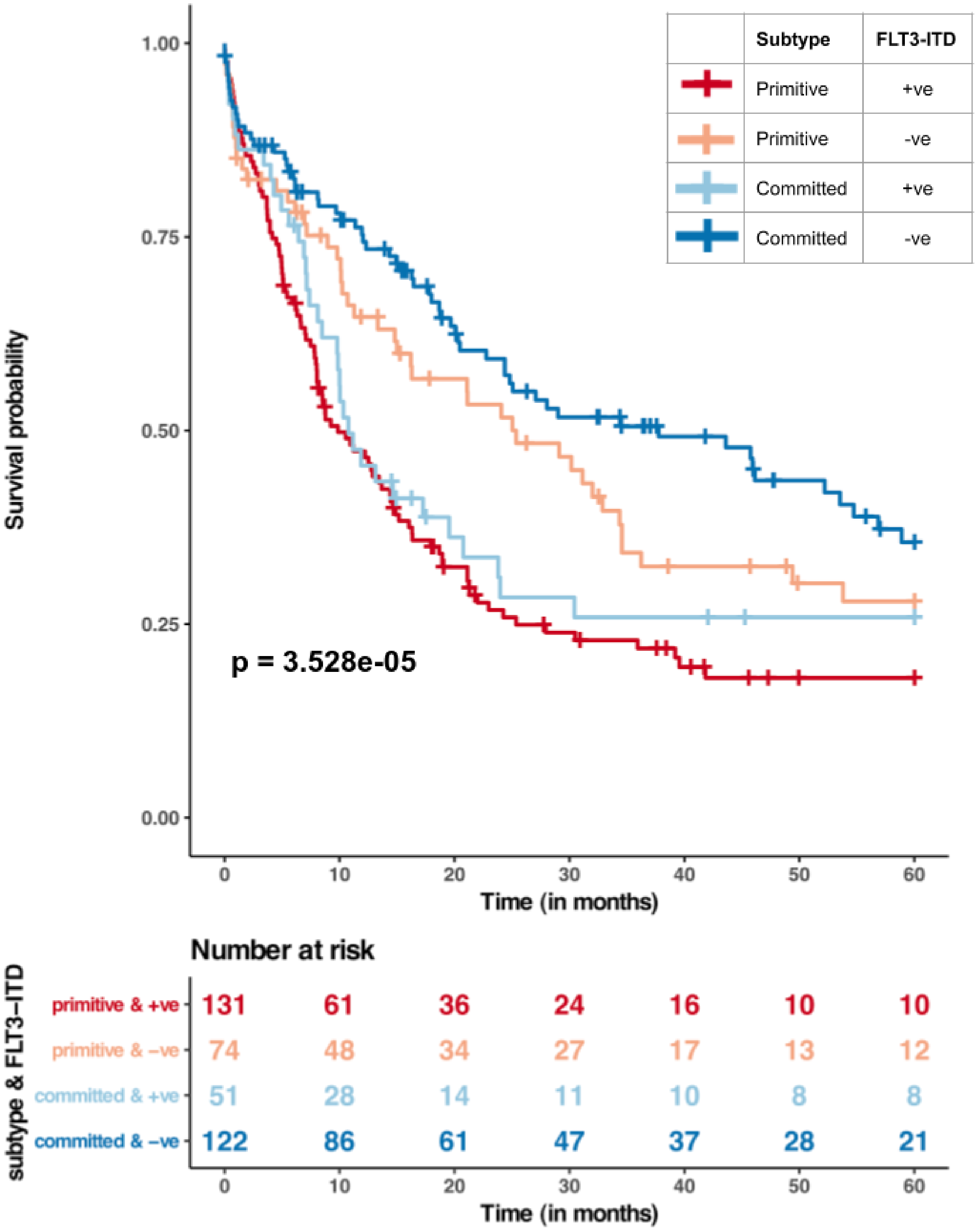

Supplementary Figure 30: Kaplan-Meier plot of overall survival for primitive and committed subtypes and FLT3-ITD status. Log-rank (Mantel-Cox) test p-values is indicated in the plot.

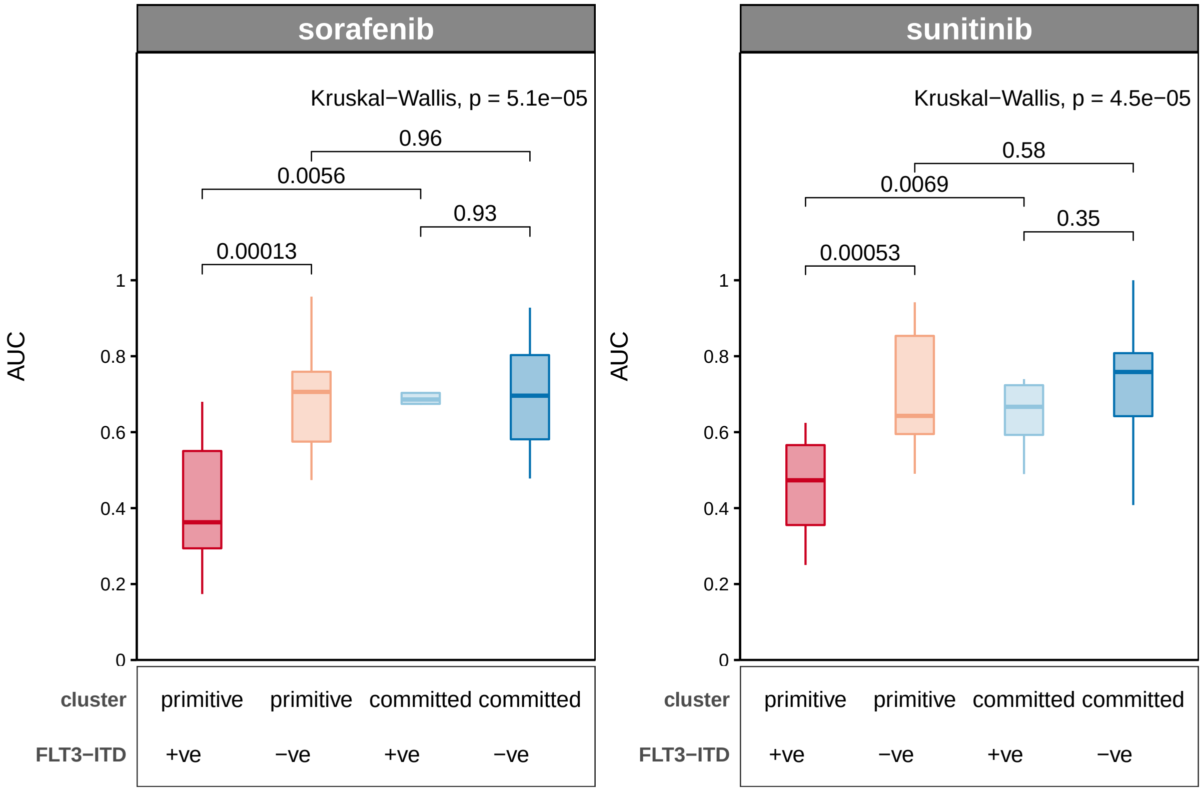

Supplementary Figure 31: Stratification of drug sensitivity by subtype and FLT3-ITD status for sorafenib and sunitinib in the BeatAML cohort. Ex vivo drug screening was performed on *n = 62* AML patient samples (primitive FLT3-ITD positive*n = 25*; primitive FLT3-ITD negative *n = 18*; committed FLT3-ITD positive*n = 7*; committed FLT3-ITD negative *n = 32*). Two-sided Wilcoxon rank-sum test was used for pairwise comparison and p-value is shown on top of the boxplots. Non-parametric Kruskal-Wallis test was used for comparing multiple groups. In the boxplots, middle line indicates median. The lower and upper hinges of the boxes correspond to the 25th and 75th percentiles. The whiskers represent 1.5 x IQR from the hinge (where IQR is the inter-quartile range). Data beyond the end of the whiskers are outlying points that are plotted individually.

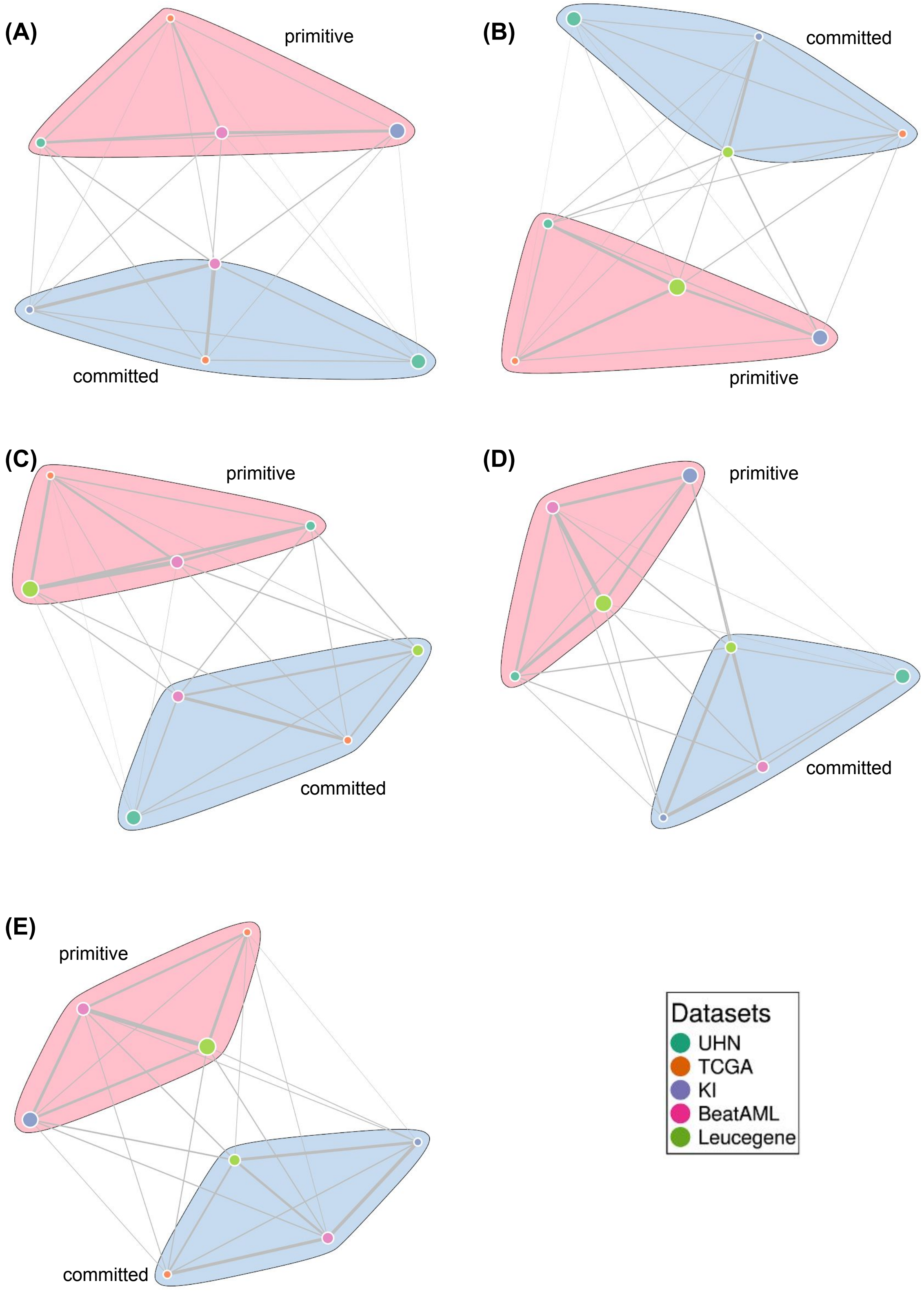

Supplementary Figure 32: Leave one dataset out cross-validation meta clustering of the data. (A), (B), (C), (D) and (E) represent clustering results while leaving Leucegene, BeatAML, KI, TCGA and UHN dataset out respectively. The size of the nodes is proportional to the number of patients in the cluster and nodes are colored according to the dataset. The edge width is proportional to the correlation between clusters.

Supplementary Table 2: Subtypes vs. Age contingency table. Pearson's chi-square test p-values are indicated on the top of the contingency table and FDR is indicated on the top of the table.

Age (FDR=7.14e-01)

	UHN (p=7.09e-01)		TCGA (p=7.43e-01)		KI (p=1.26e-01)		BeatAML (p=6.03e-01)		Leucegene (p=4.88e-01)		Total (p=4.97e-01)	
	<60	>=60	<60	>=60	<60	>=60	<60	>=60	<60	>=60	<60	>=60
primitive	18	10	14	9	14	41	15	25	35	24	96	109
committed	28	21	13	12	11	13	17	20	19	19	88	85

Supplementary Table 3: Subtypes vs. WBC contingency table. Pearson's chi-square test p-values are indicated on the top of the contingency table and FDR is indicated on the top of the table.

WBC (FDR=1.48e-01)

	UHN (p=3.63e-02)		TCGA (p=5.78e-01)		KI (p=4.41e-01)		BeatAML (p=6.69e-01)		Leucegene (p=2.79e-02)		Total (p=5.63e-02)	
	High	Low	High	Low	High	Low	High	Low	High	Low	High	Low
primitive	18	10	11	12	16	21	16	18	38	21	99	82
committed	18	31	15	10	10	7	12	19	15	23	70	90

Supplementary Table 4: Subtypes vs. karyotype contingency table. Pearson's chi-square test p-values are indicated on the top of the contingency table and FDR is indicated on the top of the table.

karyotype (FDR=3.24e-01)

	TCGA (p=1)		KI (p=3.08e-01)		BeatAML (p=6.53e-01)		Leucegene (p=4.28e-01)		Total (p=1.7e-01)	
	complex	normal	complex	normal	complex	normal	complex	normal	complex	normal
primitive	1	20	11	42	8	19	11	48	31	129
committed	2	23	2	22	7	26	4	34	15	105

Supplementary Table 5: Subtypes vs. Sex contingency table. Pearson's chi-square test p-values are indicated on the top of the contingency table and FDR is indicated on the top of the table.

Sex (FDR=4.35e-02)

	UHN (p=8.74e-01)		TCGA (p=2.36e-01)		KI (p=3.91e-01)		BeatAML (p=2.26e-01)		Leucegene (p=1.48e-01)		Total (p=1.24e-02)	
	F	M	F	M	F	M	F	M	F	M	F	M
primitive	19	9	15	8	37	18	27	13	38	21	136	69
committed	31	18	11	14	13	11	19	18	18	20	92	81

Supplementary Table 6: Subtypes vs. transplant contingency table. Pearson's chi-square test p-values are indicated on the top of the contingency table and FDR is indicated on the top of the table.

transplant (FDR=8.04e-01)

	UHN (p=5.07e-01)		TCGA (p=7.99e-01)		KI		BeatAML		Leucegene		Total (p=6.44e-01)	
	No	Yes	No	Yes	No	Yes	No	Yes	No	Yes	No	Yes
primitive	19	9	12	11	55	0	40	0	43	0	169	20
committed	38	11	15	10	23	0	36	0	32	0	144	21

Supplementary Table 7: Subtypes vs. FLT3-ITD contingency table. Pearson's chi-square test p-values are indicated on the top of the contingency table and FDR is indicated on the top of the table.

FLT3-ITD (FDR=1.06e-09)

	UHN (p=1.36e-04)		TCGA (p=4.48e-02)		KI (p=8.65e-01)		BeatAML (p=2.67e-04)		Leucegene (p=1.46e-04)		Total (p=5.04e-11)	
	mutation	wild	mutation	wild	mutation	wild	mutation	wild	mutation	wild	mutation	wild
primitive	22	6	13	10	28	27	25	15	43	16	131	74
committed	15	34	6	19	11	13	7	30	12	26	51	122

Supplementary Table 8: Subtypes vs. NRAS contingency table. Pearson's chi-square test p-values are indicated on the top of the contingency table and FDR is indicated on the top of the table.

NRAS (FDR=2.86e-02)

	TCGA (p=1)		KI (p=4.1e-02)		BeatAML (p=9.01e-02)		Leucegene (p=2.79e-01)		Total (p=2.72e-03)	
	mutation	wild	mutation	wild	mutation	wild	mutation	wild	mutation	wild
primitive	2	21	2	53	1	39	4	55	9	168
committed	3	22	5	19	6	31	6	32	20	104

Supplementary Table 9: Subtypes vs. DNMT3A contingency table. Pearson's chi-square test p-values are indicated on the top of the contingency table and FDR is indicated on the top of the table.

DNMT3A (FDR=3.85e-02)

	TCGA (p=7.63e-01)		KI (p=3.88e-03)		BeatAML (p=9.17e-02)		Leucegene (p=1)		Total (p=5.49e-03)	
	mutation	wild	mutation	wild	mutation	wild	mutation	wild	mutation	wild
primitive	10	13	14	41	14	26	32	27	70	107
committed	13	12	15	9	21	16	21	17	70	54

Supplementary Table 10: Subtypes vs. KRAS contingency table. Pearson's chi-square test p-values are indicated on the top of the contingency table and FDR is indicated on the top of the table.

KRAS (FDR=4.35e-02)

	TCGA (p=2.63e-01)		KI (p=1.65e-01)		BeatAML (p=9.69e-01)		Leucegene (p=6.96e-01)		Total (p=9.12e-03)	
	mutation	wild	mutation	wild	mutation	wild	mutation	wild	mutation	wild
primitive	0	23	0	55	0	40	1	58	1	176
committed	3	22	2	22	1	36	2	36	8	116

Supplementary Table 11: Subtypes vs. FLT3-TKD contingency table. Pearson's chi-square test p-values are indicated on the top of the contingency table and FDR is indicated on the top of the table.

FLT3-TKD (FDR=4.35e-02)

	UHN (p=3.08e-01)		TCGA (p=1.95e-02)		KI (p=1)		BeatAML (p=1)		Leucegene (p=7.7e-02)		Total (p=1.24e-02)	
	mutation	wild	mutation	wild	mutation	wild	mutation	wild	mutation	wild	mutation	wild
primitive	0	28	0	23	4	51	9	31	4	55	17	188
committed	4	45	7	18	2	22	9	28	8	30	30	143

Supplementary Table 12: Subtypes vs. PTPN11 contingency table. Pearson's chi-square test p-values are indicated on the top of the contingency table and FDR is indicated on the top of the table.

PTPN11 (FDR=2.27e-01)

	TCGA (p=9.22e-01)		KI (p=9.33e-02)		BeatAML (p=6.01e-03)		Leucegene (p=8.03e-01)		Total (p=9.74e-02)	
	mutation	wild	mutation	wild	mutation	wild	mutation	wild	mutation	wild
primitive	3	20	3	52	1	39	15	44	22	155
committed	2	23	5	19	10	27	8	30	25	99

Supplementary Table 13: Subtypes vs. WT1 contingency table. Pearson's chi-square test p-values are indicated on the top of the contingency table and FDR is indicated on the top of the table.

WT1 (FDR=2.29e-01)

	TCGA (p=9.79e-02)		KI (p=5.99e-01)		BeatAML (p=1)		Leucegene (p=6.32e-01)		Total (p=1.09e-01)	
	mutation	wild	mutation	wild	mutation	wild	mutation	wild	mutation	wild
primitive	4	19	3	52	4	36	6	53	17	160
committed	0	25	0	24	3	34	2	36	5	119

Supplementary Table 14: Subtypes vs. IDH2 contingency table. Pearson's chi-square test p-values are indicated on the top of the contingency table and FDR is indicated on the top of the table.

IDH2 (FDR=3.5e-01)

	TCGA (p=1)		KI (p=4.67e-01)		BeatAML (p=1)		Leucegene (p=6.17e-01)		Total (p=2e-01)	
	mutation	wild	mutation	wild	mutation	wild	mutation	wild	mutation	wild
primitive	2	21	15	40	7	33	18	41	42	135
committed	2	23	4	20	6	31	9	29	21	103

Supplementary Table 15: Subtypes vs. CEBPA contingency table. Pearson's chi-square test p-values are indicated on the top of the contingency table and FDR is indicated on the top of the table.

CEBPA (FDR=8.04e-01)

	TCGA (p=1)		KI (p=8.57e-01)		BeatAML (p=1)		Leucegene (p=1)		Total (p=6.51e-01)	
	mutation	wild	mutation	wild	mutation	wild	mutation	wild	mutation	wild
primitive	1	22	7	48	2	38	2	57	12	165
committed	1	24	2	22	2	35	1	37	6	118

Supplementary Table 16: Subtypes vs. ASXL1 contingency table. Pearson's chi-square test p-values are indicated on the top of the contingency table and FDR is indicated on the top of the table.

ASXL1 (FDR=8.81e-01)

	TCGA		KI		BeatAML (p=1)		Leucegene (p=8.24e-01)		Total (p=7.56e-01)	
	mutation	wild	mutation	wild	mutation	wild	mutation	wild	mutation	wild
primitive	0	23	0	55	1	39	0	59	1	176
committed	0	25	0	24	1	36	1	37	2	122

Supplementary Table 17: Subtypes vs. RUNX1 contingency table. Pearson's chi-square test p-values are indicated on the top of the contingency table and FDR is indicated on the top of the table.

RUNX1 (FDR=1)

	TCGA		KI		BeatAML (p=9.69e-01)		Leucegene (p=1)		Total (p=1)	
	mutation	wild	mutation	wild	mutation	wild	mutation	wild	mutation	wild
primitive	0	23	0	55	0	40	1	58	1	176
committed	0	25	0	24	1	36	0	38	1	123

Supplementary References

[1] Genomic and epigenomic landscapes of adult de novo acute myeloid leukemia. *N. Engl. J. Med.* **368**, 2059–2074 (2013).

[2] Mer, A. S. *et al.* Expression levels of long non-coding RNAs are prognostic for AML outcome. *J. Hematol. Oncol.* **11**, 52 (2018).

[3] Tyner, J. W. *et al.* Functional genomic landscape of acute myeloid leukaemia. *Nature* **562**, 526–531 (2018).

[4] Marquis, M. *et al.* High expression of HMGA2 independently predicts poor clinical outcomes in acute myeloid leukemia (2018).

[5] Wilkerson, M. D. & Hayes, D. N. ConsensusClusterPlus: a class discovery tool with confidence assessments and item tracking. *Bioinformatics* **26**, 1572–1573 (2010).

[6] Planey, C. R. & Gevaert, O. CoINcIDE: A framework for discovery of patient subtypes across multiple datasets. *Genome Med.* **8**, 27 (2016).

[7] Qiao, W. *et al.* PERT: a method for expression deconvolution of human blood

samples from varied microenvironmental and developmental conditions. *PLoS Comput. Biol.* **8**, e1002838 (2012).

[8] Shlush, L. I. *et al.* Identification of pre-leukaemic haematopoietic stem cells in acute leukaemia. *Nature* **506**, 328–333 (2014).

[9] Notta, F. *et al.* Distinct routes of lineage development reshape the human blood hierarchy across ontogeny. *Science* **351** (2016).

[10] Passamonti, F. & Rumi, E. Clinical relevance of jak2 (v617f) mutant allele burden (2009).

[11] Lippert, E. *et al.* Concordance of assays designed for the quantification of jak2v617f: a multicenter study. *haematologica* **94**, 38–45 (2009).

[12] Anders, S. & Huber, W. Differential expression analysis for sequence count data. *Genome Biol.* **11**, R106 (2010).

[13] Love, M. I., Huber, W. & Anders, S. Moderated estimation of fold change and dispersion for rna-seq data with deseq2. *Genome biology* **15**, 550 (2014).

[14] Weber, L. M., Nowicka, M., Soneson, C. & Robinson, M. D. diffcyt: Differential discovery in high-dimensional cytometry via high-resolution clustering. *Commun Biol* **2**, 183 (2019).

[15] Van Gassen, S. *et al.* FlowSOM: Using self-organizing maps for visualization and interpretation of cytometry data. *Cytometry A* **87**, 636–645 (2015).

[16] Barretina, J. *et al.* The cancer cell line encyclopedia enables predictive modelling of anticancer drug sensitivity. *Nature* **483**, 603–607 (2012).

[17] Smirnov, P. *et al.* PharmacoGx: an R package for analysis of large pharmacogenomic datasets. *Bioinformatics* **32**, 1244–1246 (2016).

[18] PharmacoGx. https://doi.org/doi:10.18129/B9.bioc.PharmacoGx.
