# Supplementary material for: Biological and therapeutic implications of a unique subtype of NPM1 mutated AML
Source: Nat Commun. 2021 Feb 16;12:1054. doi: 10.1038/s41467-021-21233-0 (PMC7886883; doi:10.1038/s41467-021-21233-0)
Supplement: Supplementary file 2 — Description of Additional Supplementary Files [file 41467_2021_21233_MOESM2_ESM.pdf]

Description of Additional Supplementary Files

Supplementary Data 1: Differential gene expression analysis results

Supplementary Data 2: Results for subtype pathway analysis

Supplementary Data 3: Motif enrichment analysis

Supplementary Data 4: Ranking of drugs using cell line data

Supplementary Data 5: Drug-dose response curves for UHN ex vivo screening

Supplementary Data 6: List of antibodies used in CyToF analysis
