# Supplementary material for: Biological and therapeutic implications of a unique subtype of NPM1 mutated AML
Source: Nat Commun. 2021 Feb 16;12:1054. doi: 10.1038/s41467-021-21233-0 (PMC7886883; doi:10.1038/s41467-021-21233-0)

X110661 (subtype=primitive)

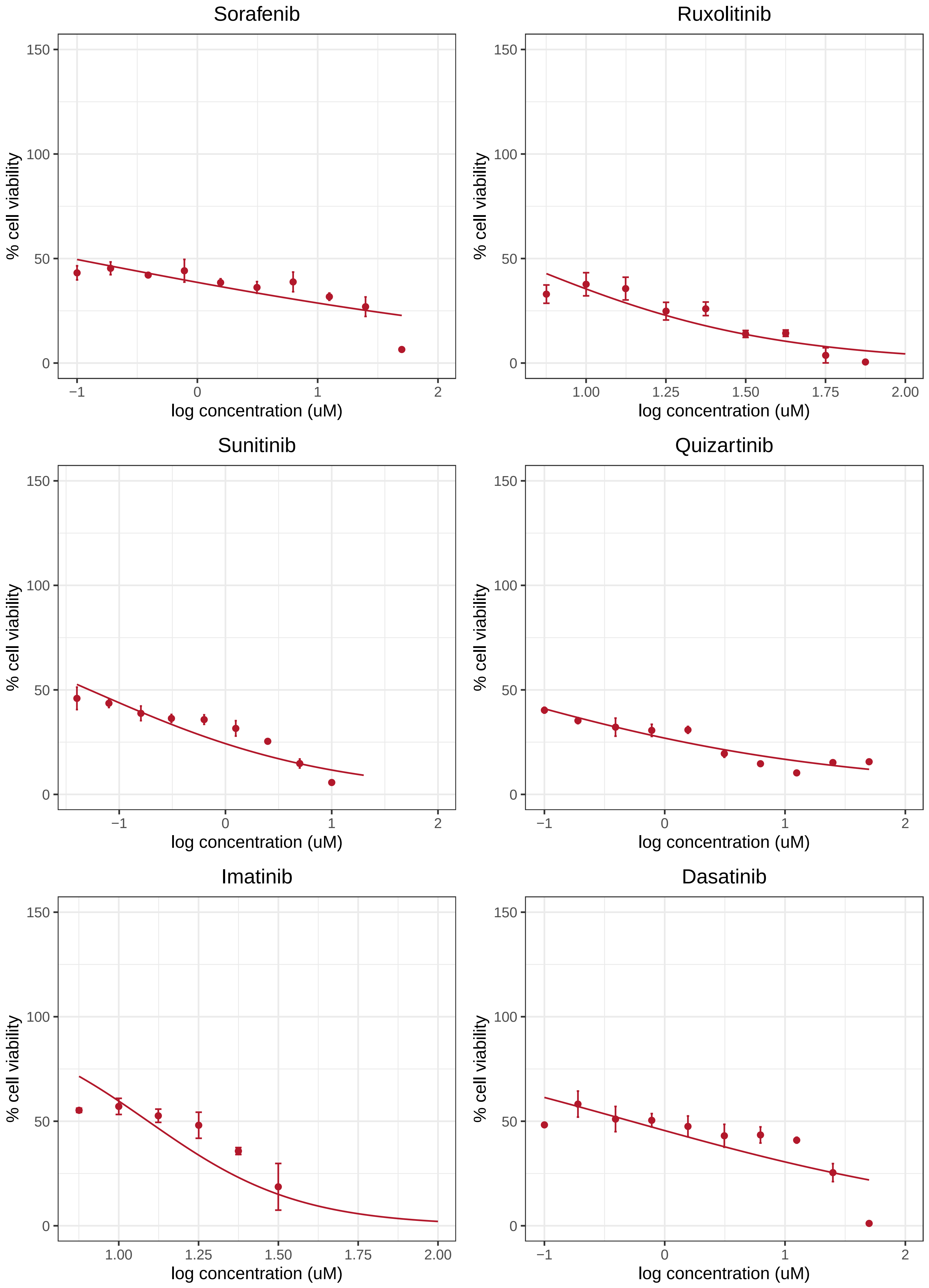

X130023 (subtype=primitive)

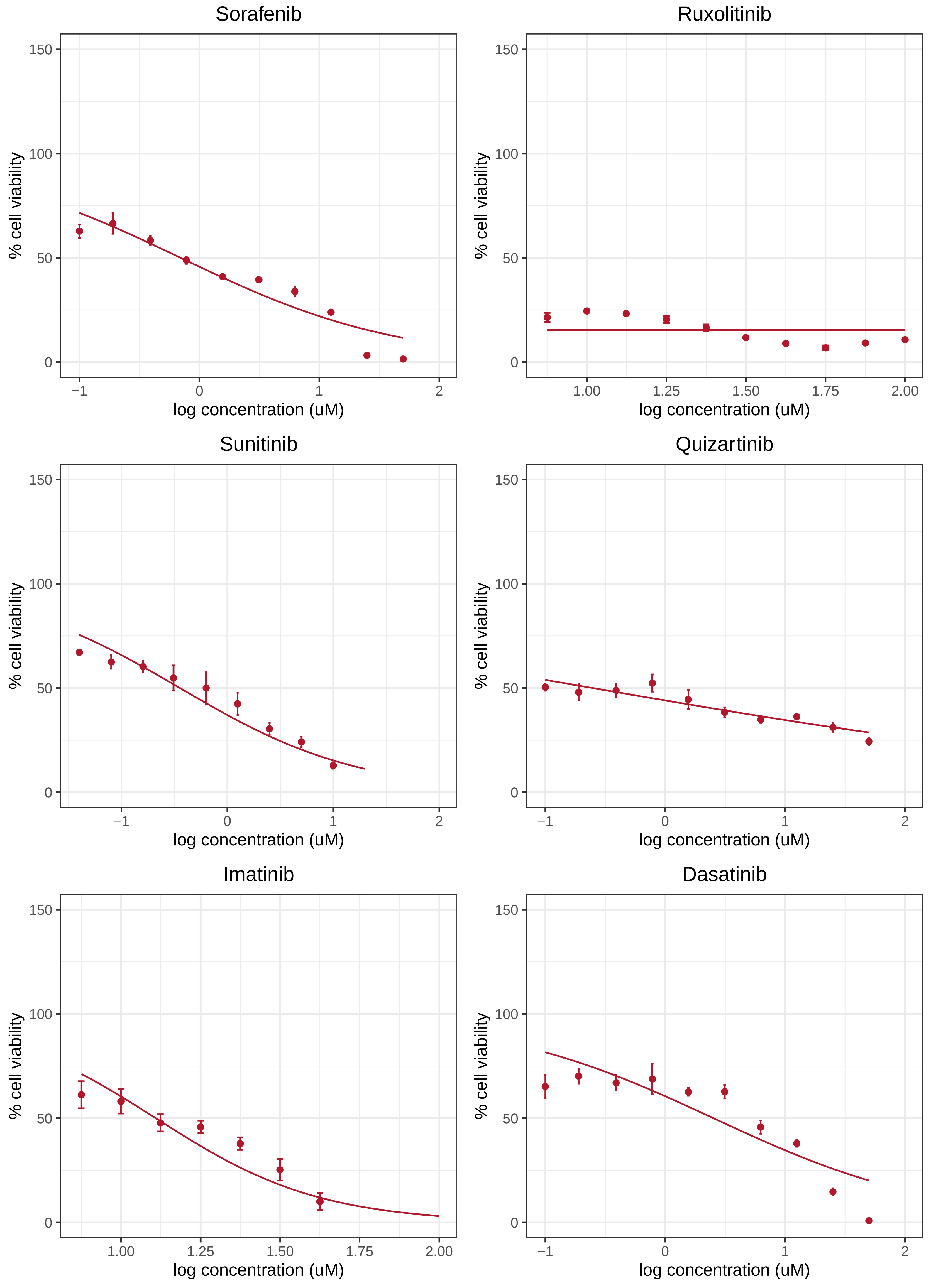

X2942752 (subtype=primitive)

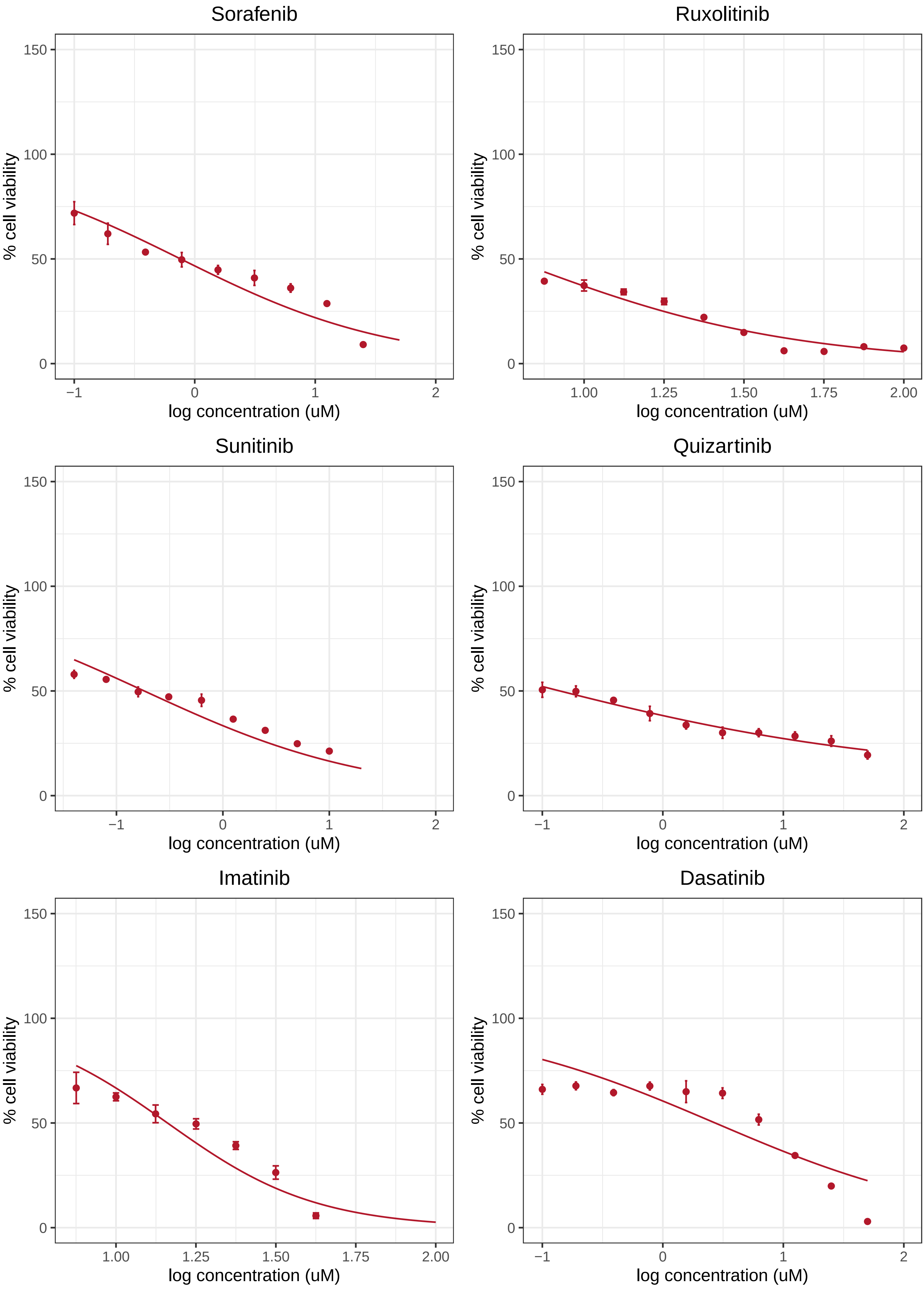

X80088 (subtype=primitive)

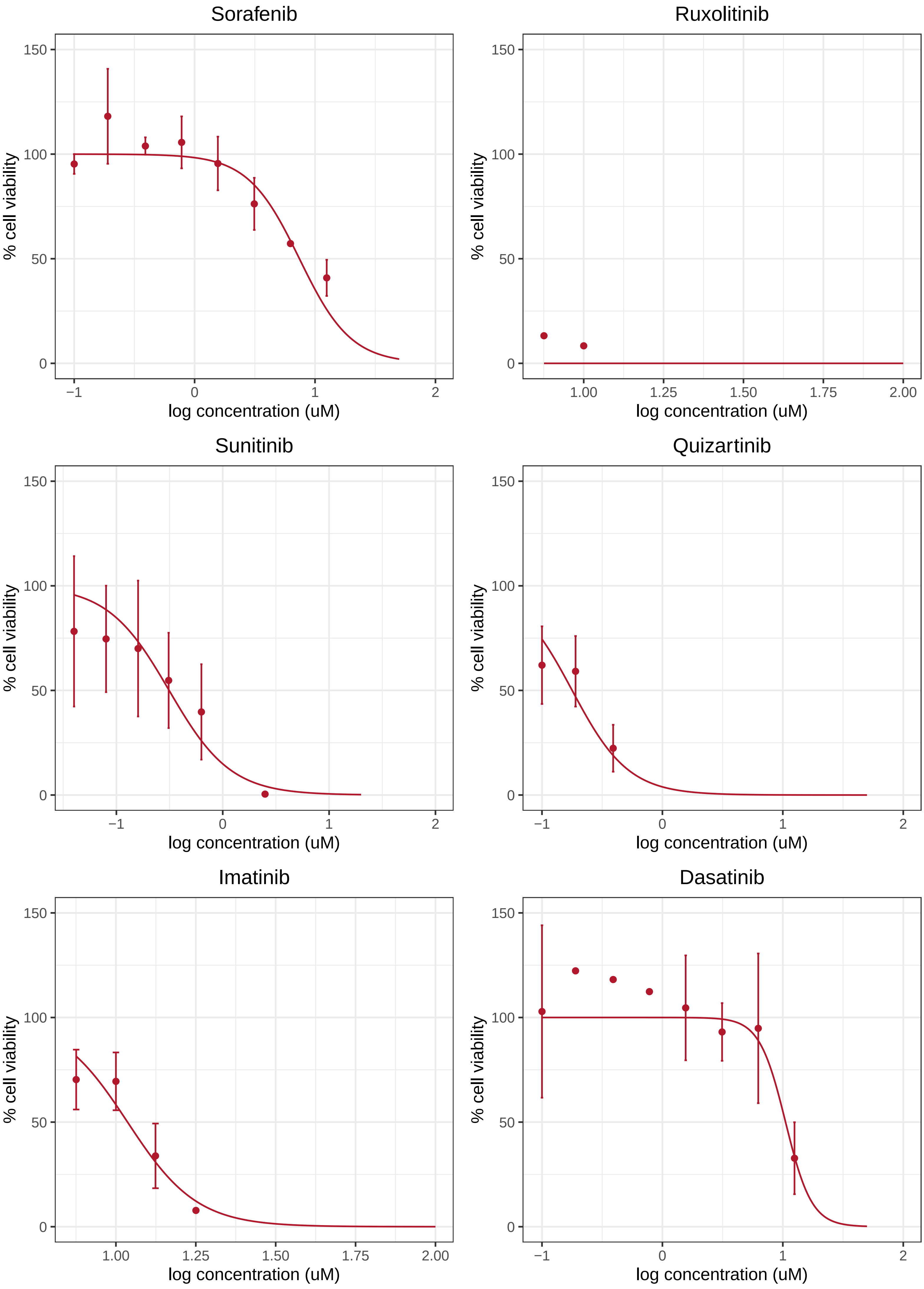

X140659 (subtype=primitive)

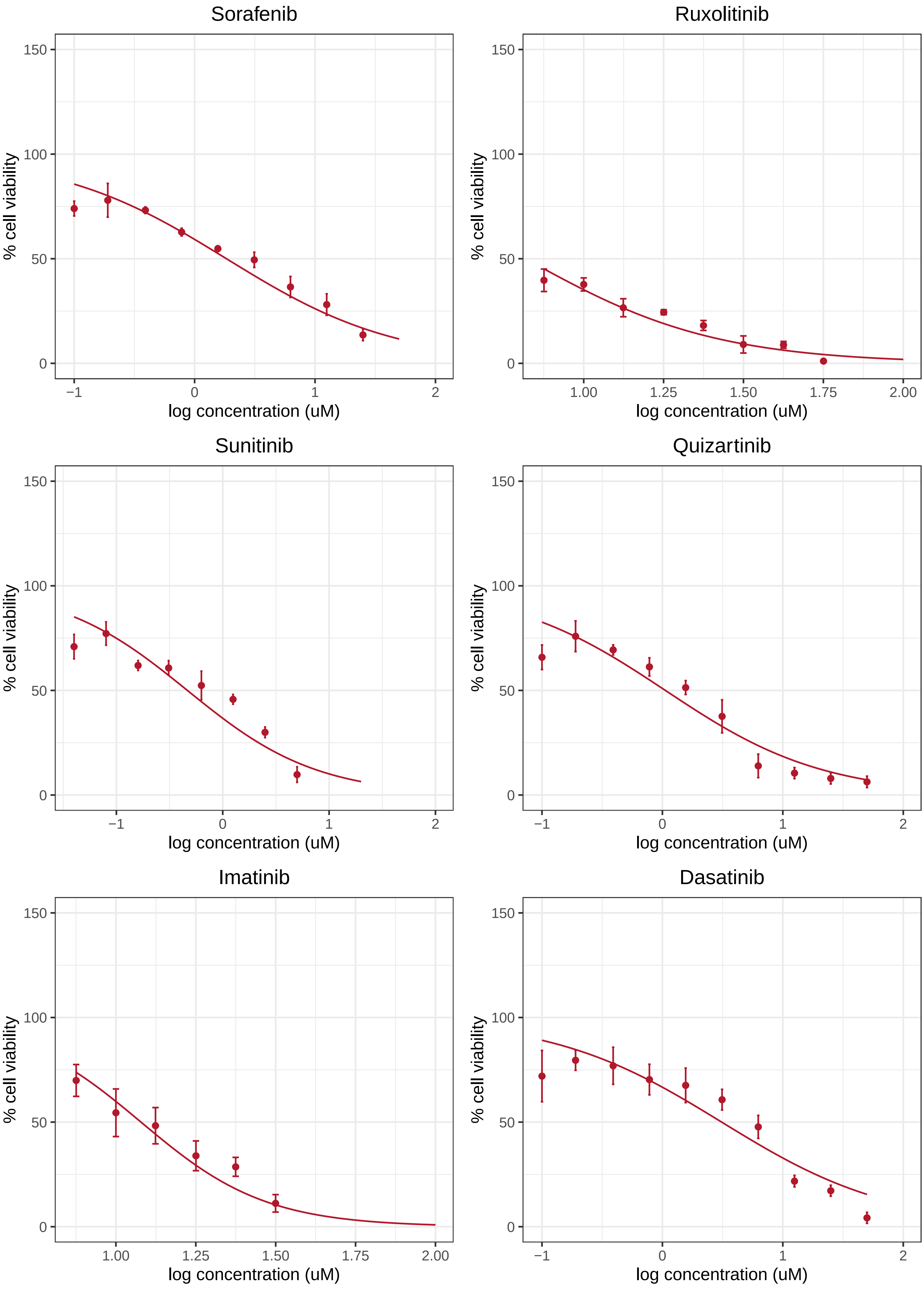

X120170 (subtype=primitive)

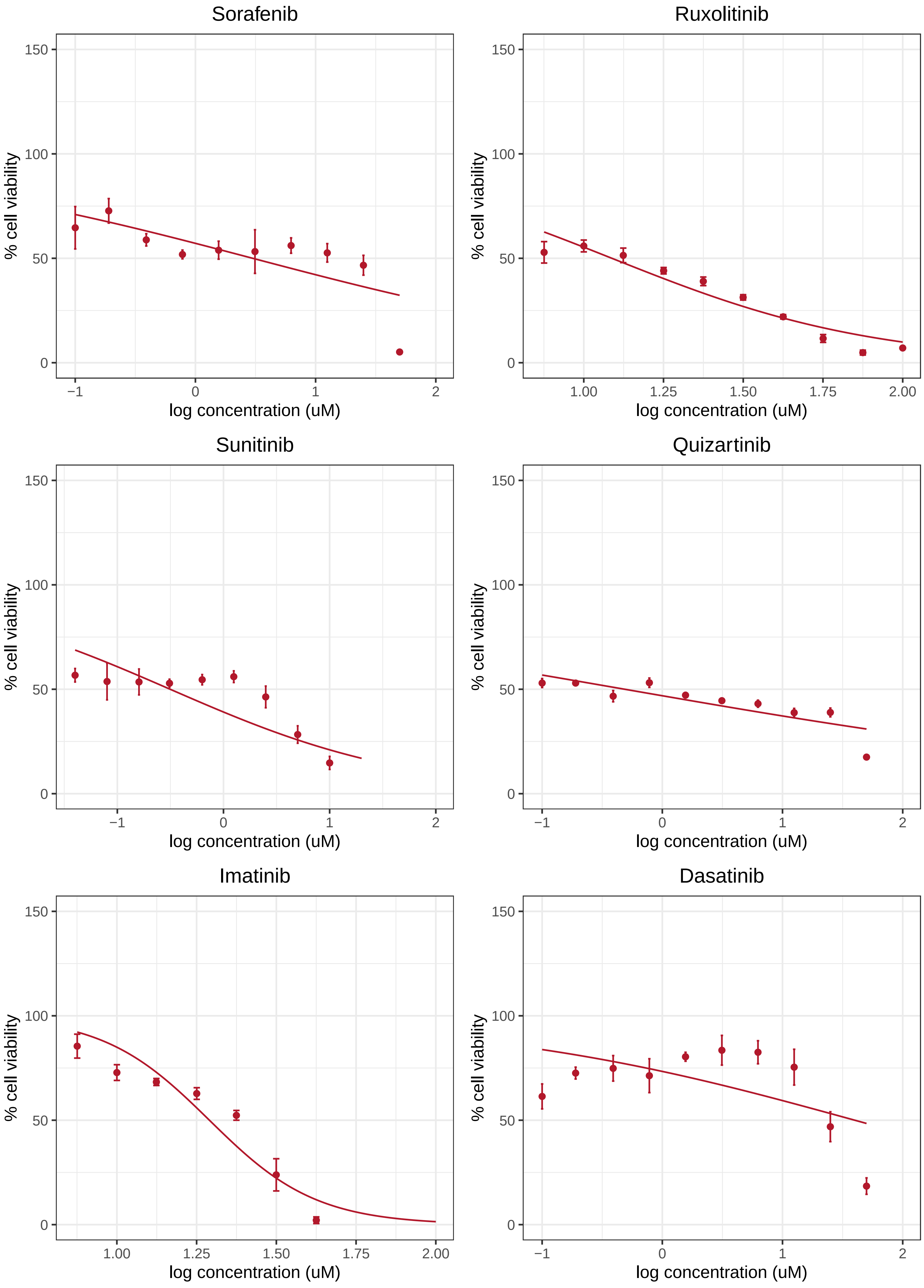

X120899 (subtype=primitive)

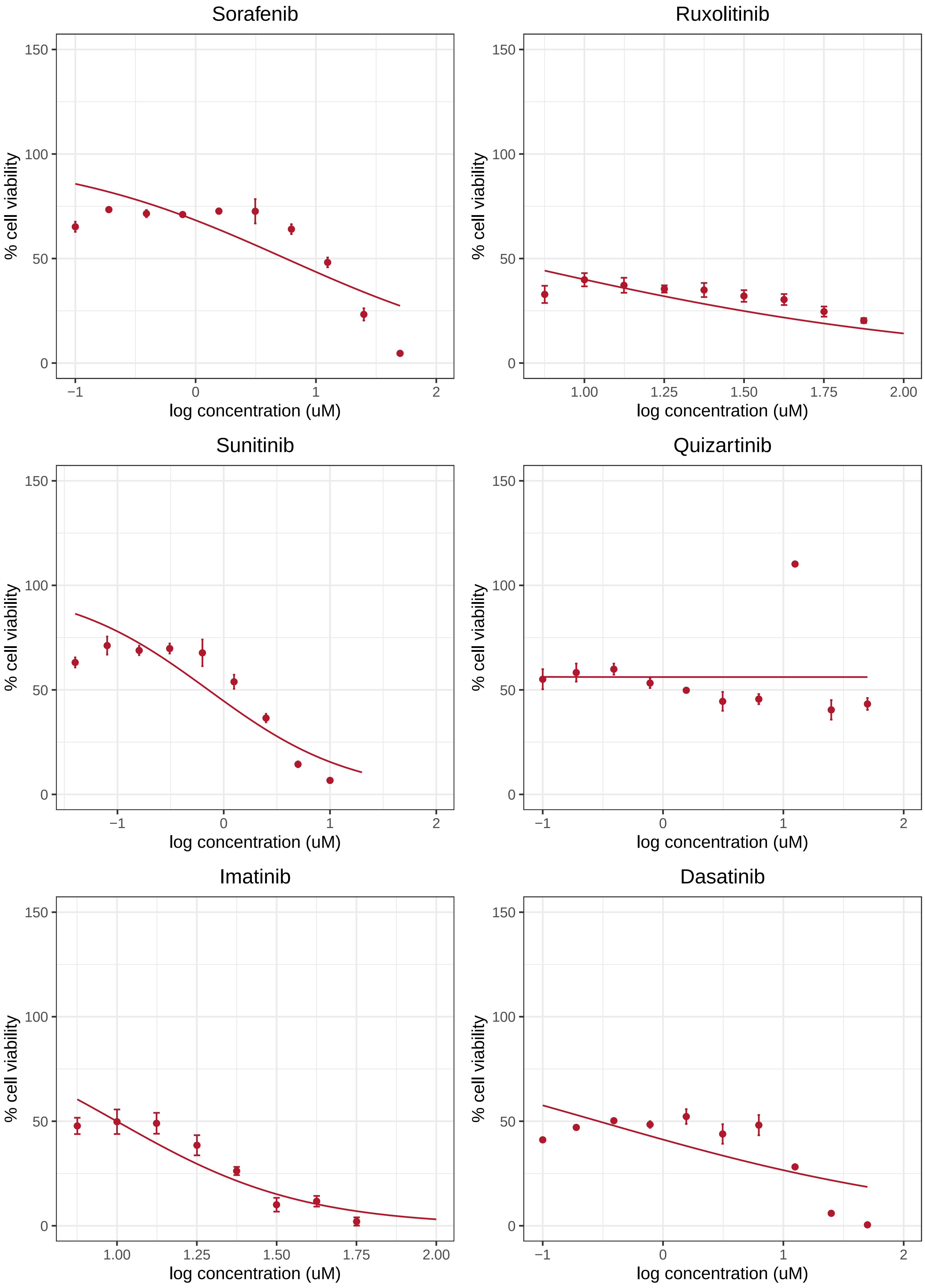

X90376 (subtype=primitive)

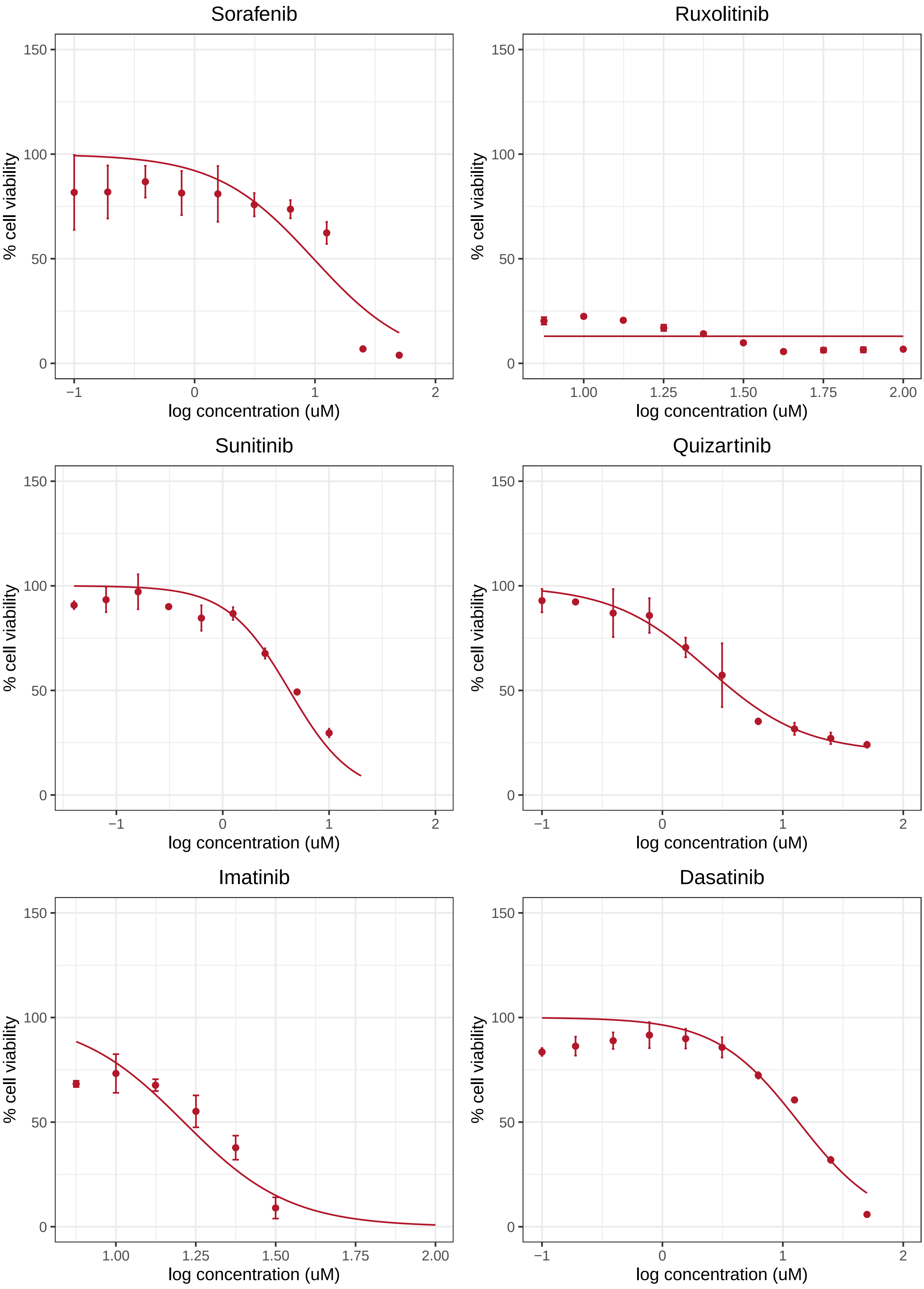

X90669 (subtype=primitive)

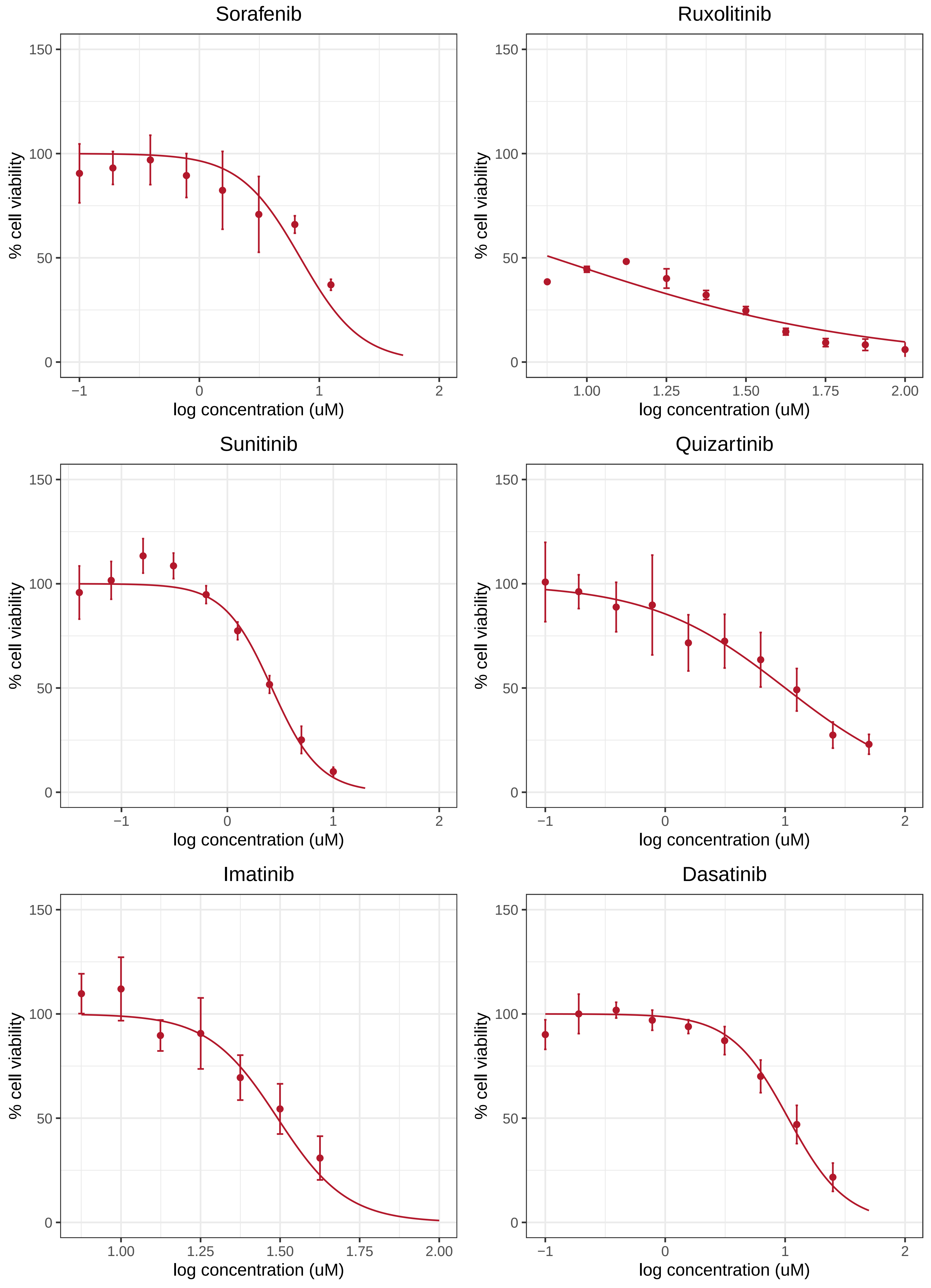

X120541 (subtype=primitive)

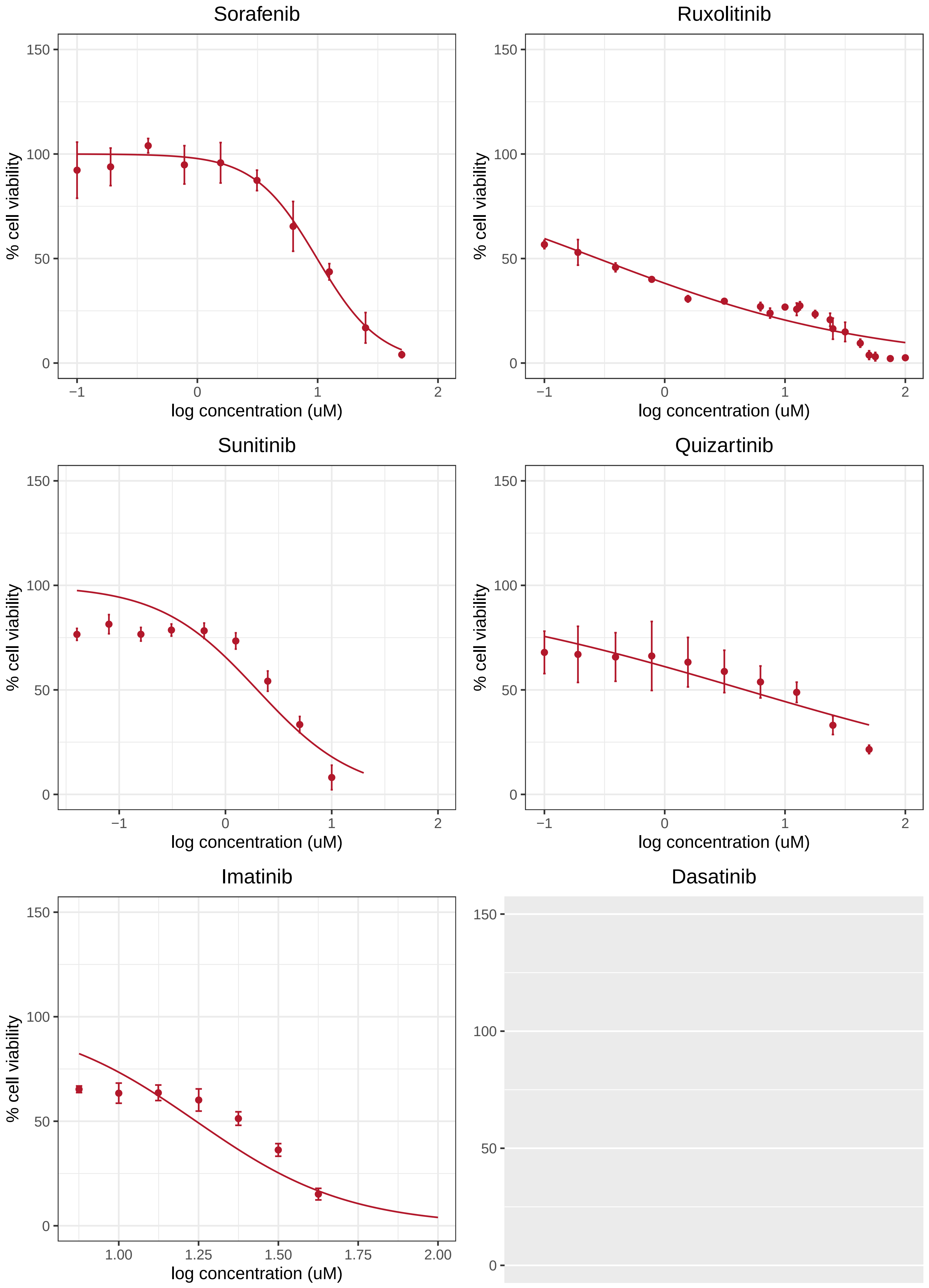

X80561 (subtype=committed)

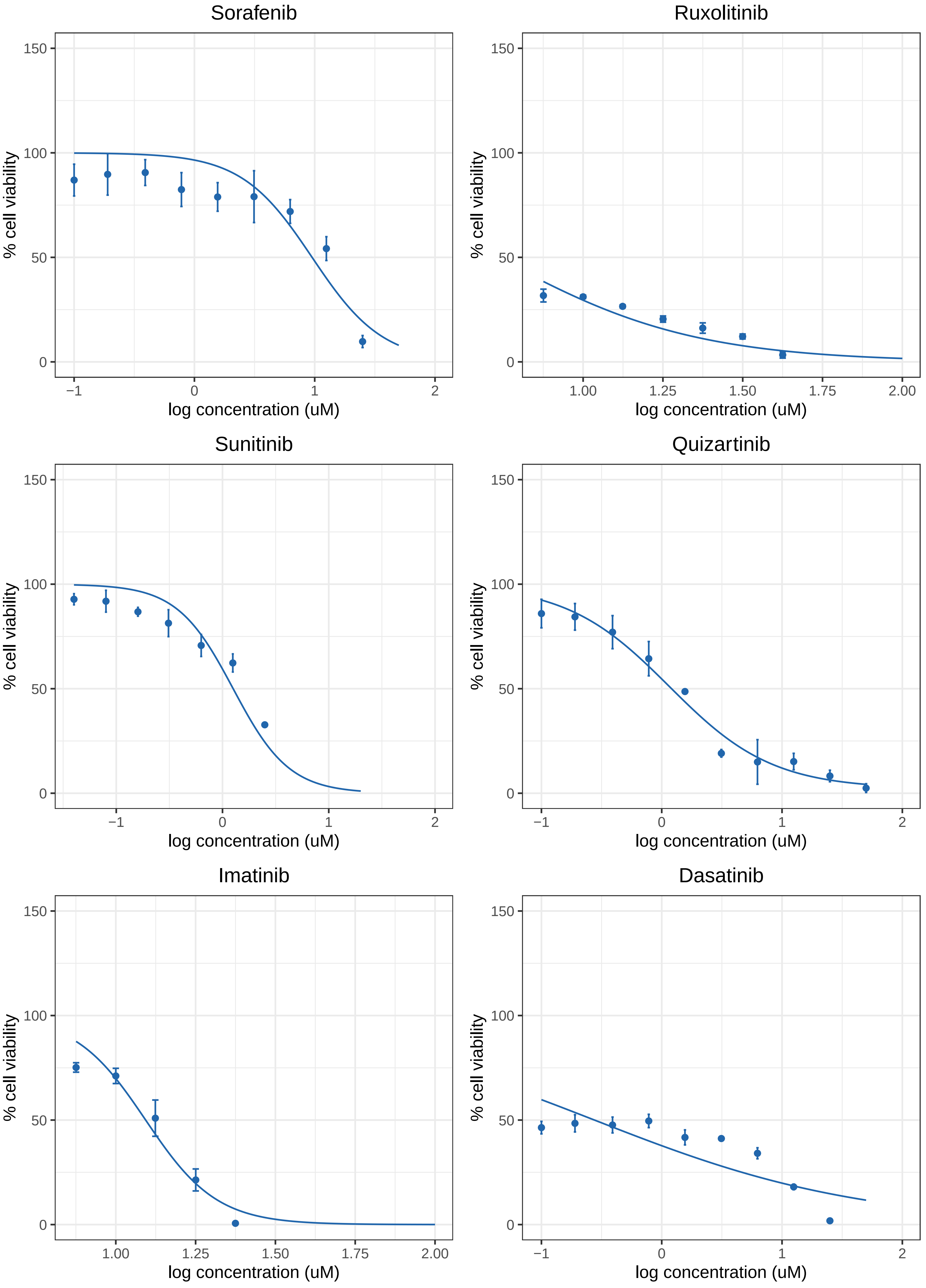

X90202 (subtype=committed)

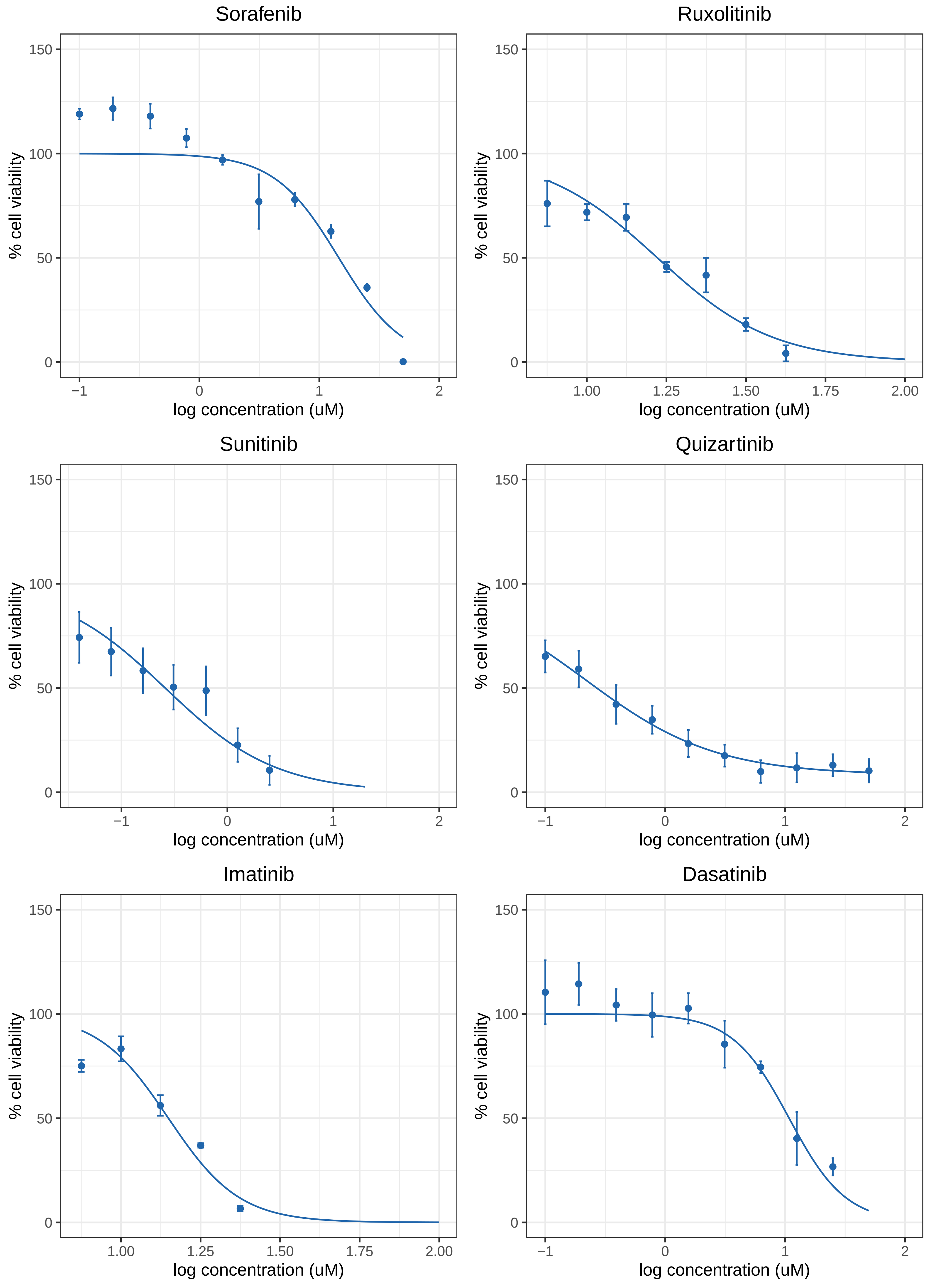

X120942 (subtype=committed)

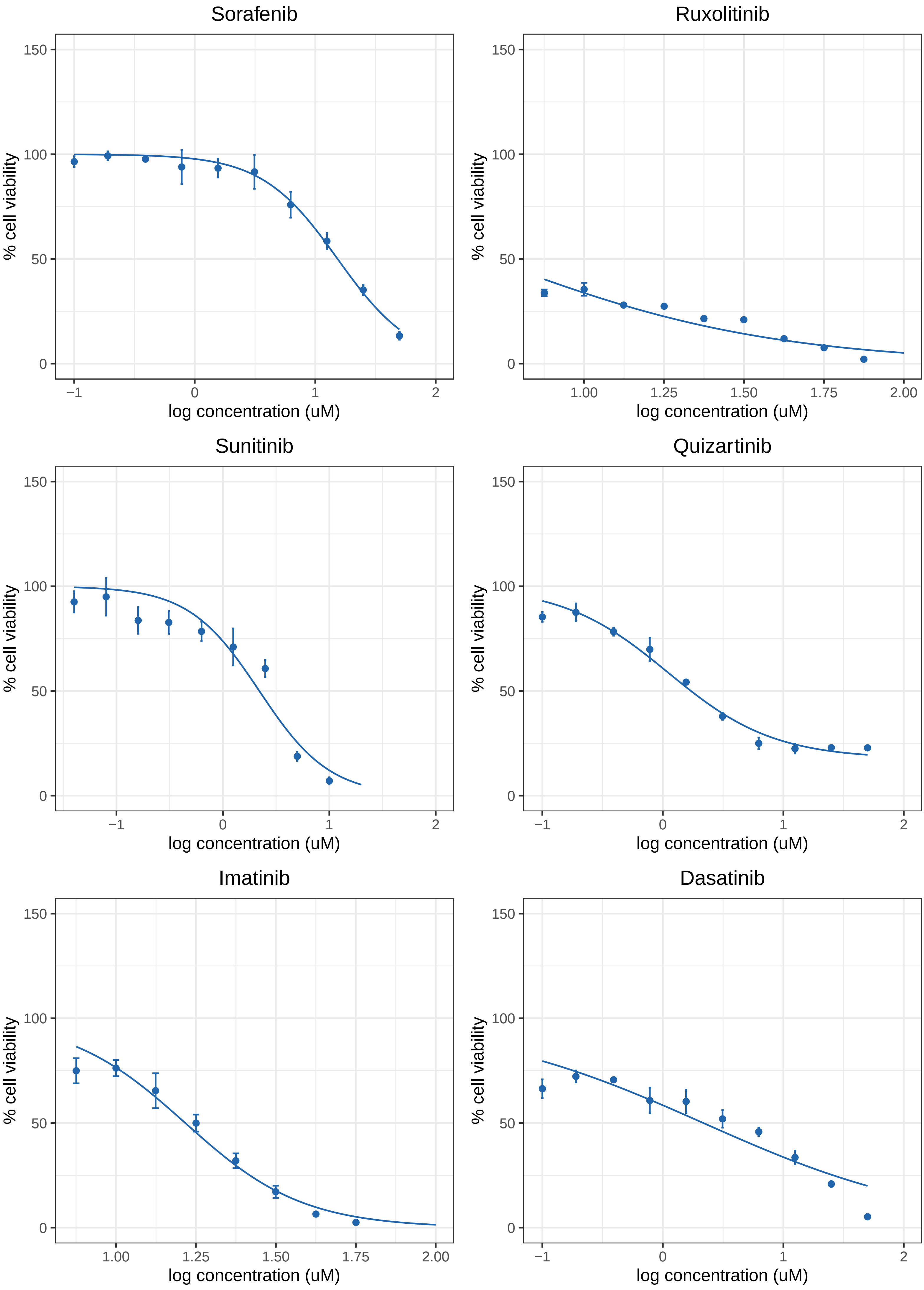

X80197 (subtype=committed)

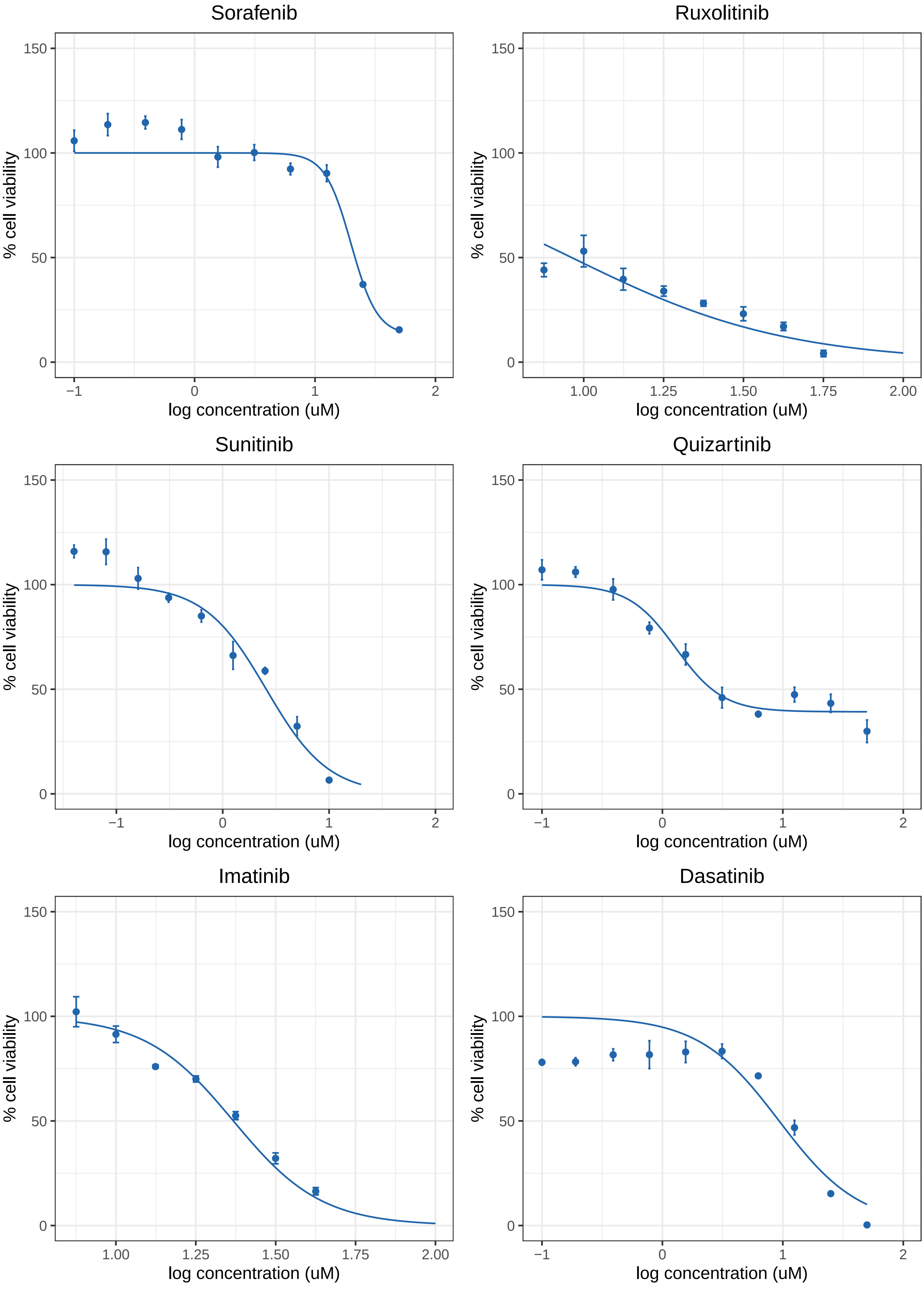

X597 (subtype=committed)

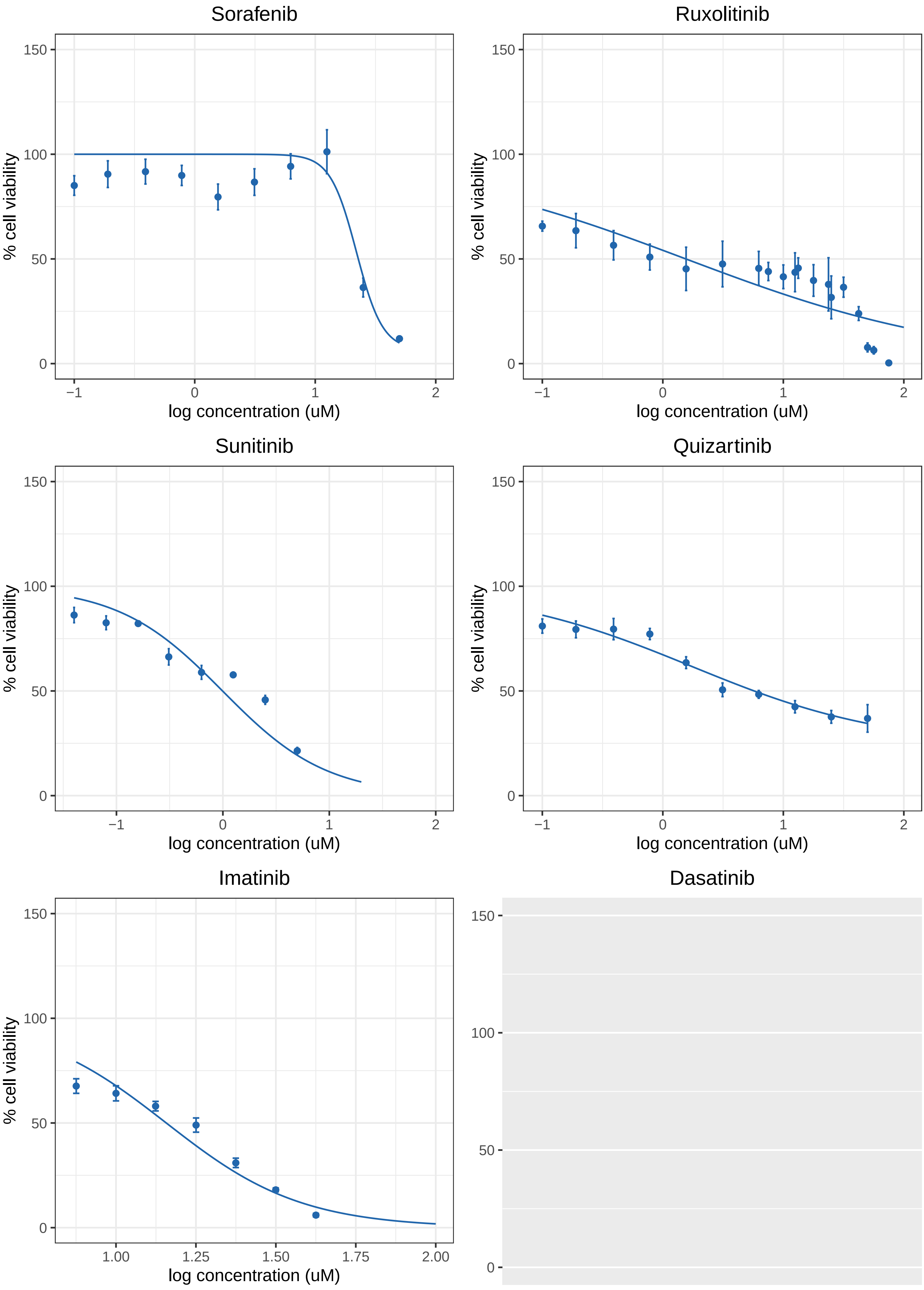

X90784 (subtype=committed)

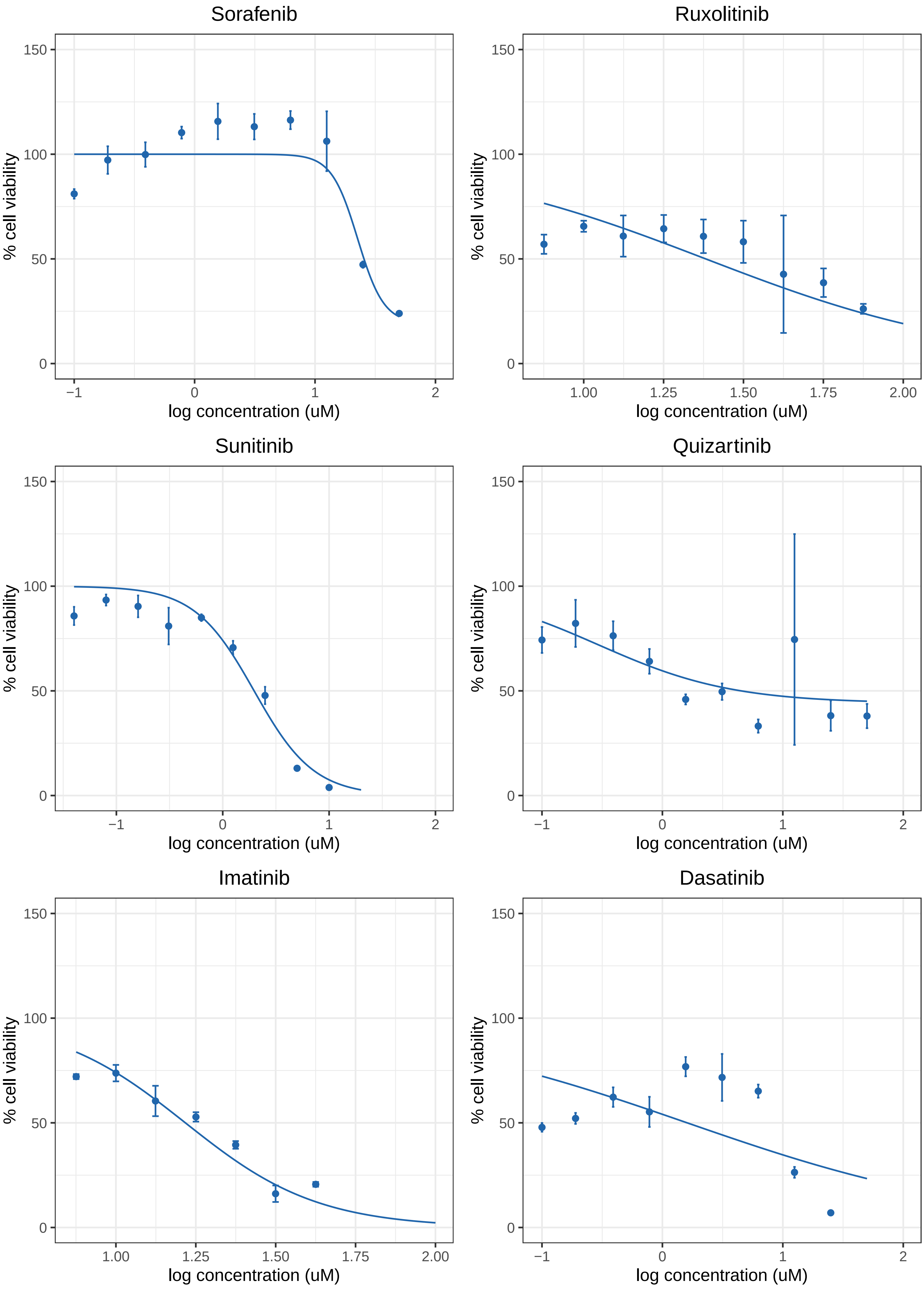

X477 (subtype=committed)

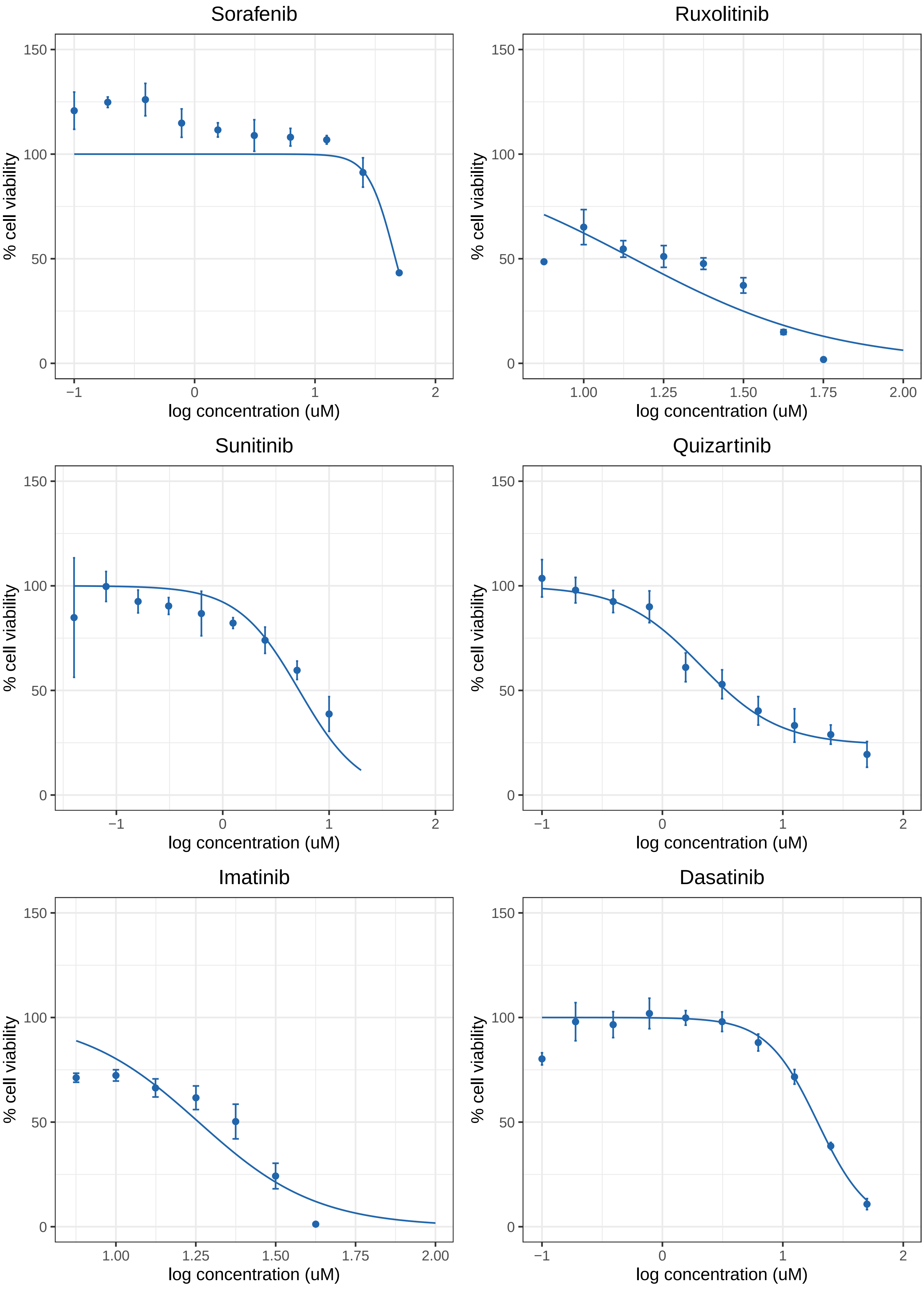

X120805 (subtype=committed)

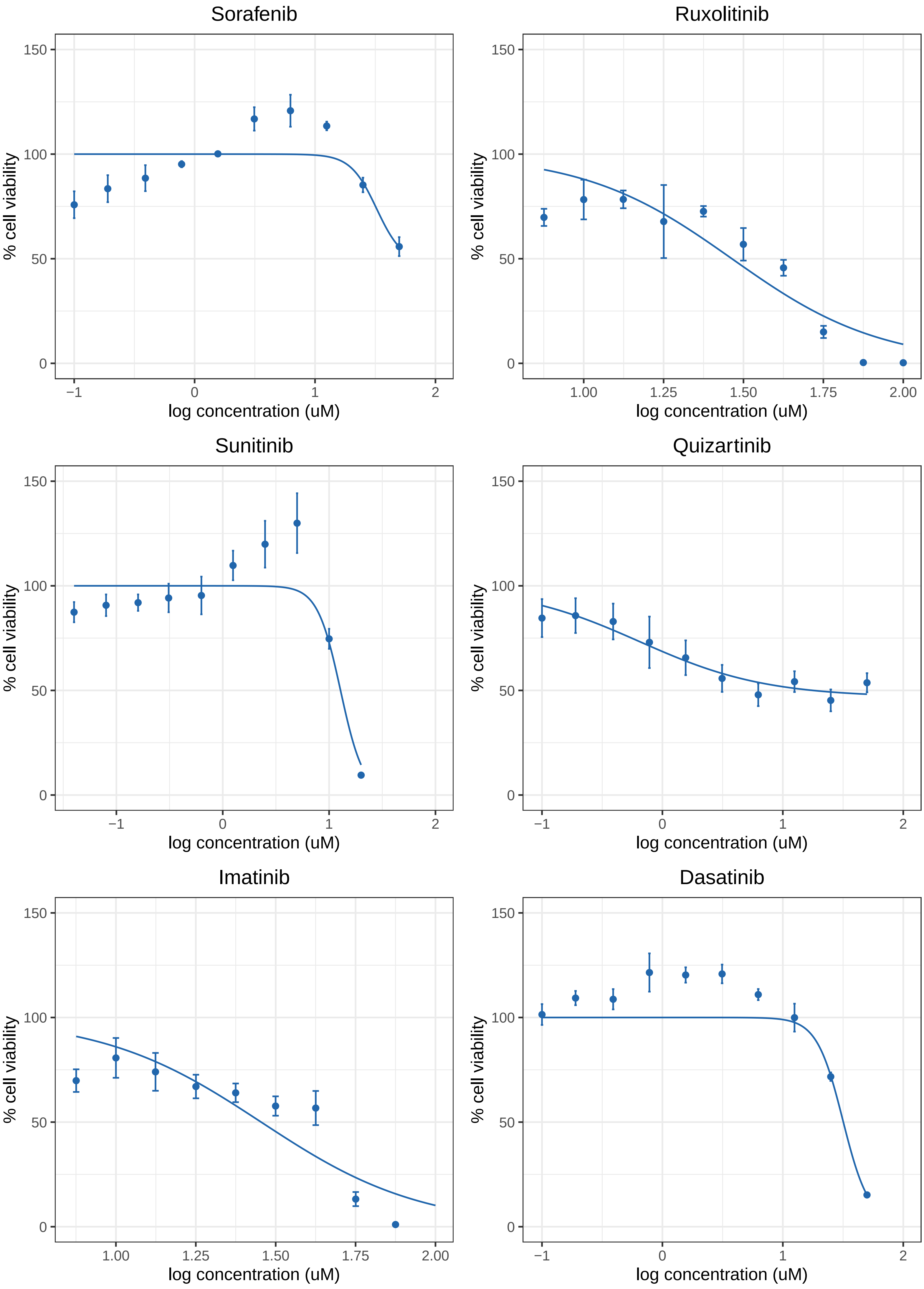

X90047 (subtype=committed)

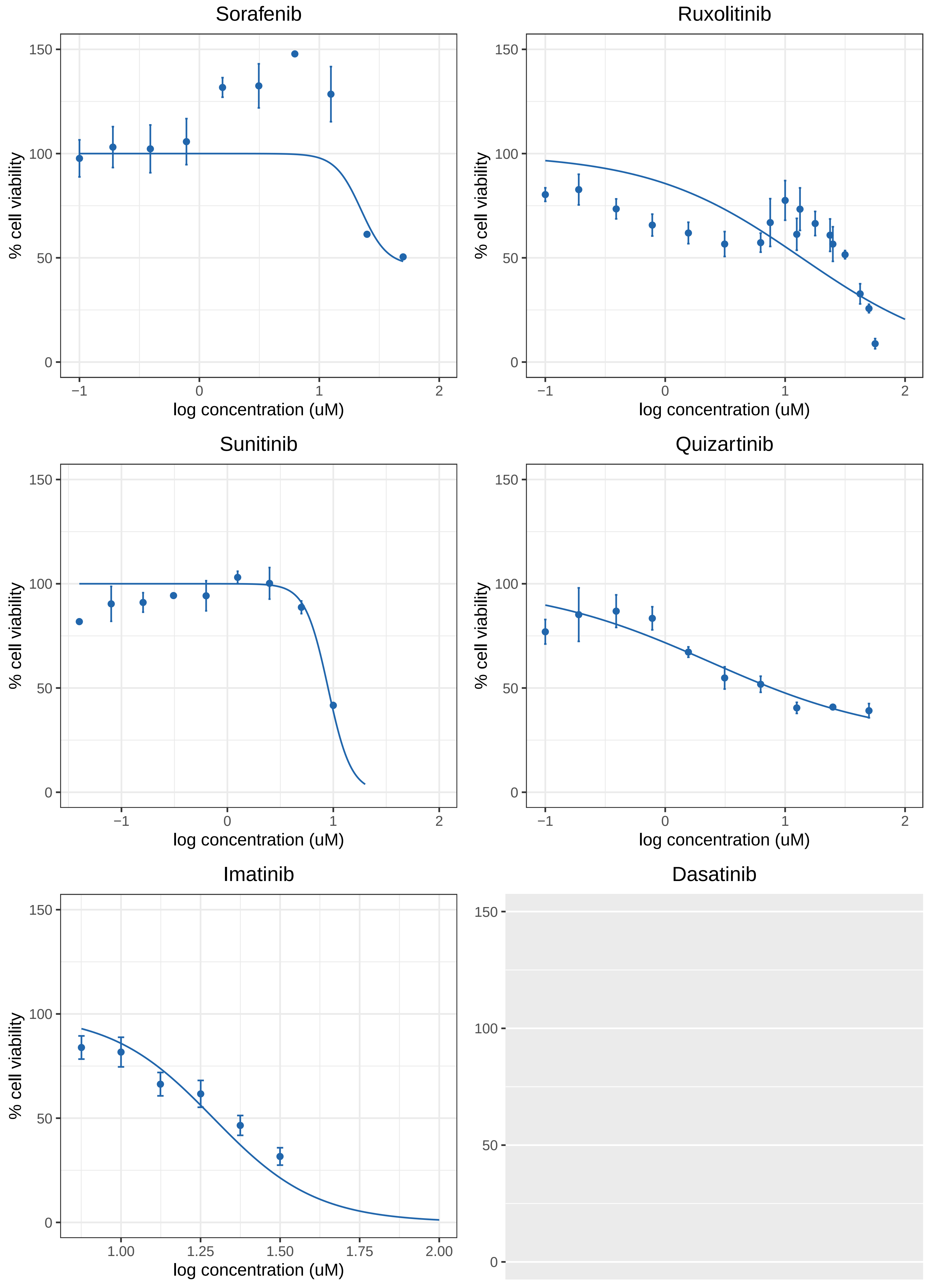

X110530 (subtype=committed)

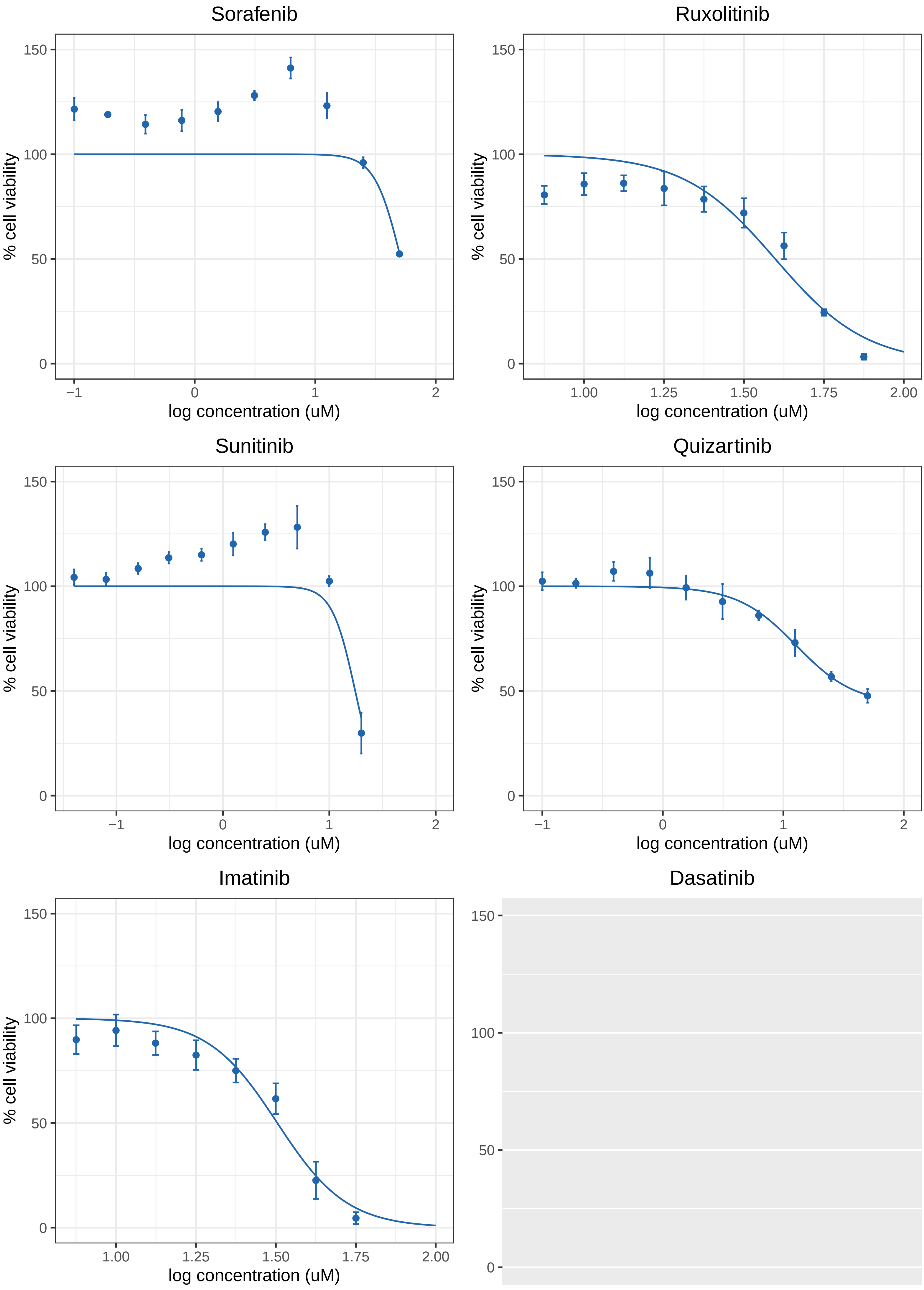

Supplement: Supplementary file 3 — Supplementary Data Files 1–6 [file 41467_2021_21233_MOESM3_ESM.zip › sup_data/Supplementary Data 5.pdf]
