# Supplementary material for: Biological and therapeutic implications of a unique subtype of NPM1 mutated AML
Source: Nat Commun. 2021 Feb 16;12:1054. doi: 10.1038/s41467-021-21233-0 (PMC7886883; doi:10.1038/s41467-021-21233-0)
Supplement: Supplementary file 4 — Reporting Summary [file 41467_2021_21233_MOESM4_ESM.pdf]

nature research

Corresponding author(s): Benjamin Haibe-Kains

Last updated by author(s): Dec 14, 2020

Reporting Summary

Nature Research wishes to improve the reproducibility of the work that we publish. This form provides structure for consistency and transparency in reporting. For further information on Nature Research policies, see our Editorial Policies and the Editorial Policy Checklist.

Statistics

For all statistical analyses, confirm that the following items are present in the figure legend, table legend, main text, or Methods section.

n/a	Confirmed	
☐	☒	The exact sample size (*n*) for each experimental group/condition, given as a discrete number and unit of measurement
☐	☒	A statement on whether measurements were taken from distinct samples or whether the same sample was measured repeatedly
☐	☒	The statistical test(s) used AND whether they are one- or two-sided<br>*Only common tests should be described solely by name; describe more complex techniques in the Methods section.*
☐	☒	A description of all covariates tested
☐	☒	A description of any assumptions or corrections, such as tests of normality and adjustment for multiple comparisons
☐	☒	A full description of the statistical parameters including central tendency (e.g. means) or other basic estimates (e.g. regression coefficient) AND variation (e.g. standard deviation) or associated estimates of uncertainty (e.g. confidence intervals)
☐	☒	For null hypothesis testing, the test statistic (e.g. F, t, r) with confidence intervals, effect sizes, degrees of freedom and P value noted<br>*Give P values as exact values whenever suitable.*
☒	☐	For Bayesian analysis, information on the choice of priors and Markov chain Monte Carlo settings
☒	☐	For hierarchical and complex designs, identification of the appropriate level for tests and full reporting of outcomes
☐	☒	Estimates of effect sizes (e.g. Cohen's d, Pearson's r), indicating how they were calculated

Our web collection on statistics for biologists contains articles on many of the points above.

Software and code

Policy information about availability of computer code

Data collection	The sequencing data generated in this project are available at the European Genome-Phenome Archive (https://ega-archive.org) under the accession id EGAD00001006669 for RNAseq and EGAD00001006670 for ATACseq data. Four other data sets are available at these sources:<br>- TCGA AML data https://portal.gdc.cancer.gov<br>- BeatAML https://doi.org/10.1038/s41586-018-0623-z<br>- KI AML data ttps://doi.org/10.5281/zenodo.292986<br>- Leucegene NCBI-GEO accession ids GSE49642, GSE52656, GSE62190, GSE66917, GSE6703
Data analysis	A complete software environment through CodeOcean containing all necessary data and code to reproduce the analysis and figures described in this manuscript is available at https://doi.org/10.24433/CO.3416958.v1 and https://github.com/bhklab/NPM1-AML .<br>Tools used for the analysis:<br>RNAseq - Kallisto version 0.45.0, HTSeq count version 0.6.1, DESeq2 package (version 1.18.1)<br>ATAC-seq - BWA version 0.7.17, CREAM (version 1.1.1)<br>CyTOF analysis - Helios software (v6.7.1014) , flowCore R package (version 2.0.1), diffcyt R package (version 1.8.6)

For manuscripts utilizing custom algorithms or software that are central to the research but not yet described in published literature, software must be made available to editors and reviewers. We strongly encourage code deposition in a community repository (e.g. GitHub). See the Nature Research guidelines for submitting code & software for further information.

Data

Policy information about availability of data

All manuscripts must include a data availability statement. This statement should provide the following information, where applicable:

- Accession codes, unique identifiers, or web links for publicly available datasets
- A list of figures that have associated raw data
- A description of any restrictions on data availability

- UHN AML RNA-seq data EGAD00001006669, and UHN ATAC-seq data EGAD00001006670
- TCGA https://portal.gdc.cancer.gov
- BeatAML https://doi.org/10.1038/s41586-018-0623-z
- KI https://doi.org/10.5281/zenodo.292986
- Leucegene NCBI-GEO accession ids GSE49642, GSE52656, GSE62190, GSE66917, GSE67039
- Source data for figures are provided as a docker image at https://doi.org/10.24433/CO.3416958.v1 and https://github.com/bhklab/NPM1-AML.

Field-specific reporting

Please select the one below that is the best fit for your research. If you are not sure, read the appropriate sections before making your selection.

☒ Life sciences ☐ Behavioural & social sciences ☐ Ecological, evolutionary & environmental sciences

For a reference copy of the document with all sections, see nature.com/documents/nr-reporting-summary-flat.pdf

Life sciences study design

All studies must disclose on these points even when the disclosure is negative.

Sample size	All of the datasets used in our study are publicly available. We did not perform any computation to determine sample size.
Data exclusions	No data was excluded in this study
Replication	The reproducibility of the experiments was confirmed using at least three biological replicates or replicates involving samples from multiple different patients, as follows: Fig1, Fig2a and Fig5 are representative of 378 patients from 5 different cohorts. Fig2b is representative of 6 patient samples, each with 3 replicates. Fig3 and Fig6 (UHN cohort) are representative of 20 samples. Fig4 is representative of 17 patient samples. Fig6 also represents validation of findings in an independent patient dataset.
Randomization	Randomized studies were not included as we utilized all available data to maximize the sample size. For ATACseq, CyTOF and drug screening analysis samples were chosen based on material availability, mutation and subtype status.
Blinding	Researchers performing RNAseq, ATACseq, CyTOF, drug sensitivity and PCR experiments did not have access to the subtype and mutation status of the samples. However, by necessity the individual on the team doing data analysis did have access to the key regarding the identity of the samples. Steps were taken to prevent bias including naming the samples by a strictly numerical system that does not indicate the genotype of the samples.

Reporting for specific materials, systems and methods

We require information from authors about some types of materials, experimental systems and methods used in many studies. Here, indicate whether each material, system or method listed is relevant to your study. If you are not sure if a list item applies to your research, read the appropriate section before selecting a response.

Materials & experimental systems

n/a	Involved in the study
☐	☒ Antibodies
☐	☒ Eukaryotic cell lines
☒	☐ Palaeontology and archaeology
☒	☐ Animals and other organisms
☐	☒ Human research participants
☒	☐ Clinical data
☒	☐ Dual use research of concern

Methods

n/a	Involved in the study
☒	☐ ChIP-seq
☒	☐ Flow cytometry
☒	☐ MRI-based neuroimaging

Antibodies

Antibodies used	HI30 (marker: CD45; Fluidigm; catalog number: 3089003B); L243 (marker: HLA-DR; BioLegend; catalog number: 307651); SK3 (marker: CD4; BioLegend; catalog number: 344602); 581 (marker: CD34; BioLegend; catalog number: 343502); HIB19 (marker: CD19;

	eBioscience; catalog number: 16-0199-85); M5E2 (marker: CD14; BioLegend; catalog number: 301802); Bu15 (marker: CD11c ; BioLegend; catalog number: 337202); 3G8 (marker: CD16; BioLegend; catalog number: 302002); HIT2 (marker: CD38; BioLegend; catalog number: 303502); WM53 (marker: CD33; BioLegend; catalog number: 303419); NCAM16.2 (marker: CD56; BD Biosciences; catalog number: 559043); UCHT1 (marker: CD3; BioLegend; catalog number: 300402)
Validation	All antibodies have been widely used in the research community and validated by the supplier. A full list with all references to catalogue numbers is provided in supplementary information file 7.

Eukaryotic cell lines

Policy information about cell lines

Cell line source(s)	DSMZ, Braunschweig, Germany
Authentication	Cell lines were authenticated by the supplier.
Mycoplasma contamination	All cell lines were routinely tested for mycoplasma. No contamination was detected.
Commonly misidentified lines (See ICLAC register)	ICLAC-00248 (OCI-AML2) and ICLAC-00249 (OCI-AML3) cell lines were used for RNAseq comparison.

Human research participants

Policy information about studies involving human research participants

Population characteristics	Primary human AML samples were isolated from fresh bone marrow and peripheral blood samples from 77 patients at the diagnosis and prior to therapy or bone marrow transplant. All samples in the UHN cohort have a NPM1 mutation. They were 50 female and 27 male. For each patient written informed consent was obtained in accordance with the Declaration of Helsinki and University Health Network (UHN) institutional review board. All available characters are reported in Supplementary Table 2-6.
Recruitment	Patients were admitted to the Princess Margaret Cancer Centre for examination and treatment for AML. All patients that consented were included in the study.
Ethics oversight	The study was approved by the ethical committee of the University Health Network (UHN) , Toronto Canada.

Note that full information on the approval of the study protocol must also be provided in the manuscript.
